# Potential Driven Spin-Crossover Synergized with Hydrogen-Bond Engineering in a Molecular Catalyst for Selective H_2_O_2_ Electrosynthesis

**DOI:** 10.1021/jacs.6c16940

**Published:** 2026-09-14

**Authors:** Libo Sun, Yanjie Zhai, Zhiying Wu, Dongxue Yu, Zhiqiang Liang, Zhen-An Qiao, Hua Zhang, Xin Wang

**Affiliations:** † Department of Chemistry, 53025 City University of Hong Kong, Kowloon 999077, Hong Kong SAR, P. R. China; ‡ State Key Lab of Inorganic Synthesis and Preparative Chemistry, College of Chemistry, 12510 Jilin University, Changchun 130012, P. R. China; § Hong Kong Institute for Clean Energy, City University of Hong Kong, Kowloon 999077, Hong Kong SAR, P. R. China; ∥ Shenzhen Research Institute, City University of Hong Kong, Shenzhen 518057, P. R. China; ⊥ Hong Kong Branch of National Precious Metals Materials Engineering Research Center, City University of Hong Kong, Kowloon 999077, Hong Kong SAR, P. R. China

## Abstract

The electrochemical two-electron oxygen reduction reaction (2e^–^ ORR) provides a sustainable route for hydrogen peroxide (H_2_O_2_) production. However, its efficiency is fundamentally constrained by the spin-forbidden activation of triplet O_2_ and the subsequent proton-coupled electron-transfer steps required to form singlet H_2_O_2_. Herein, we report a pyrazine-functionalized nickel phthalocyanine catalyst (NiPNPC) that overcomes these constraints and achieves exceptional selectivity for H_2_O_2_ (up to 97.15%). In situ spectroscopic and computational studies reveal that the pyrazine units within the macrocyclic ligand promote a dynamic spin-crossover at the Ni center from a low-spin to a high-spin under cathodic polarization for O_2_ activation, thereby resolving the intrinsic spin mismatch. Concurrently, the pyrazine units facilitate the reorganization of a well-defined hydrogen-bonding network at the electrolyte-catalyst interface. This network ensures efficient proton delivery to the active center and thereby efficient H_2_O_2_ formation. The synergy between dynamic spin-state modulation and interfacial hydrogen-bond engineering provides a mechanistic understanding for selective H_2_O_2_ formation and establishes a new design paradigm for advanced molecular electrocatalysts in sustainable synthesis.

## Graphical abstract

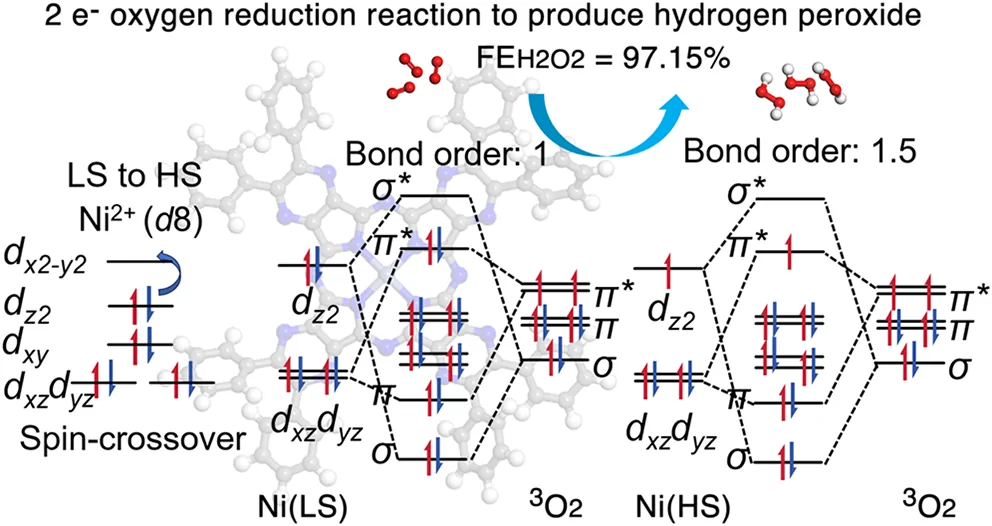

## Introduction

Hydrogen peroxide (H_2_O_2_) is a vital oxidant with extensive industrial applications.^1–3^ Currently, its large-scale production is dominated by the energy-intensive anthraquinone process, which faces challenges such as complex operations, environmental issues, and high costs associated with the catalysts.^4^ These drawbacks have created a growing demand for more efficient and sustainable alternatives.^5–11^ The electrochemical two-electron oxygen reduction reaction (2e^–^ ORR) has emerged as a promising route for H_2_O_2_ synthesis.^12–15^ However, achieving high efficiency and selectivity remains a major challenge, primarily due to the competing four-electron pathway that produces water.^16–20^ Overcoming this selectivity bottleneck requires the development of advanced ORR catalysts.^21–25^ Therefore, a fundamental understanding of the reaction mechanism and the design of rational catalysts are crucial for advancing this sustainable production technology.^26–31^

A fundamental challenge in efficient 2e^–^ ORR lies in its spin-forbidden character, where the ground-state molecular oxygen (^3^O_2_) is in a triplet, and H_2_O_2_ is in a singlet.^32–34^ This spin mismatch creates an inherent barrier to the 2e^–^ ORR pathway.^35^ Molecular catalysts, such as metal phthalocyanines, serve as an ideal platform to address this issue due to their tunable structures.^36–41^ Currently, conventional design strategies have primarily focused on optimizing static properties, such as the choice of the metal center or the use of electron-withdrawing/donating functional groups to decorate the macrocyclic ligands, etc.^24,42^ In contrast, the dynamic evolution of the molecular catalyst’s geometric and electronic structure and spin state during catalysis, especially how ligand modifications influence this dynamic reaction process, remains poorly understood.^43,44^

Beyond the spin challenge, the overall selectivity towards H_2_O_2_ is further dictated by subsequent proton-coupled electron transfer (PCET) steps.^45–47^ The efficiency of these PCET processes is highly dependent on the local reaction environment, especially the hydrogen-bonding network at the catalyst-electrolyte interface.^48^ Engineering the macrocyclic ligand, for example, by incorporating hydrogen-bonding motifs onto the backbone, offers a viable strategy to tailor this interfacial microenvironment.^49^ An optimized hydrogen-bonding network can facilitate proton delivery, thereby aiding the formation and stabilization of H_2_O_2_.^50–53^ Nonetheless, a fundamental question persists about how such engineered interfacial hydrogen-bonding networks cooperate with the dynamic spin and electronic states of the active sites to drive the 2e^–^ ORR pathway. This mechanistic interplay remains a pivotal yet underexplored aspect of molecular catalysts.

Herein, we demonstrate that the combined strategy of facilitating dynamic spin-crossover and engineering an interfacial hydrogen-bonding network in a molecular catalyst drives highly selective H_2_O_2_ electrosynthesis.^54^ Using a pyrazine-functionalized nickel phthalocyanine derivative (NiPNPC) as the model catalyst, we achieve H_2_O_2_ selectivity up to 97.15%. In situ spectroscopic and theoretical analyses reveal that the pyrazine units in NiPNPC allow the nickel site, initially in a low-spin state in the pristine catalyst, to undergo a facilitated dynamic transition to a high-spin state to activate triplet O_2_. This spin-crossover facilitates the formation of a superoxide intermediate, thereby overcoming the spin-forbidden nature of O_2_ reduction. Concurrently, the pyrazine units in the ligand framework facilitate the formation of a tailored hydrogen-bonding network at the catalyst-electrolyte interface. This network enables efficient proton delivery during subsequent PCET steps and steers the reaction toward H_2_O_2_. Our work elucidates a cooperative mechanism in which dynamic spin-state transition activates triplet O_2_ to form a superoxide intermediate, while an engineered hydrogen-bonding microenvironment facilitates the crucial protonation steps. This insight advances the catalyst design paradigm from static electronic and geometric tuning toward the active co-regulation of spin states and interfacial microenvironments, providing a new strategy for developing molecular electrocatalysts for sustainable synthesis.

## Results and Discussion

Building on the recognition that nickel phthalocyanine (NiPC) derivatives are promising candidates for the 2e^–^ ORR, our previous work primarily explored static ligand modifications to influence the Ni active site.^38^ To advance beyond this strategy and address the dynamic and interfacial factors highlighted in the above section, we turned to pyrazine as a functional unit.^55^ Pyrazine provides structural rigidity, electronic tunability, and the ability to mediate both metal center properties and intermolecular interactions.^56^ The incorporation of pyrazine into the NiPC framework serves a dual purpose. First, it modulates the electron density at the Ni center, potentially influencing its dynamic spin state under reaction conditions. Second, the nitrogen atoms within the pyrazine ring offer sites to reorganize the hydrogen-bonding network at the catalyst-electrolyte interface. This integrated design targets the simultaneous regulation of dynamic spin states and the local proton transfer microenvironment.

Guided by this design principle, we synthesized a series of NiPC derivatives functionalized with pyrazine units, including NiNPC and NiPNPC, alongside the control counterpart NiPhPC, which lacks this unit (Figure 1b). We first investigated their electronic structures through computational methods. Analysis of the calculated electrostatic potential (ESP) maps reveals that the pyrazine unit significantly alters the molecular surface electrostatic potential (Figure 1c).^57^ In NiNPC and NiPNPC, the pyrazine regions appear blue, indicating a high electron density and an electron-rich state. This feature suggests a strong affinity for the partially positive hydrogen atoms of surface water molecules. Furthermore.^58^ Molecular orbital calculations further demonstrate a narrowing of the energy gap between the highest occupied molecular orbital (HOMO) and the lowest unoccupied molecular orbital (LUMO) in the order of NiNPC > NiPNPC > NiPhPC (Figure 1d). The difference in the HOMO-LUMO gap implies different electron transfer capabilities.^38^ These computational results confirm that the incorporation of pyrazine effectively tunes the electronic properties of the Ni active site and promotes surface water reorganization at the molecular level.

**Figure 1:**
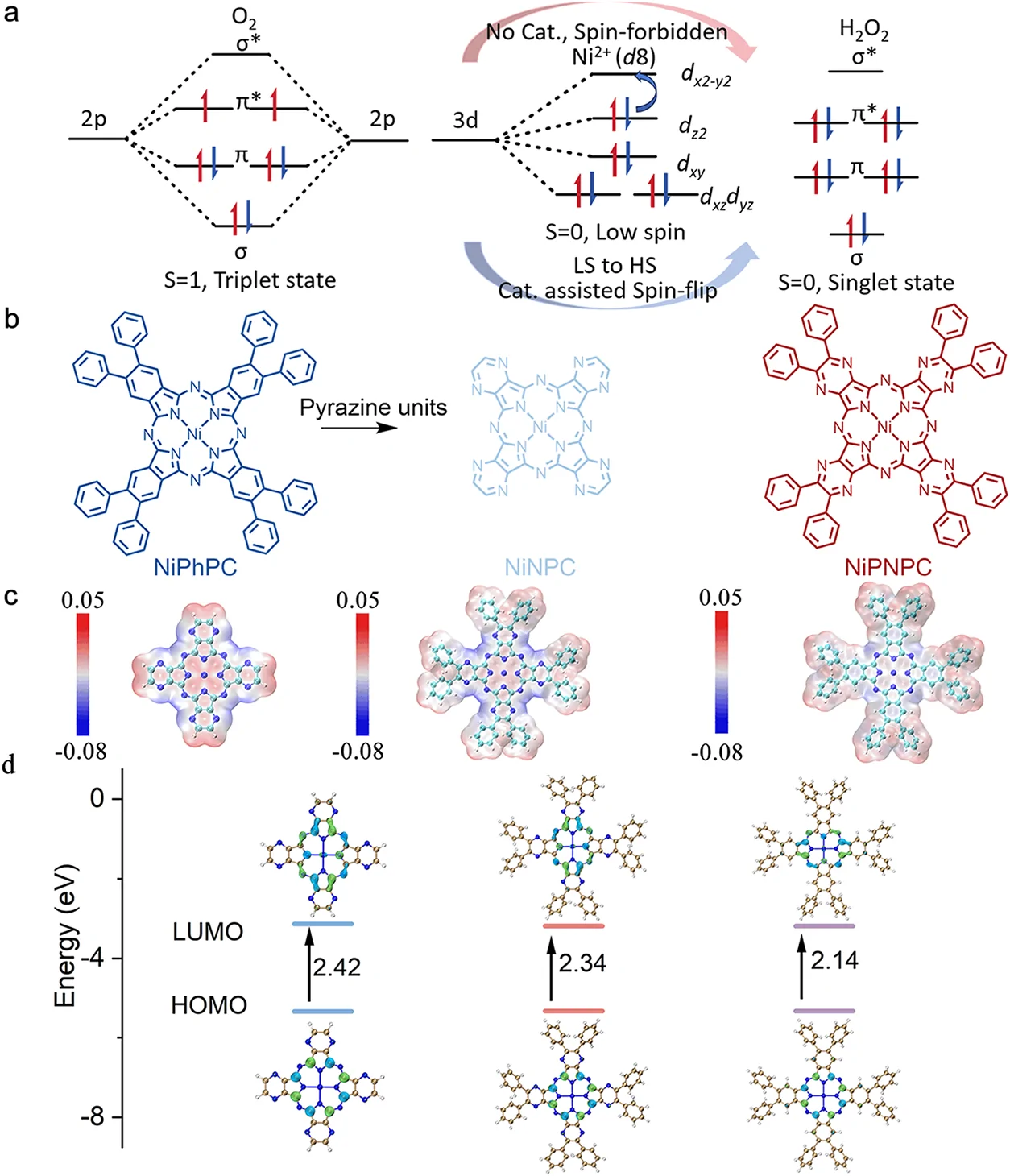
(a) Illustrating the triplet state O_2_ and the singlet state H_2_O_2_. (b) Illustrating the structure of NiNPC and NiPNPC. (c) Calculated electrostatic potentials (ESP) for the NiPC derivatives. (d) Calculated HOMO–LUMO orbitals.

Following the computational design, the target NiPC derivatives (NiNPC, NiPNPC, and NiPhPC) were successfully synthesized (see Experimental Section for details), and their molecular structures were confirmed by high-resolution mass spectrometry (HRMS, Figure S1). The electronic structures were further corroborated by UV–vis spectroscopy (Figure S2). All compounds display the characteristic absorption profiles of methallophthalocyanines, with a B-band (Soret band) around 290–320 nm and a Q-band around 550–700 nm, corresponding to a π–π* transitions.^43^ Notably, NiPNPC exhibits a slight red shift in its Q-band absorption compared to NiNPC, indicating a reduced HOMO–LUMO gap and facilitated π–π* transition. NiPhPC exhibits a further red shift than NiPNPC. This observation aligns well with the trend predicted by the frontier orbital calculations.

The oxidation state and local coordination environment of nickel in the synthesized derivatives were elucidated by X-ray photoelectron spectroscopy (XPS) and X-ray absorption near-edge structure (XANES) spectroscopy. The Ni 2p XPS spectra (Figure 2a) for NiNPC, NiPNPC, and NiPhPC all show characteristic peaks of 2p_3/2_ and 2p_1/2_ at around 855.4 and 872.8 eV, confirming a nearly Ni­(II) oxidation state.^59^ The subtle variations in these spectral profiles indicate that the different functional groups modulate the electronic structure of these Ni centers. This finding is further supported by Ni K-edge XANES (Figure 2b). The edge positions for all derivatives align closely with those of the commercial NiPC, confirming a slightly less than +2 oxidation state.^60^ These results verify the successful synthesis of the target molecular structures, consistent with the HRMS and UV–vis data. Fourier-transform extended X-ray absorption fine structure (FT-EXAFS) spectra (Figure 2e) further show a prominent first-shell peak at around 1.4 Å (phase-uncorrected) for NiNPC, NiPNPC, NiPhPC, and commercial NiPC, attributable to Ni–N scattering. The complete absence of a Ni–Ni scattering path (around 2.2 Å) across all series unequivocally verified the isolation status of Ni sites. This conclusion was further corroborated by wavelet transform (WT) analysis (Figure 2c), which displayed only a single maximum at around 6.9 Å^–1^ and around 1.4 Å in the (k, R) space, corresponding to Ni–N coordination. Importantly, no characteristic signals of Ni–Ni metallic bonds were detected across the entire k-space range, particularly around 8.1 Å^–1^. Quantitative EXAFS fitting afforded the precise local coordination parameters (Figure S7 and Table S2). The fitting yielded an average Ni–N bond length of around 1.9 Å and a coordination number of around 4, confirming the four-coordinate configuration of our designed molecular catalysts.

**Figure 2:**
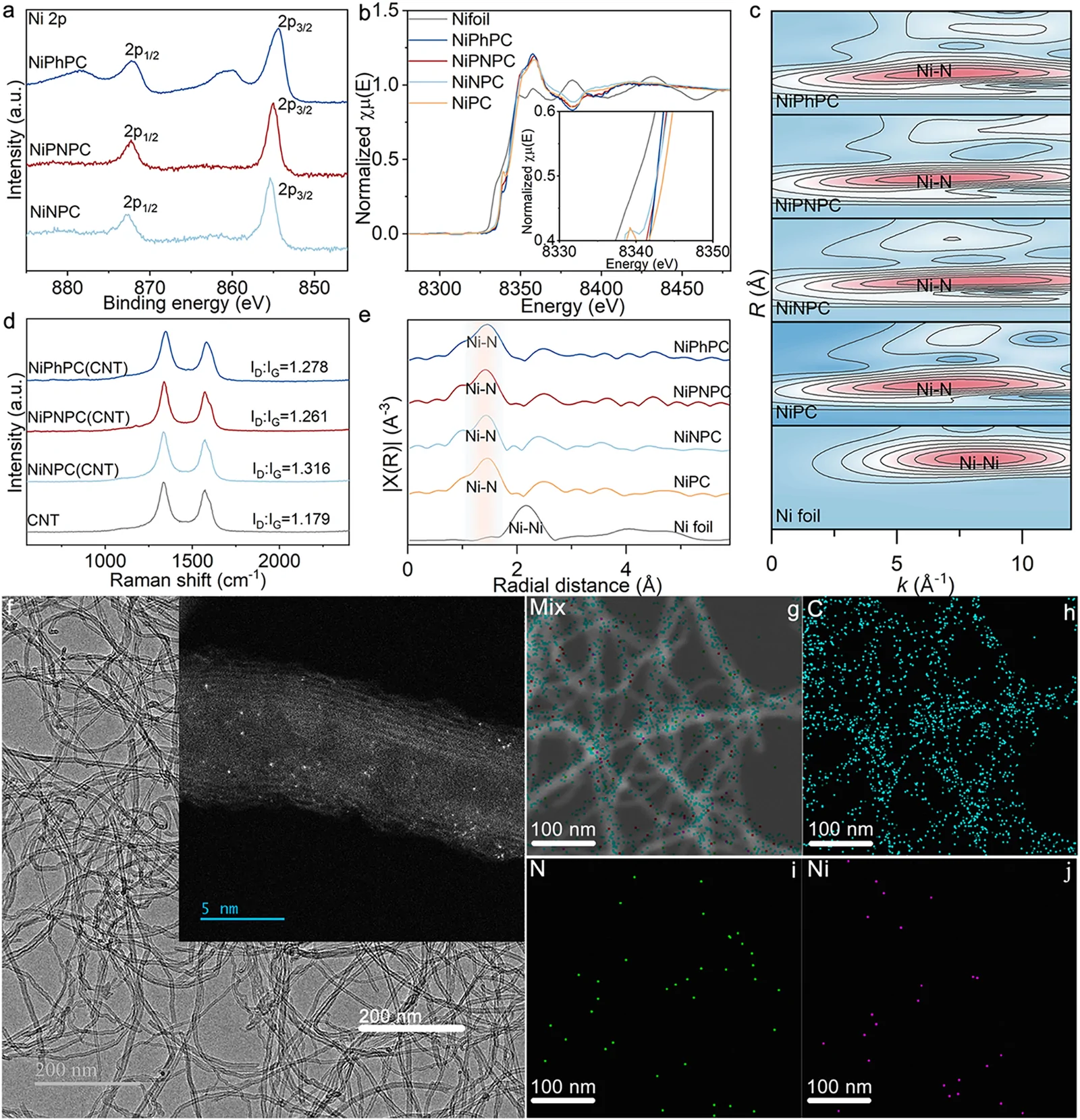
(a) XPS spectra for Ni 2p_3/2_ and 2p_1/2_ peaks. (b) Ni K-edge XANES data. (c) Wavelet transform (WT) contour maps of the extended EXAFS spectra at the Ni K-edge. (d) Raman spectra after immobilization. (e) Fourier-transformed (FT) k^3^-weighted χ­(k) function of the EXAFS spectra. (f) Illustration of transmission electron microscopy (TEM) images of NiPNPC. Inset: AC HAADF-STEM image of NiPNPC. (g–j) Energy-dispersive X-ray spectroscopy mapping images of NiPNPC.

To enable efficient electrocatalytic testing, the molecular NiPC catalysts were heterogenized by immobilizing them on multi-walled carbon nanotubes (CNTs) via π-π stacking interactions. The successful loading was first evidenced by Raman spectroscopy (Figure 2d). The intensity ratio of the *I*_D_/*I*_G_ follows the order NiNPC­(CNT) > NiPhPC­(CNT) > NiPNPC­(CNT). This trend indicates that the functionalized derivatives, particularly NiPNPC and NiPhPC, integrate more effectively with the CNT graphitic surface, promoting a more ordered composite structure. The morphology and distribution of the supported catalysts were further examined by electron microscopy (Figures 2f, and S3–S4). Taking the optimal NiPNPC as an example, high-resolution transmission electron microscopy (HRTEM) and high-angle annular dark-field scanning transmission electron microscopy (HAADF-STEM) images (Figure 2f and inset) confirm the uniform dispersion of NiPNPC molecules on the CNT scaffold without observable aggregation. Elemental mapping via energy-dispersive X-ray spectroscopy (EDS) within the scanning electron microscope (Figure 2g–2j) visually demonstrates the homogeneous distribution of Ni, N, and C elements, corroborating the successful and uniform immobilization. Besides, after immobilization on CNT, the Ni 2p_3/2_ binding energies of NiPhPC, NiNPC, and NiPNPC all converge to approximately 856 eV, indicating similar electronic environments at the Ni centers (Figure S5). Meanwhile, EXAFS fitting confirms that all three catalysts retain the four-coordinate Ni–N_4_ structure on the CNT support (Figures S6 and S8, Table S2).^61,62^ These results suggest that the CNT support does not selectively perturb the Ni centers in a way that would distinguish the three catalysts, and the distinct catalytic behavior likely arises primarily from the molecular structure of the ligands.^54,63^

The electrocatalytic performance of the NiPC derivatives for the 2e^–^ ORR was evaluated using a rotating ring-disk electrode (RRDE). All potentials are reported versus the reversible hydrogen electrode (RHE). Linear sweep voltammetry (LSV) curves were recorded in O_2_-saturated 0.1 M KOH at a rotation rate of 1600 rpm (Figure 3a). The disk current densities (*j*_disk_) correspond to O_2_ reduction, while the ring current (*j*_ring_) arises from the oxidation of generated H_2_O_2_. Among the series, NiPhPC­(CNT) showed higher disk current and a relatively lower ring current over most potential windows, indicating it may also facilitate 4e^‑^ pathway to H_2_O, consistent with its poorer selectivity for H_2_O_2_. In contrast, NiPNPC­(CNT) exhibited a more positive onset potential for both disk and ring currents relative to the other catalysts, suggesting superior intrinsic activity associated with pyrazine units.

**Figure 3:**
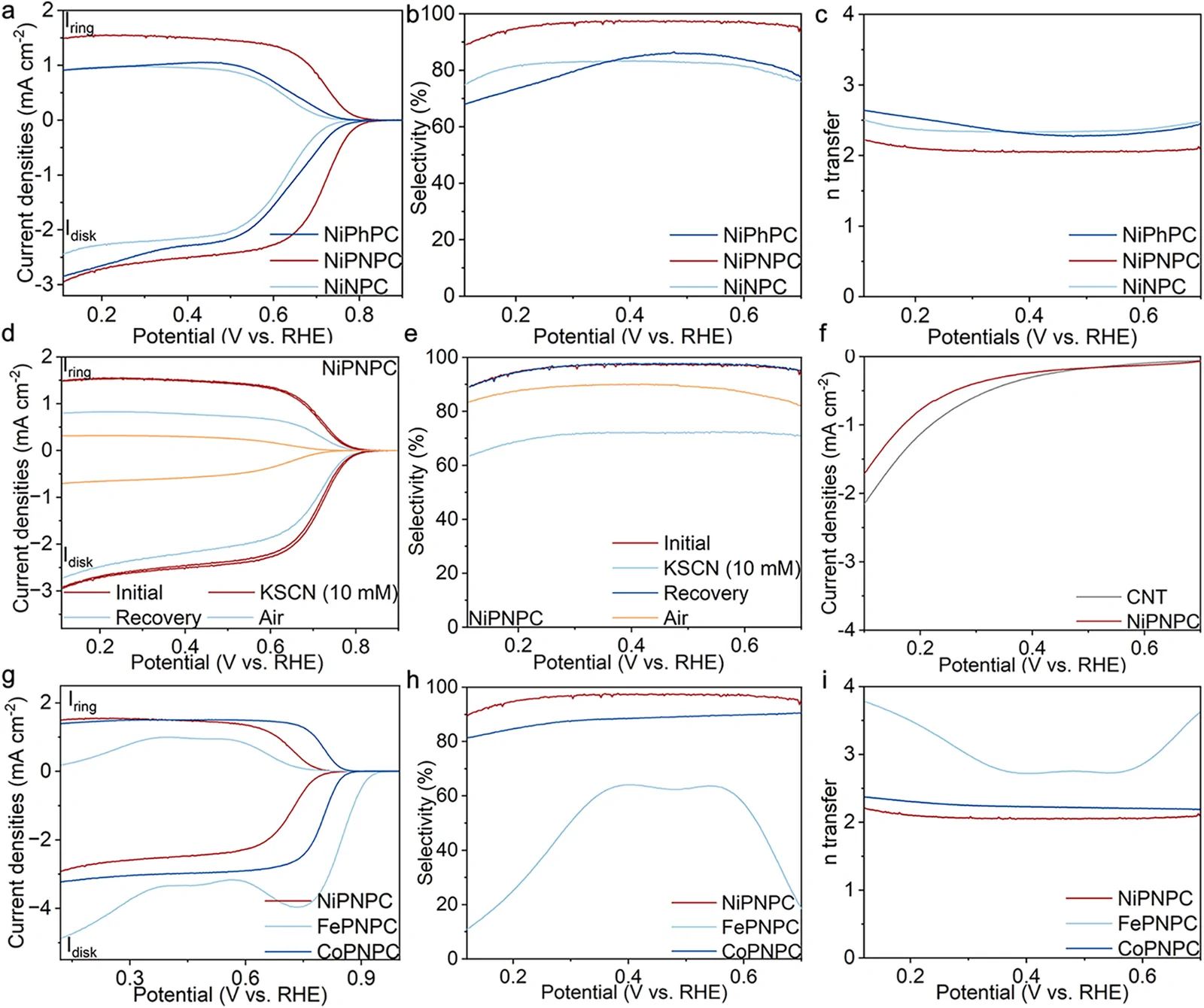
(a) Electrocatalytic LSV curves at the ring-disk electrodes. (b) Calculated selectivity for production of H_2_O_2_. (c) Calculated electron transfer numbers. (d, e) LSV and selectivity comparison of NiPNPC in O_2_ atmosphere, air atmosphere, and with the addition of KSCN. (f) LSV comparison of NiPNPC and bare CNT electrode. (g–i) LSV, selectivity, and n transfer comparison of FePNPC, CoPNPC, and NiPNPC.

The corresponding H_2_O_2_ selectivity and electron transfer number (*n*) were derived from the RRDE data and are presented in Figure 3b,3c. Remarkably, NiPNPC­(CNT) maintains high H_2_O_2_ selectivity (>90%) across a broad potential window (0.1 to 0.7 V vs RHE), reaching a maximum value of 97.15%, significantly higher than the others and bare CNT (Figure S10). Concurrently, its electron transfer number remains below 2.2 over this range, confirming the dominant 2e^–^ pathway.^64^ These results confirm NiPNPC to as the the most intrinsic catalyst for 2e^–^ ORR among the series. Meanwhile, the reaction kinetics of these catalysts were studied (Figure S13), where NiPNPC exhibits the lowest Tafel slopes for both disk (55 mV dec^–1^) and ring electrodes (62 mV dec^–1^), indicating that NiPNPC possesses the fastest ORR and H_2_O_2_ generation kinetics among these catalysts.^65^ To further probe the role of proton transfer in the rate-determining step, we performed Tafel analysis in both H_2_O and D_2_O electrolytes (0.1 M KOH vs 0.1 M KOD, Figure S14). For NiPNPC, the Tafel slope increased from 62 mV dec^–1^ in KOH to 84 mV dec^–1^ in KOD, while for NiPhPC it increased from 81 mV dec^–1^ to 103 mV dec^–1^. The kinetic isotope effect (*k*_H_/*k*_D_) for NiPNPC reached approximately 1.5–2 at cathodic potentials below 0.4 V, significantly higher than that for NiPhPC (approximately 1.5 across almost the entire potential range). This confirms that proton transfer plays a more pronounced role in the rate-determining step for NiPNPC, consistent with the enhanced proton delivery enabled by the pyrazine-reorganized hydrogen-bonding network. The smaller KIE for NiPhPC further supports that its proton transfer is less efficient.

To assess the practicality of NiPNPC under more realistic conditions, we evaluated its performance in an air atmosphere (Figure 3d,e). Although the absolute current densities decreased due to the lower O_2_ concentration, the H_2_O_2_ selectivity remained high (>80%, with a maximum of 90.10%), demonstrating its robust performance in ambient air. To identify the active site, we performed a poisoning experiment by introducing KSCN into the electrolyte (Figures 3d,3e and S15).^64,66^ The marked decrease in both current densities and H_2_O_2_ selectivity upon the addition of SCN^–^ ions, which are known to block the metal sites, confirms the involvement of Ni in the catalytic process. The activity could be almost fully recovered after thoroughly washing the catalyst, elucidating the crucial role of Ni sites in the ORR process. Furthermore, to rule out catalytic decomposition of the produced H_2_O_2_, we conducted control experiments by scanning the potential in an N_2_-saturated electrolyte containing H_2_O_2_. The NiPNPC electrode showed decreased current for H_2_O_2_ reduction compared to bare CNT (Figure 3f), indicating that the H_2_O_2_ produced is not easily decomposed on the catalyst surface.

To underscore the unique advantage of the Ni center in NiPNPC, we synthesized and evaluated analogous Fe- and Co-based phthalocyanines with pyrazine (FePNPC, CoPNPC) and phenyl (FePhPC, CoPhPC) substituents for comparison.^67,68^ As shown in Figures 3g–3i and S16, their catalytic behavior differs markedly. Both FePNPC and FePhPC promote the 4e^‑^ ORR pathway to water and pooer 2e^–^ ORR pathway to H_2_O_2_, consistent with the known properties of Fe centers. In contrast, the Co-based analogues (CoPNPC and CoPhPC) favor the 2e^–^ ORR pathway. Notably, both FePNPC and CoPNPC show higher 2e^–^ ORR activity than their phenyl-substituted counterparts FePhPC and CoPhPC, suggesting that pyrazine unit functionalization is an effective strategy for promoting the 2e^–^ ORR across these molecular catalysts. However, the H_2_O_2_ selectivity of FePNPC and CoPNPC is consistently and significantly lower than that achieved by NiPNPC across the entire potential range. This comparative study clearly establishes NiPNPC as the optimal catalyst within this series for highly selective H_2_O_2_ production via 2e^–^ ORR. The superior performance of NiPNPC thus provides a suitable platform for investigating the mechanistic origin of the high selectivity.

We further employed in situ attenuated total reflectance surface-enhanced infrared absorption spectroscopy (ATR-SEIRAS) and in situ Raman spectroscopy to monitor the formation and evolution of reaction intermediates in real time. As shown in Figures S17–S18, NiPNPC and NiPhPC were compared.^30,49^ The superoxide (O_2_^•–^) signal (1110 cm^–1^) is much weaker for NiPNPC compared to NiPhPC throughout the potential range, underscoring the faster turnover and consumption of O_2_^•–^ on the former catalyst.^64,69^ In 0.1 M KOD, the O_2_^•–^ signal for NiPNPC is stronger than in 0.1 M KOH, suggesting hydrogenation with proton transfer plays a more important role in the H_2_O_2_ production. In situ Raman studies reinforce these observations. Based on the benchmark study by Dong et al.,^70^ which identified *OOH (∼732 cm^–1^), *OH (∼1080 cm^–1^), and O_2_^•–^ (∼1150 cm^–1^) using D_2_O and ^18^O_2_ isotope experiments, we assign our observed bands accordingly. Under an Ar atmosphere (Figure 4b), only the characteristic D and G bands of the CNT support and molecular catalyst are observed. Upon introducing O_2_ and applying a cathodic potential, new peaks emerge simultaneously (Figure 4a). The peak at about 740 cm^–1^ is assigned to the *OOH intermediate, the band at about 1087 cm^–1^ is assigned to the *OH species, and the peak at about 1147 cm^–1^ is attributed to the O–O stretching vibration of adsorbed O_2_^•–^.^70^ This assignment is further supported by our D_2_O isotope experiments (Figure 4c, 0.1 M KOD vs 0.1 M KOH), where the ∼740 cm^–1^ and ∼1087 cm^–1^ bands exhibited slight red shifts (to ∼724 and ∼1080 cm^–1^, respectively), confirming the presence of exchangeable protons, while the ∼1147 cm^–1^ band remained unchanged, consistent with a proton-free O_2_^•–^ species.^70^

**Figure 4:**
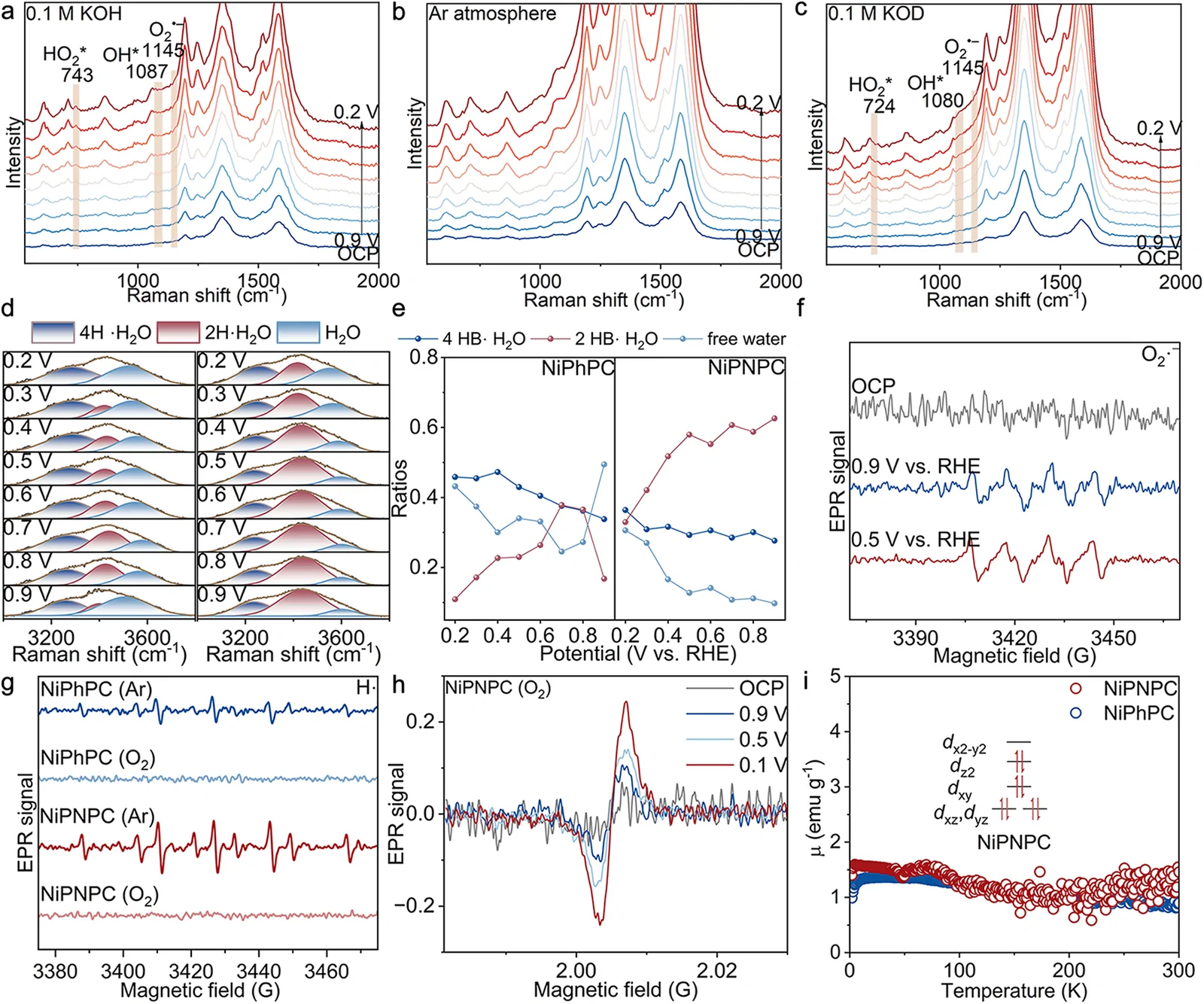
In situ Raman spectra of NiPNPC under (a) O_2_, (b) Ar atmosphere, and (c) 0.1 M KOD. (d) In situ Raman spectra of the O–H stretching vibrational mode of NiPNPC and NiPhPC. (e) Calculated ratio of the interfacial water. (f) The EPR signals of O_2_^•–^ radicals under different potentials. (g) The EPR signals of H radicals for NiPhPC and NiPNPC under Ar and O_2_ atmospheres. (h) The EPR signals of the NiPNPC electrodes under different potentials. (i) The measurement of effective magnetic moments for NiPNPC and NiPhPC.

To probe the reaction pathway and identify key intermediates, we employed in situ electron spin resonance (ESR) spectroscopy using 5,5-dimethyl-1-pyrroline *N*-oxide (DMPO) as a spin-trap agent for NiPNPC under operating potentials.^31,71,72^ As shown in Figure 4f, characteristic signals corresponding to superoxide radicals (O_2_^•–^) are detected with negative potentials,^73,74^ which aligns with the in situ ATR-SEIRAS and in situ Raman results. Considering that efficient hydrogenation also plays a key role in the formation of H_2_O_2_, the characteristic hydrogen radical was further examined. As shown in Figure 4g, NiPNPC exhibits a stronger hydrogen radical signal than that of NiPhPC under an Ar atmosphere. Indicating NiPNPC is superior to NiPhPC for hydrogen radical generation. However, such signals can be consumed under an O_2_ atmosphere, suggesting the existence of hydrogenation and consumption of hydrogen radicals to produce H_2_O_2_ during the reaction.

The observation of a stronger active hydrogen signal for NiPNPC than for NiPhPC prompted us to investigate the structure of interfacial water, as the structure of interfacial water, which mediates proton delivery to the active site, is crucial for the hydrogenation steps. To probe the interfacial water structure, we performed in situ Raman spectroscopy in the O–H stretching region (around 3000–3800 cm^–1^) (Figures 4d,4e, and S21–S28).^52,75,76^ The spectra were deconvoluted into three components, the free water (ca. 3600 cm^–1^), moderately H-bonded water (2HB·H_2_O, ca. 3450 cm^–1^), and strongly H-bonded water (4HB·H_2_O, ca. 3200 cm^–1^), following established literature protocols.^49,51,77^ As shown in Figure 4e, NiPNPC exhibits the highest proportion of 2HB·H_2_O and the lowest proportion of 4HB·H_2_O among all catalysts, indicating that pyrazine functionalization reorganizes the interfacial water network into a more dynamic hydrogen-bonding configuration that facilitates proton transfer.^15,25,27^ Control experiments (Figures S21–S28) reveal that CNT alone also exhibits a relatively high 2HB·H_2_O population, attributed to oxygen-containing functional groups on the CNT surface that attract water. However, CNT lacks Ni sites for O_2_ activation and shows poor H_2_O_2_ selectivity, confirming that water reorganization alone is insufficient for selective 2e^–^ ORR. NiPhPC shows the highest 4HB·H_2_O population, reflecting a rigid water network that impedes proton delivery, consistent with its lower H_2_O_2_ selectivity. ZnPNPC (which contains the same pyrazine units as NiPNPC but d10 Zn with no spin crossover) shows increased 2HB·H_2_O compared to NiPhPC, confirming the hydrogen-bonding role of pyrazine. However, ZnPNPC exhibits poor H_2_O_2_ selectivity, further confirming that the Ni-centered spin crossover is essential for high catalytic activity. The observed higher 2HB·H_2_O population, the enhanced hydrogen radical signal, and the observed KIE (>1) all correlate with the accelerated proton transfer enabled by the reorganized interfacial water network and higher H_2_O_2_ selectivity for NiPNPC than NiPhPC.

After examining the surface and interfacial behavior of the catalysts, we turned to probe the metal sites during reactions. As exhibited in Figures 4h, S32 and S33, the pristine NiPNPC shows a negligible EPR signal at OCP due to its low-spin state. Upon cathodic polarization, however, an obvious potential-dependent response emerges, irrespective of the atmosphere (Ar or O_2_), indicating that unpaired electrons respond to the applied potential. Control experiments (Figure S32) with CNT, NiPhPC, NiNPC, metal-free ligand, and ZnPNPC confirm that this potential-dependent EPR signal originates from the Ni center, while ligand-centered radicals and CNT defects do not contribute. Magnetic moment tests further exclude a static high-spin state for both NiPNPC and NiPhPC (calculated to be ca. 2.83 for a high-spin state). The observation that NiPNPC shows almost no signal at OCP but a clear signal under cathodic polarization, despite a low-spin ground state, provides direct evidence for a potential-driven spin crossover at the Ni site. To verify this dynamic spin-state transition, we performed operando X-ray emission spectroscopy (XES) measurements (Figures 5a,b, and S34).^78^ XES is highly sensitive to spin state. The evolution of the Kβ′ emission line under applied potential provides a direct spectral characteristic of the Ni spin state. Operando XES analysis, calibrated against NiPC (low-spin) and NiO (high-spin) references, reveals a potential-driven transition of the Ni center from low-spin to high-spin under cathodic polarization. The high-spin fraction shows a clear potential-dependent increase, reaching approximately 50% at 0.3 V vs RHE (semi-quantitative estimate based on linear interpolation between the OCP and 0 V spectra). The spectral features of NiPNPC shift with applied potential under both Ar and O_2_ atmospheres (Figure S34), confirming that the spin transition is primarily potential-driven. The spin crossover is attributed to the introduction of pyrazine units to the macrocyclic ligand, which lowers the energy barrier for spin crossover, as supported by DFT calculations (Figure S40). This spin state change aligns with the EPR signals of the NiPNPC electrode under applied potentials. In contrast, the change in the XES spectra for NiPhPC was much smaller than that of NiPNPC under different applied potentials (Figure S34), confirming a relatively weak spin state change when combined with the magnetic measurement. These results indicate that the high-spin Ni species critically contributes to the 2e^–^ ORR on NiPNPC.

**Figure 5:**
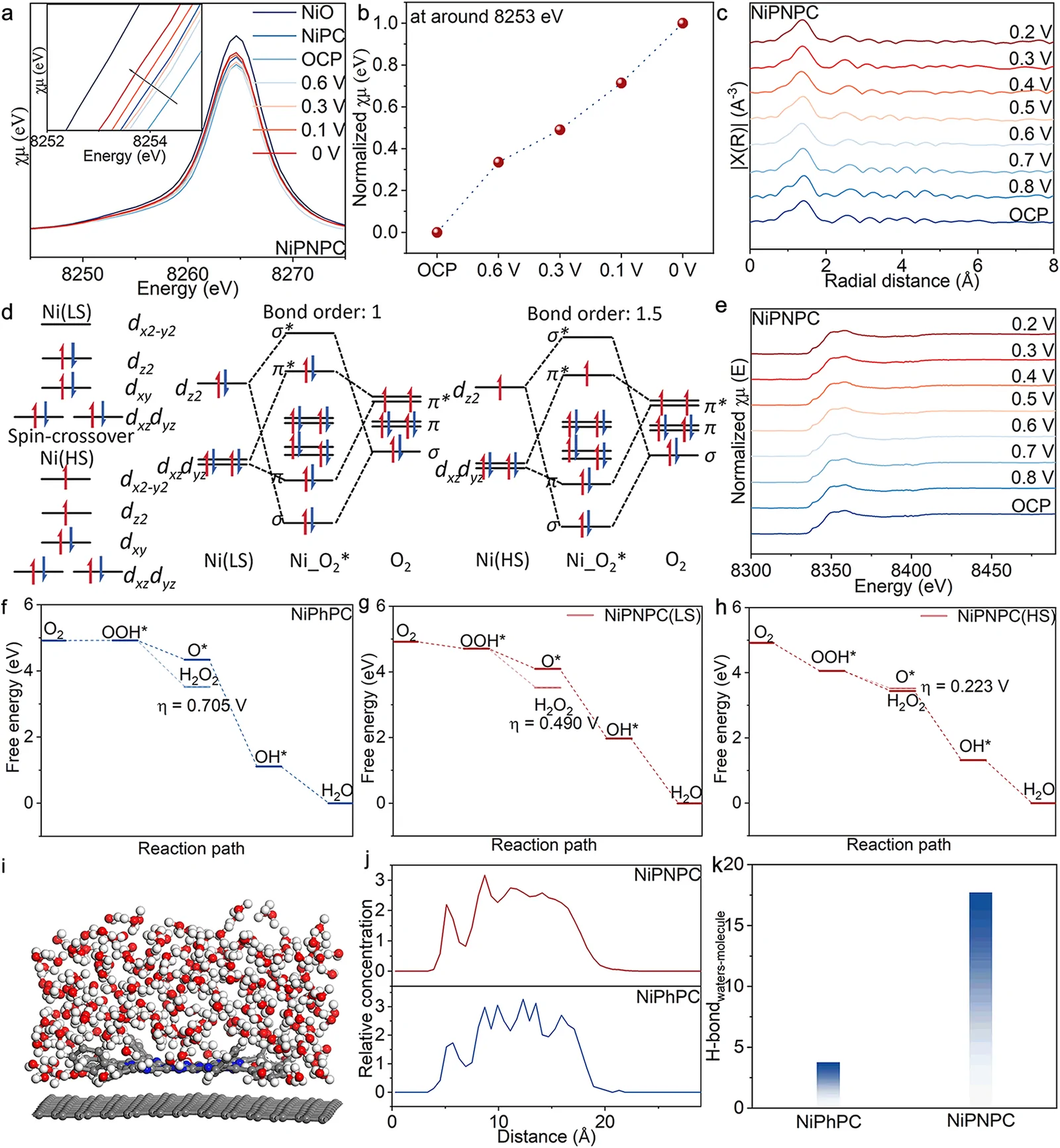
(a) Operando Ni Kβ’ XES spectra for NiPNPC. (b) Normalized intensity comparison of Kβ’ signal at around 8253 eV. (c) Operando Ni K-edge XANES of NiPNPC under O_2_ atmosphere. (d) The orbital interactions between low-spin (LS) and high-spin (HS) Ni sites and the triplet O_2_ molecule for NiPNPC. (e) Operando Fourier-transformed k-space of NiPNPC under O_2_ atmosphere. (f–h) Calculated 2e^–^ and 4e^–^ ORR reaction pathway. (i) Molecular dynamics simulation results of the interfacial structure for NiPNPC. (j) Relative concentration of water along the *z*-axis of NiPNPC and NiPhPC. (k) Calculated H-bond between water and molecules.

Operando X-ray absorption near-edge structure (XANES) spectroscopy tests were performed for NiPNPC to obtain further insights into the oxidation state and coordination environment of the Ni atom during the reaction. Under an O_2_ atmosphere, Figure 5c displays the normalized Ni K-edge XANES spectra. The operando XANES spectra reveal that the oxidation state of Ni under all applied potentials are close to +2. The coordination environment was also investigated from the characteristic 1s–4p peak, where a clear peak at around 8338 eV, indicative of four-coordinate Ni centers, is observed under different potentials. Operando Fourier-transform extended X-ray absorption fine structure spectra (Figure 5e) with prominent first-shell peaks and a fitted coordination number of approximately 4 (Figure S35) are observed with no Ni–Ni bond detected, confirming that Ni remains in a 4-coordinated, atomically dispersed state during reactions. The different behaviors of high-spin and low-spin state Ni can be further understood through orbital analysis. The orbital interactions between Ni sites with different spin states and the triplet O_2_ molecule were analyzed. As shown in Figure 5d, the oxygen molecule is more readily captured by high-spin Ni than by low-spin Ni because of a higher bond order resulting from the spin-orbital interactions between high-spin Ni and O_2_, thus ensuring effective activation of the triplet O_2_ molecule. It should be noted that, after O_2_ end-on adsorption, NiPNPC may decrease from a D4h to *C*_4*v*_ symmetry. Under this symmetry, the d_*z* ^2^_ orbital with A_1_ irreducible representation (irrep) is symmetry-matched to the O_2_ σ-type orbital (A_1_), while the d*_xz_*/d*_yz_* orbitals (E) match the O_2_ π* orbitals (E). The d_*x* ^2^–*y* ^2^_ (B_1_) and d*_xy_* (B_2_) orbitals have zero overlap with O_2_σ and π* when considering the point group symmetry, although they indirectly influence the overall electron-donating properties of the metal. The present qualitative analysis provides a simplified model of the bond order and orbital interactions.

Having established the dynamic behaviors of both the interfacial water network and the Ni spin state, we next examined their interplay across the potential range. Notably, EPR measurements reveal the presence of superoxide radicals across a wide potential range, indicating that O_2_ activation occurs continuously throughout the operating window. Meanwhile, operando XES shows that the spin-state signal evolves progressively with applied potential, confirming that the Ni center maintains its spin-crossover capability. Concurrently, the population of weakly H-bonded water (2HB·H_2_O), which is established at higher potentials, is consumed as the potential shifts negatively, consistent with its role in supplying protons for hydrogenation. These observations support the conclusion that spin crossover and hydrogen-bond network engineering operate in a concerted manner. Triplet O_2_ is adsorbed and activated on the in situ generated high-spin Ni site, leading to electron transfer and the formation of a superoxide intermediate (as verified by DFT, Figures S40–S41). Simultaneously, the pyrazine-reorganized hydrogen-bonding network facilitates proton delivery and promotes the protonation of the superoxide intermediate to form the key *OOH intermediate, and the subsequent hydrogenation step then yields H_2_O_2_. This dynamic picture, in which spin-crossover enables O_2_ activation and an engineered hydrogen-bonding network governs selective protonation, provides a fundamental explanation for the superior performance of NiPNPC.

Density functional theory (DFT) calculations were performed to gain deeper insights into the mechanism. Geometry optimization reveals a slightly longer average Ni–N bond length in the NiPNPC macrocycle (1.920 Å) compared to NiPhPC (1.911 Å). Crystal orbital Hamiltonian population (COHP) analysis yields integrated COHP values of −1.162 eV for NiPNPC and −1.163 eV for NiPhPC (Figure S42), which are nearly identical. This indicates that the pyrazine substitution induces only a minor electronic perturbation at the Ni center. The primary electronic effect of pyrazine is better captured by the operando XES and spin-crossover energy calculations (Figures S40–S41), which show a significantly reduced energy difference for the low-spin to high-spin transition in NiPNPC. We then computed the free energy profiles for the 2e^–^ ORR pathway (Figures 5f–5h, and S38–S39). For NiPNPC in its low-spin state, the potential-determining step (PDS) for *OOH formation has an energy difference of 0.490 eV. Crucially, when accounting for the in situ generated high-spin state identified by XES, this energy difference drops significantly to 0.223 eV. In contrast, the PDS energy difference for NiPhPC is calculated to be 0.705 eV. This large difference (approximately 0.480 eV) in the activation energy rationalizes the superior H_2_O_2_ selectivity of NiPNPC. Finally, molecular dynamics (MD) simulations were conducted to probe the interfacial structure (Figures 5i–5k and S45). The water molecules around the catalyst show a distinct peak near 5 Å, corresponding to the relative concentration between water and the catalyst. As shown in Figure 5j, the intensity of the peak is substantially higher for NiPNPC than for NiPhPC, demonstrating that the pyrazine N atoms indeed promote the accumulation and reorganization of water at the interface, thereby leading to a changed hydrogen-bonding network at the interface. From the calculation, it is found that NiPNPC could form more hydrogen bonds with water layers compared to that of NiPhPC (Figure 5k), which aligns with our in situ spectroscopic observations observed from Figure 4e. Thereby, the decoration of the pyrazine unit could disrupt the tetrahedral water network of the interface, leading to a weakened rigid 4HB·H_2_O network and enhanced 2HB·H_2_O population. This MD-derived picture is consistent with the Raman spectral deconvolution and is further supported by the enhanced hydrogen radical signal in EPR and the kinetic isotope effect observed in D_2_O electrolyte. The convergence of computational and experimental evidence confirms that pyrazine-induced water reorganization facilitates proton transfer, thereby promoting the selective formation of H_2_O_2_.

Traditional H-cell systems for H_2_O_2_ production usually require electrolytes that contain inorganic salts, raising both production expenses and downstream purification costs. To circumvent this limitation, we used a porous solid electrolyte (PSE) reactor with a design that allows deionized water to replace conventional electrolyte solutions (Figure S47).^79,80^ In this setup, protons formed at the anode travel through a proton exchange membrane (PEM) into the middle porous solid electrolyte layer, while HO_2_^–^ generated at the cathode passes through an anion exchange membrane (AEM) into the same layer, the two species then combine to form pure H_2_O_2_.^81^ We evaluated NiPNPC in this PSE reactor under galvanostatic operation, with current densities ranging from 10 to 200 mA cm^–2^ (Figure 6a–c). Across the entire range, the cell maintained a Faradaic efficiency (FE) for H_2_O_2_ above 80%, reaching a maximum of approximately 95.04% at 10 mA cm^–2^ and remaining around 84.42% at 200 mA cm^–2^. At the highest current density, the production rate reached 3.15 mmol cm^–2^ h^–1^ with a cell voltage below 2.99 V. These results indicate that the high selectivity observed in fundamental RRDE measurements translates effectively to a more practical system. The PSE reactor also showed robust durability with NiPNPC (Figure 6d). At an industrially relevant current density of 100 mA cm^–2^, the Faradaic efficiency remained above 88% for at least 100 h of continuous operation. Over this period, the outlet H_2_O_2_ concentration stayed above 3400 ppm at a flow rate of 0.5 mL min^–1^. Taken together, integrating the NiPNPC cathode catalyst with the PSE reactor enables H_2_O_2_ collected from the water-fed PSE stream with high selectivity and long-term durability, demonstrating the practical potential of our molecular design strategy.

**Figure 6:**
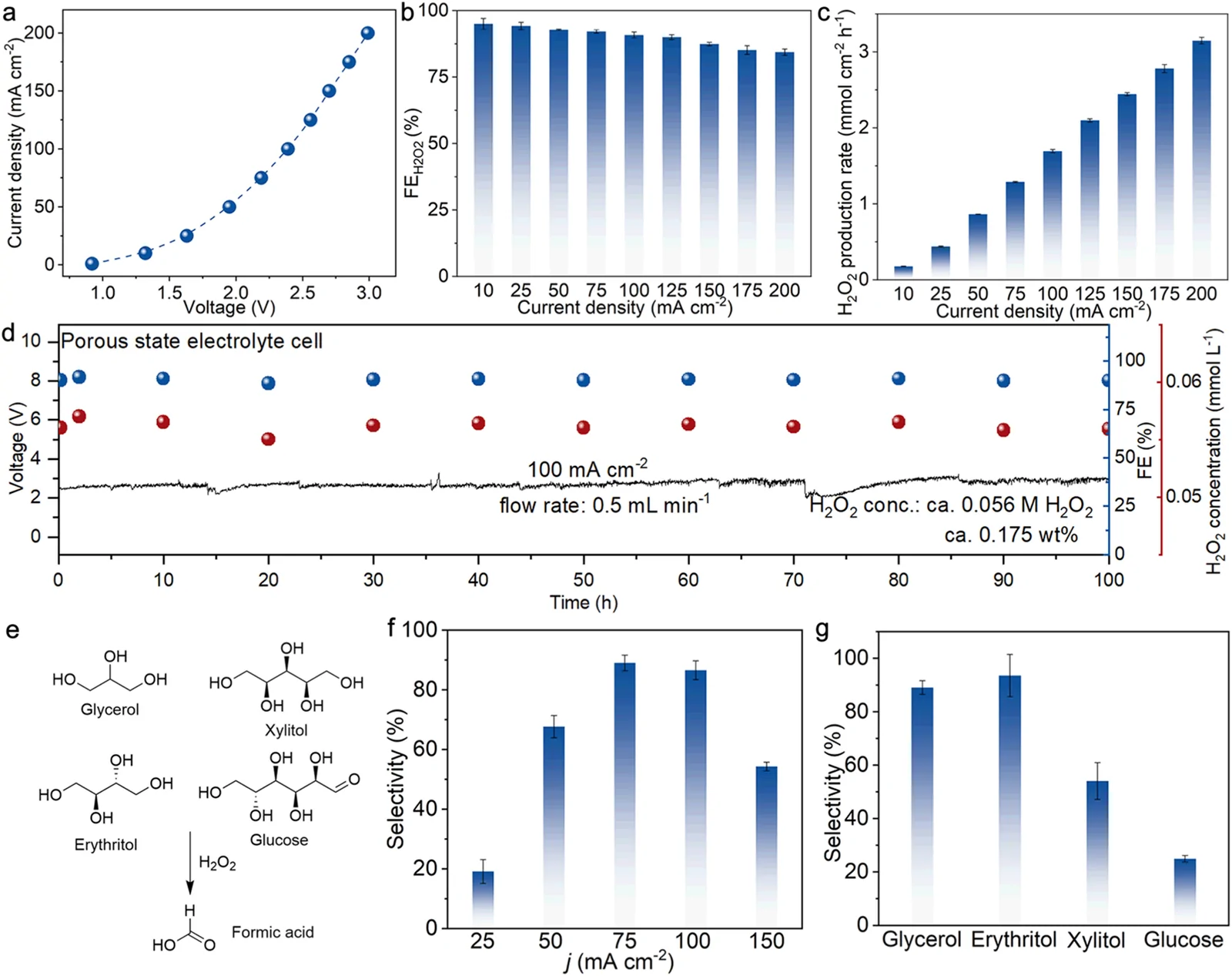
(a) *I*–*V* curve for H_2_O_2_ production of NiPNPC in the porous solid electrolyte reactor. (b) Faradaic efficiency of H_2_O_2_. (c) H_2_O_2_ production rate. (d) Stability test. (e) The molecules used for biomass conversion with H_2_O_2_ to produce formate. (f) The selectivity to yield formate for Glycerol with H_2_O_2_ produced under different current densities. (g) The selectivity comparison of Glycerol, Erythritol, Xylitol, and Glucose to yield formate.

Motivated by the exceptional 2e^–^ ORR selectivity in both RRDE and PSE, we further evaluated the practical potential of NiPNPC by assembling it into a gas diffusion electrode (GDE) and testing it in a flow cell reactor (Figure S48).^80,82–84^ As shown in Figure S49, the H_2_O_2_ production rate increases with applied current density, reaching a high yield rate of 19.31 mol g^–1^ h^–1^ at 200 mA cm^–2^. A long-term stability test at 50 mA cm^–2^ (Figure S50) demonstrates stable operation over 52 h, accumulating H_2_O_2_ at a concentration of 1.71 wt % in the electrolyte. The SEM and XPS data before and after stability tests confirm the good stability of NiPNPC under reaction conditions (Figures S51 and S52). Considering the challenges associated with H_2_O_2_ separation and storage, we further investigated its direct utilization in a tandem electrochemical and chemical process for biomass transformation (Figure 6e).^64,85,86^ Using the in situ generated H_2_O_2_, glycerol was selectively oxidized to formate in the alkaline electrolyte. As shown in Figure 6f, a formate selectivity of approximately 89.08% was achieved at a current density of 75 mA cm^–2^. This tandem strategy was successfully extended to other biomass-derived polyols, including erythritol, xylitol, and glucose (Figure 6g). Among them, erythritol afforded the highest formate selectivity (up to 93.53%), highlighting the versatility of this integrated approach for producing valuable chemicals from renewable sources.

## Conclusions

In summary, we have demonstrated a synergistic molecular design that integrates dynamic spin-state regulation with interfacial hydrogen-bond engineering to achieve highly selective H_2_O_2_ electrosynthesis. In the pyrazine-functionalized NiPC catalyst (NiPNPC), O_2_ adsorption is triggered by a potential-driven spin-crossover at the Ni center from low-spin to high-spin, which resolves the spin mismatch and facilitates the formation of a superoxide intermediate. Concurrently, the pyrazine units trigger the assembly of an optimized hydrogen-bonding network at the catalyst-electrolyte interface, ensuring efficient proton delivery for the formation of *OOH intermediate and the selective formation of H_2_O_2_ (up to 97.15%). Advanced in situ techniques, accompanied by computational techniques, explained the spin-state transformation, intermediate pathway, and the optimized *OOH. This work shifts the design paradigm for molecular electrocatalysts from static electronic and geometric tuning toward the active co-regulation of dynamic spin states and the local reaction microenvironment. The strategy of combining spin-crossover motifs with functional units that tailor interfacial networks represents a broadly applicable blueprint for the rational design of advanced catalysts across diverse proton-coupled electron transfer processes

## Supplementary Material



## Notes

Detailed synthesis, characterization and DFT calculation methods; HRMS, UV–vis, EXAFS fitting data, additional electrocatalytic data, in situ ATR-SEIRAS, in situ Raman, EPR, Operando XES, Operando XAFS, additional DFT calculations, PSE and flow cell setup, additional flow cell data (PDF)
