# Supplementary material for: Potential Driven Spin-Crossover Synergized with Hydrogen-Bond Engineering in a Molecular Catalyst for Selective H2O2 Electrosynthesis
Source: J Am Chem Soc. 2026 Sep 14;148(38):41673–85. doi: 10.1021/jacs.6c16940 (PMC13632549; doi:10.1021/jacs.6c16940)
Supplement: Supplementary file 1 [file ja6c16940_si_001.pdf]

# Potential Driven Spin-Crossover Synergized with Hydrogen-Bond Engineering in a Molecular Catalyst for Selective $H_2O_2$ Electrosynthesis


Libo Sun,[1] Yanjie Zhai,[1] Zhiying Wu,[1] Dongxue Yu,[1] Zhiqiang Liang,[2] Zhen-An Qiao,[2] Hua Zhang,[1,3,4,5] and Xin Wang*[1,5]

[1]Department of Chemistry, City University of Hong Kong, Kowloon, 999077, Hong Kong SAR, P. R. China.

[2]State Key Lab of Inorganic Synthesis and Preparative Chemistry, College of Chemistry, Jilin University, Changchun 130012, P. R. China.

[3]Hong Kong Institute for Clean Energy, City University of Hong Kong, Kowloon, 999077, Hong Kong SAR, P. R. China.

[4]Shenzhen Research Institute, City University of Hong Kong, Shenzhen, 518057, China.

[5]Hong Kong Branch of National Precious Metals Material Engineering Research Center, City University of Hong Kong, Kowloon, 999077, Hong Kong SAR, P. R. China

*Corresponding author: E-mail: wang.xin@cityu.edu.hk

**Experimental section.**

**Chemicals.**

Nafion perfluorinated resin solution (5 wt.% in lower aliphatic alcohols and water, containing 15 – 20% water), nickel acetate tetrahydrate (98%), benzil (98%), 40% aq glyoxal solution, diaminomaleonitrile (96%), cerium sulfate (98%), and quinoline (>98.0%) were purchased from Dieckmann. Dowex 50WX8 (hydrogen form) was bought from Sigma-Aldrich. Multi-walled carbon nanotubes (CNT, 5 – 15 nm, >99wt%) were purchased from Chengdu Organic Chemicals Co., Ltd. KOH (99.95%) was purchased from Macklin Inc. Sigacet 28BC and 39 BB gas diffusion layer, PIPON-60, $IrO_2$ powder, Nafion 212, and Nafion 117 membrane were purchased from Fuel Cell Store. Carbon cloth was bought from Sheng Ernuo., Ltd. Solvents were purchased from CityU Mini Store.

**Synthesis of nickel phthalocyanine derivatives.**

**Synthesis of NiNPC.** The synthesis of pyrazine-2,3-dicarbonitrile was according to the literature,[1] where diaminomaleonitrile (2 g, 18.5 mmol) and 40% aq glyoxal solution (3 mL, ca. 20 mmol) were mixed and added in boiling ethanol (10 mL). After adding concentrated HCl (0.3 mL), the system was refluxed for 30 min. Subsequently, the system was cooled to room temperature, and the crystalline product was precipitated. After filtration, the mixture was collected by centrifugation and washed with cold ethanol and hexane to obtain the white needle product: $^1H$ NMR ($CDCl_3$, 300 MHz) δ: 8.94 (s, 2H). After drying, the product (300 mg) was mixed with nickel acetate tetrahydrate (100 mg) in quinoline (4 mL), then the system was reacted at 200 $^oC$ under an Ar atmosphere for 24 hours. After cooling to room temperature, the product was precipitated from diethyl ether, and the green-colored product obtained was washed repeatedly with a 1:1 mixture of methanol/diethyl ether three times. Then it dried at 60 $^oC$ overnight to obtain the product. HRMS: $M^+$, 578.0491, Analysis: $C_{24}H_8N_{16}Ni$, 578.0471.

**Synthesis of NiPNPC.** The synthesis of 5,6-diphenylpyrazine-2,3-dicarbonitrile was according to the literature,[2] where benzil (2.1 g, 0.01 mol), diaminomaleonitrile (2.16 g, 0.02 mol) were mixed with $H_2O$/ethanol/HOAC (24/36/2 mL) under an inert gas atmosphere. After refluxing at 85 $^oC$ for 2h, a light yellow color product can be precipitated after the reaction system has cooled down to room temperature. The product was filtered and washed with cooled ethanol three times and dried: $^1H$ NMR ($CDCl_3$, 300 MHz) δ: 7.50 (m, 4H), 7.47 (d, $J$ = 1.5 Hz, 2H), 7.42 (dd, $J1$ = 5.7 Hz, $J2$ = 6.9 Hz, 4H). The synthesis of NiPNPC was the same as that of NiNPC, the only difference is that 5,6-diphenylpyrazine-2,3-dicarbonitrile (300 mg) was used instead of pyrazine-2,3-dicarbonitrile. The synthesis of NiPNPC can also use $H_2$PNPC as precursor, where the synthesis of $H_2$PNPC can be employed using below method. 10 mL of n-pentanol was added into a 50 mL flask protected with Ar atmosphere, and Li pellet (1g) was added to the system (caution: this step is extremely dangerous and may cause fire!). Then the system was heated to around 60 $^oC$, after Li pellet reacted, diaminomaleonitrile (1.13 g, 0.01 mol) was

added and allow the system to react at around 100 °C overnight. After quench the system with ethanol and DI water, n-pentanol was removed by evaporation, and green powders may form in the solvent. The powders can be precipitated, filtered, and washed with ethanol and water for three times to obtain the green powders, which can be used as precursors to prepare NiPNPC. HRMS: $M+NH_4^+$, 1148.9107, Analysis: $C_{72}H_{42}N_{16}$, 1130.3778. HRMS: $M^+$, 1186.3025, Analysis: $C_{72}H_{40}N_{16}Ni$, 1186.2975.

**Synthesis of NiPhPC.** NiPhPC was used directly as reported in our previous research.[3]

**Electrochemical measurement.**

**RRDE tests.** CNT was bought from Times Nano Ltd. and used directly. The immobilization of NiPC derivatives onto CNT to obtain heterogeneous catalysts can be found below. Briefly, 100 mg of CNT were added to 40 mL DMF and sonicated for one hour. Meanwhile, a calculated amount of NiPC derivatives was added to another DMF solution (10 mL) and sonicated for 30 min. Then they were mixed and further sonicated for at least one hour. Then the resulting mixture was stirred overnight to obtain well-mixed solution. Afterwards, 60 mL of diethyl ether was added to the system and centrifuged. The materials were further washed with diethyl ether for three times and dried in the fume hood overnight, then they were dried at 80 °C in a vacuum oven to obtain the catalysts. The electrochemical $H_2O_2$ production was performed with a rotating ring-disk electrode (RRDE) system from Metrohm autolab. A saturated calomel electrode (SCE) and a graphite rod were used as the reference and counter electrode, respectively. To prepare the ink, 10 mg composite was dispersed in 1.95 mL isopropanol and 0.05 mL 5 wt.% Nafion. Then the mixture was sonicated for at least 0.5 h to obtain a homogeneous ink. 5 µL of the above-prepared ink was drop-casted on the glassy carbon disk. The measurement was conducted in 0.1 mol KOH. The rotating speed and the scan rate were controlled to be 1600 rpm and 5 mV $s^{-1}$, respectively. The potential applied for obtaining the ring current of the RRDE during $H_2O_2$ production is 1.2 V versus RHE. The $H_2O_2$ selectivity and the electron transfer number on the RRDE of the electrocatalysts were calculated based on below equation:

$$H_2O_2\ (\%)=200 \times I_R/N(I_R/N+I_D) \qquad (1)$$

$$n=4\times I_R/N(I_R/N+I_D) \qquad (2)$$

where $I_R$ is the ring current, $I_D$ is the disk current, and N is the collection efficiency of RRDE.

**Flow cell test.** 4 mg catalyst was added into 7.98 mL isopropanol, 0.02 mL perfluorinated Nafion resin solution (5%), and sonicated to prepare the catalyst ink. The catalyst ink was air-brushed onto the gas diffusion layer (Sigracet 28BC) with a loading of 0.2 mg $cm^{-2}$, and the reaction window was controlled to be 1.0 $cm^{-2}$. The platinum mesh and a saturated Ag/AgCl electrode served as the counter and reference electrode in 1.0 M KOH, respectively. Anode and cathode chambers were separated by Nafion 212 membrane. The experiments were carried out with a flow rate of 25 mL $min^{-1}$ for $O_2$ and a flow

rate of 25 mL $min^{-1}$ for electrolyte. The generated $H_2O_2$ is determined by the cerium sulfate titration method.

The concentration was measured by the cerium sulfate titration method according to the following equation:

$$2Ce^{4+} + H_2O_2 = 2Ce^{3+} + 2H^{+} + O_2 \qquad (3)$$

The yellow $Ce^{4+}$ would convert into colorless $Ce^{3+}$, and the change of color was determined by UV-vis to quantify the amount of consumed $Ce^{4+}$, so as to obtain the concentration of $H_2O_2$.

**FE calculation.** The FE for $H_2O_2$ production was calculated using the following equation:

$$FE\ (\%) = (n \times F \times C_{H2O2} \times V) / Q \times 100\% \qquad (4)$$

where n = 2 (number of electrons transferred for $H_2O_2$ generation), F = 96485 C $mol^{-1}$ (Faraday constant), $C_{H2O2}$ = concentration of $H_2O_2$ determined by UV-vis absorbance of cerium sulfate, V = volume of electrolyte, and Q is total charge passed. This equation has been added to SI.

**Porous solid electrolyte (PSE) reactor measurements.** $H_2O_2$ was collected from the water-fed PSE stream reactor. This reactor features an anion exchange membrane (AEM; PIPON-60, Fuel Cell Store) and a proton exchange membrane (PEM; Nafion 117, Fuel Cell Store), with a styrene-divinylbenzene sulfonated copolymer PSE layer (Dowex 50WX8, hydrogen form, Sigma-Aldrich) positioned between the two membranes. Around 1.0 mg $cm^{-2}$ NiPNPC catalyst and $IrO_2$ catalyst (Fuel Cell Store) were loaded on Sigracet 39 BB GDL electrode and Ti felt as the cathode and anode, respectively. The cathode side was supplied with 40 sccm of $O_2$ gas feed rate. The anode side was circulated with 0.1 M $H_2SO_4$ aqueous solution at 5 mL $min^{-1}$, while the middle PSE chamber was pumped with pure $H_2O$ solution at 0.5 mL $min^{-1}$. Cell potential was recorded in a two-electrode configuration without iR compensation. The generated $H_2O_2$ concentration Ctitration was quantified by $Ce^{4+}$ titration method. For stability test, carbon cloth was used as the substrate.

**In situ ATR-SEIRAS test.** The in situ ATR-SEIRAS experiments were performed on a Bruker INVENIO FT-IR Spectrometer using an MCT detector. The PIKE VeeMax III electrochemical device was employed for the tests, and the Si prism was sputtered with a 50 nm Au layer. The reflection angle of the PIKE VeeMax III was set to an angle of 60 $^{o}$C. The as-prepared catalyst ink was drop-casted onto a Si prism (0.1 mL), and the in-situ signals with applied potentials were collected in 0.1 M KOH saturated with $O_2$ atmosphere.

**In situ Raman test.** The in situ Raman measurements were carried out on a Horiba XploRA Plus Spectrometer. The shell-isolated nanoparticle-enhanced Raman (SHINERS) was used. The catalysts were drop-casted onto carbon paper as the working electrode. Platinum wire and a saturated Ag/AgCl electrode were used as counter and reference electrode, respectively. The cell bought from Shanghai

Chuxi Ltd. was employed for experiments. The signals in 0.1 M KOH with saturated Ar and $O_2$ atmosphere under applied potentials were collected with 532 nm light source.

**Operando XAS and XES tests.** The operando XANES and XES tests were taken on a Rapidxafs instrument from Anhui Absorption Spectroscopy Analysis Instrument Co. Ltd.[4] The silica (Si551) was selected for recording signals, and 20 kV, 20 mA was used for EXAS tests, 25 kV, 3 mA was used for XES tests. For both tests, 0.1 M KOH with a saturated $O_2$ or Ar atmosphere was used during the collection of data.

**Measurement of effective magnetic moments.** The magnetic moment data were obtained using a Quantum Design PPMS-9 magnetometer on bulk powder samples of NiPNPC and NiPhPC at a temperature control range of 2–300 K under an applied field of 0.1 T. The effective magnetic moment ($\mu_{eff}$) was calculated as $\mu_{eff}= 2.828(\chi_m T)^{1/2}$ in Supporting Information. (T: temperature. $\chi_m$: molar magnetic susceptibility).

**Computational details.**

The molecular structures and related electronic structure information of these nickel phthalocyanine derivatives were calculated using Gaussian software.[5] B3LYP hybrid functional was used for geometry optimization, the pseudopotential LANL2DZ basis set was considered for the nickel atom, and the 6-311+G(d) basis set was considered for the other elements in the models, the Tomasi's polarized continuum model (PCM) was employed for optimization in the aqueous phase.[6-9] The Van der Waals interaction was corrected using Grimme's DFT-D3 with Becke-Jonson damping method.[10, 11] The electronic structure information was analyzed with the assistance of MULTIWFN 3.7 and VMD software.[12, 13]

For calculating the Gibbs free energy and electronic structure analysis during reactions, the density functional theory (DFT) was applied using the Vienna Ab Initio Simulation Package (VASP) code with consideration of spin-polarization.[14] All the calculations were based on the generalized gradient approximation (GGA) with the description of atomic potential by the projector augmented wave (PAW) pseudo-potential, and the exchange-correlation potentials were modeled using Perdew–Burke–Ernzerhof (PBE) functional.[14, 15] Grimme's DFT-D3 with Becke-Jonson damping method was applied for correcting the Van der Waals' interactions with use of implicit solvent model. To avoid the possible interaction between adjacent atoms in the unit, a vacuum layer thickness of over 20 Å was set along the z-axis. The convergence threshold force and energy during optimization were set to be 0.02 eV $Å^{-1}$ and $10^{-5}$ eV, and the plane-wave cut-off energy was set to be 450 eV. The k-point grids of 1 × 1 × 1 were used for the models. The Gibbs free energy difference ($\Delta G$) for each elementary reaction step was calculated using the following formula, based on the computational hydrogen electrode (CHE) model.[16]

$$\Delta G=\Delta E+\Delta E_{ZPE}-T\Delta S \qquad (5)$$

where $E$, $E_{ZPE}$, $T$, and $S$ are the total electron energy, zero-point energy, temperature, and entropy, respectively. The data were treated with the assistance of vaspkit.1.02 software.[17]

For molecular dynamics simulations, the Forcite module was employed. The simulation process adopts regular integration (NVT), with a time step of 1.0 fs and a total simulation duration of 2000 ps. Temperature and pressure were controlled by the North-Hoover thermostat. The water layers were constructed with a density of approximately 1.0 g $mL^{-1}$. The hydrogen bond between NiPNPC/NiPhPC and water was defined with geometric criteria: O···H distance $< 3.1$ Å and O–H···O angle $> 90°$. The values reported in Figure 5k are the frame averaged molecule–water hydrogen bond counts.

**Characterizations.**

The UV–vis absorption measurements were performed on a Shimadzu 1900i spectrophotometer. X-ray photoelectron spectroscopy (XPS) data were taken on a Thermo Scientific K-Alpha instrument. The Raman spectra data were measured on a Horiba Raman microscope. Transmission electron microscopy and EDS-mapping images were obtained on a JEOL JEM 2100PLUS instrument. High-resolution mass (HRMS) data were collected on a Thermo Scientific Q Exactive with ESI scanning mode. The ATR-SEIRAS spectra were measured using a Bruker Invenio spectrometer. The ICP-MS data were taken on an Agilent 7700 (MS). The XAS and XES data were taken on a RapidXAFS 2 M (Anhui Absorption Spectroscopy Analysis Instrument Co., Ltd.) by transmission mode at 20 kV and 20 mA, and 25 kV and 3 mA. The aberration-corrected high-angle annular dark-field scanning transmission electron microscopy images were obtained on the FEI Themis Z instrument. EPR data were obtained on a Bruker EMXnano instrument. The magnetic behaviour was taken on a LakeShore 8604 instrument.

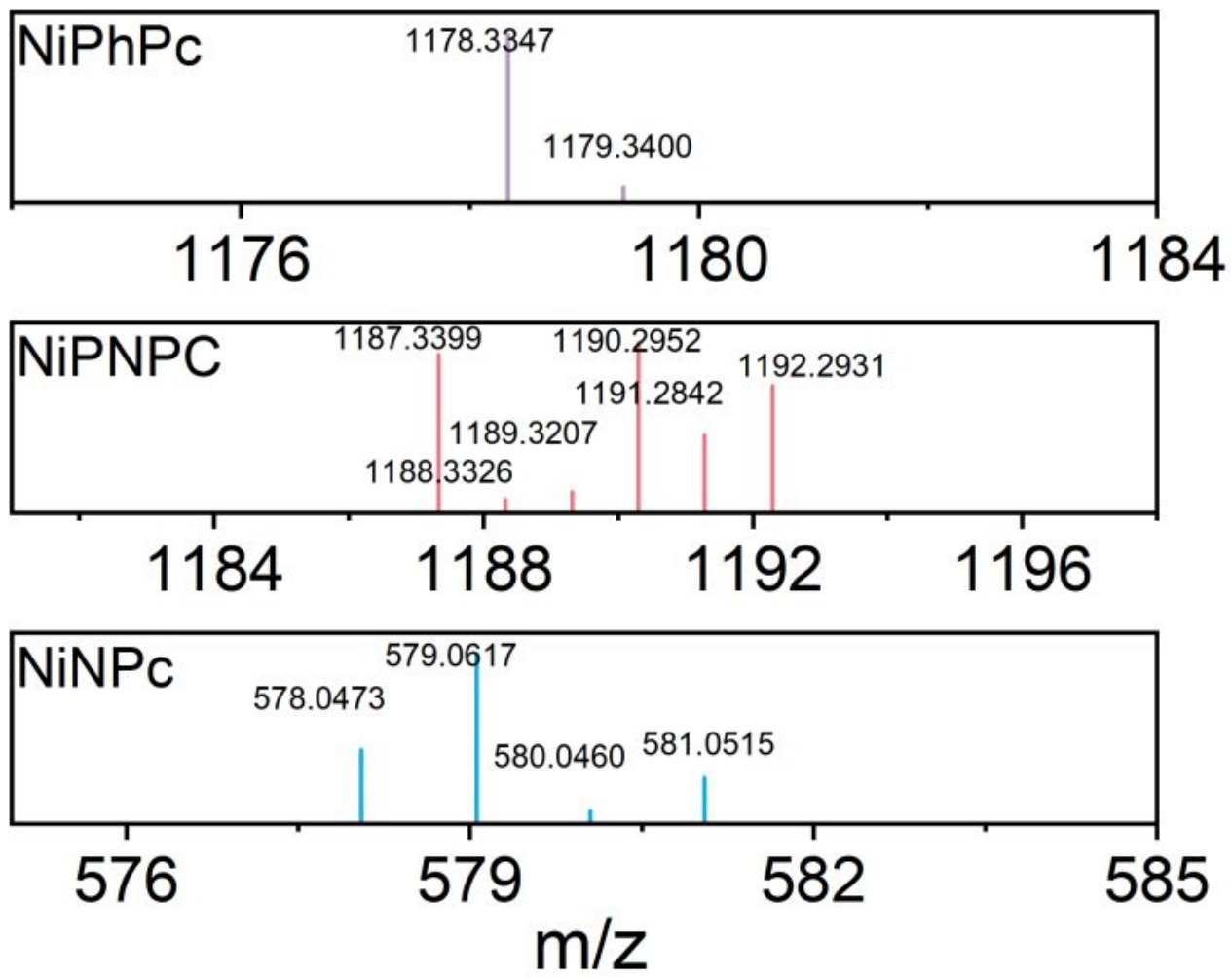


Figure S1. Mass of NiNPc, NiPNPc, and NiPhPc.

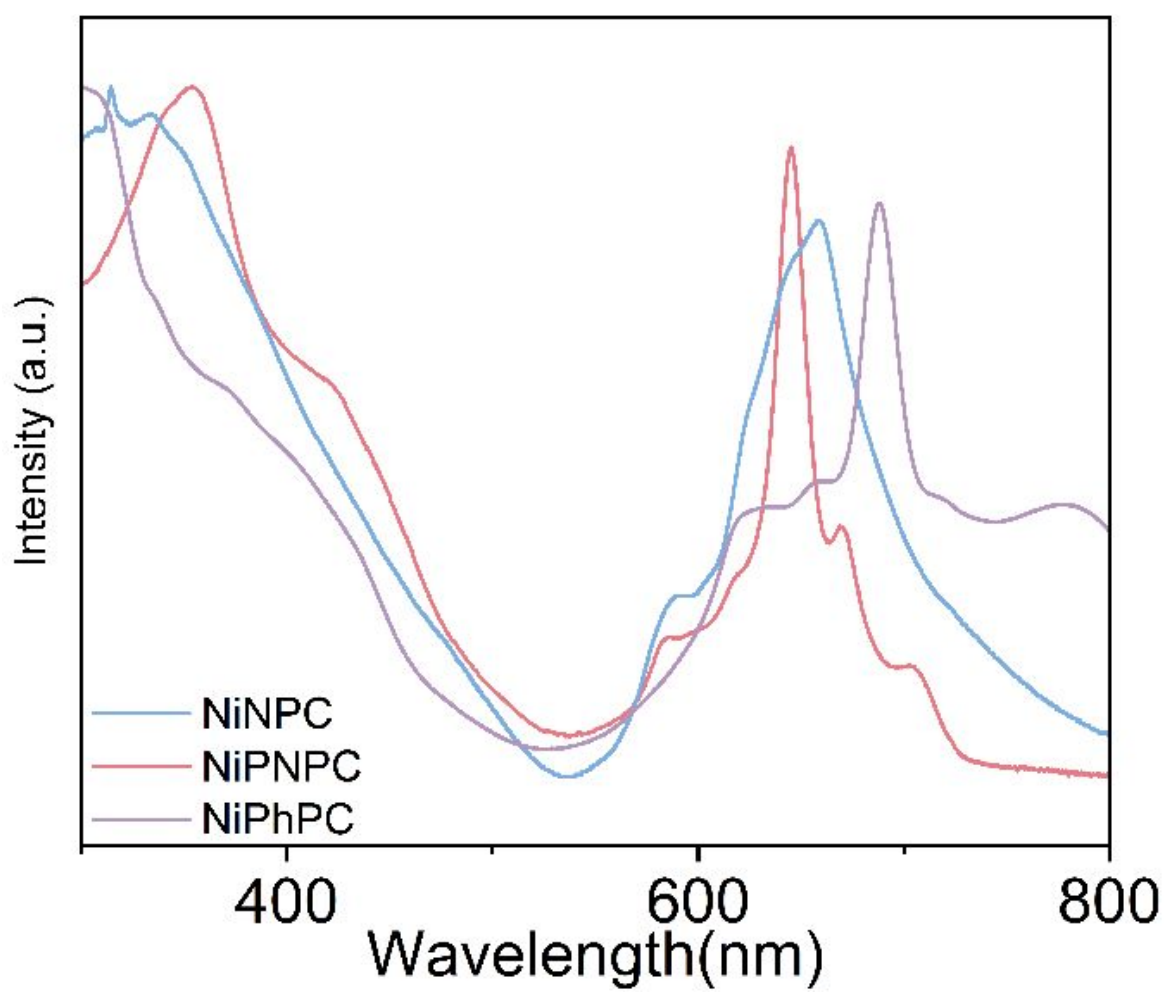


Figure S2. UV-vis data of NiNPc, NiPNPc, and NiPhPc in $CHCl_3$.

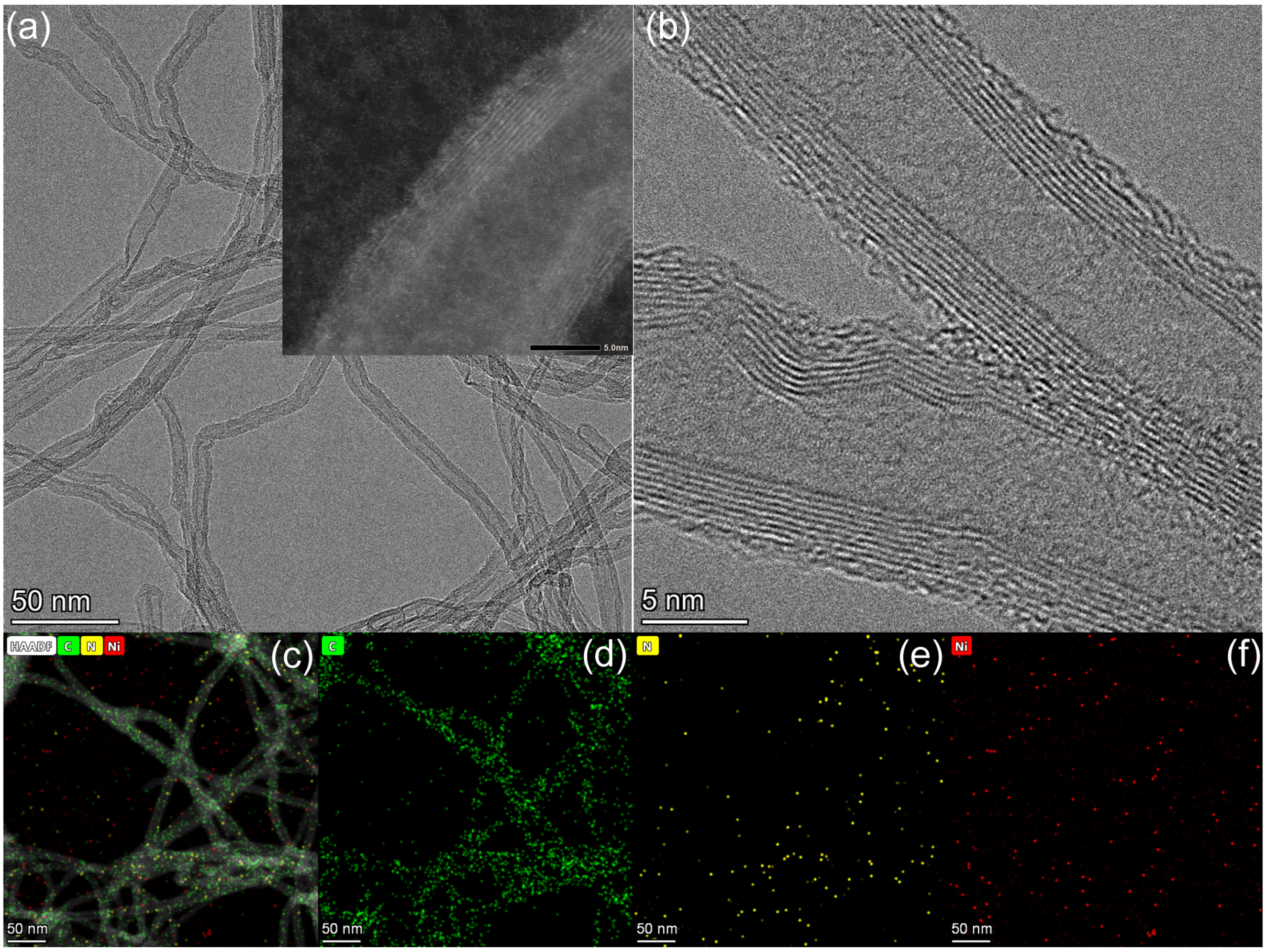


Figure S3. (a-b) Illustration of transmission electron microscopy (TEM) images of NiPhPC. Inset a: AC HAADF-STEM image of NiPhPC. (c to f) Energy-dispersive X-ray spectroscopy mapping images of NiPhPC.

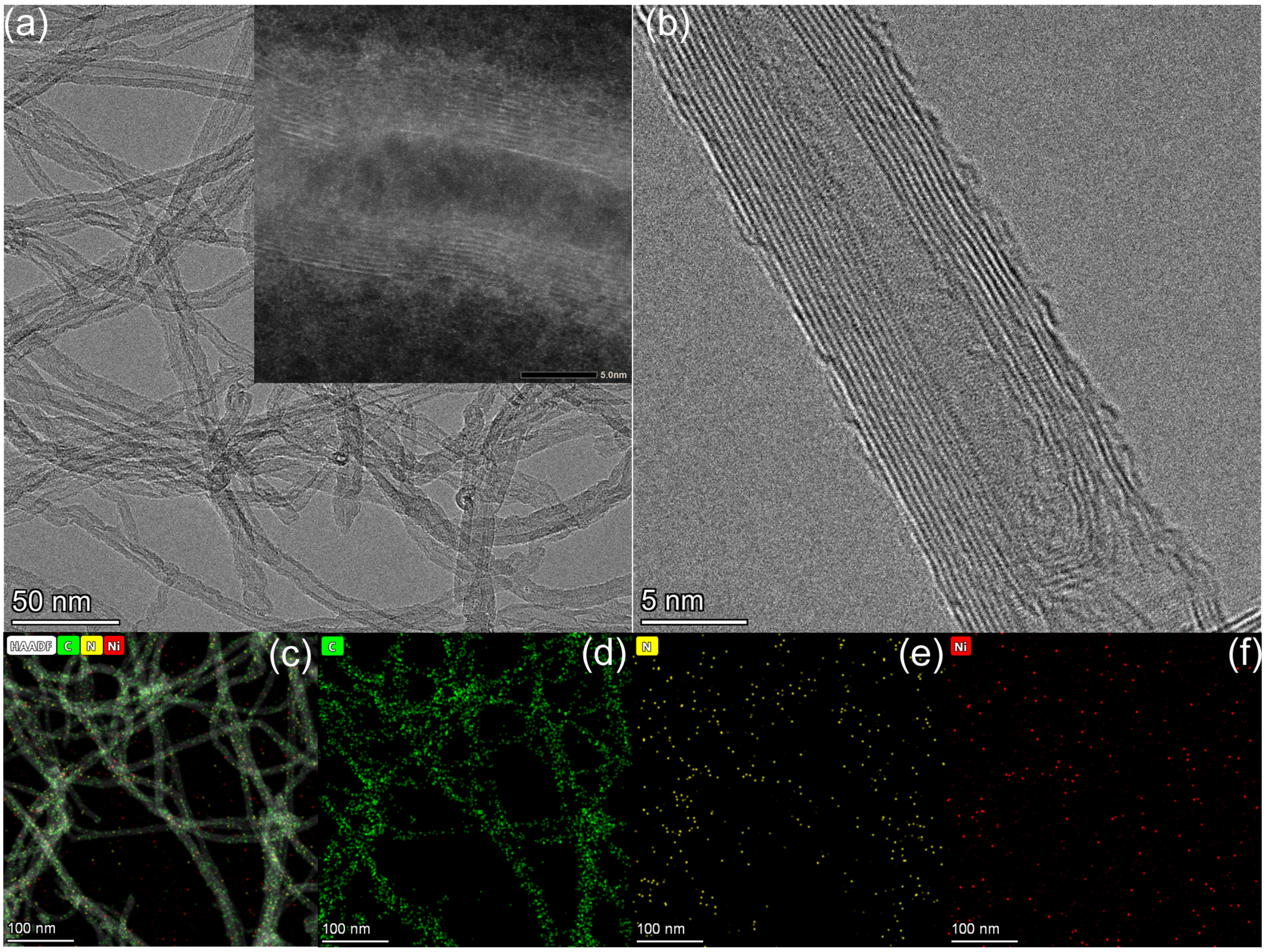


Figure S4. (a-b) Illustration of transmission electron microscopy (TEM) images of NiNPC. Inset a: AC HAADF-STEM image of NiNPC. (c to f) Energy-dispersive X-ray spectroscopy mapping images of NiPhPC.

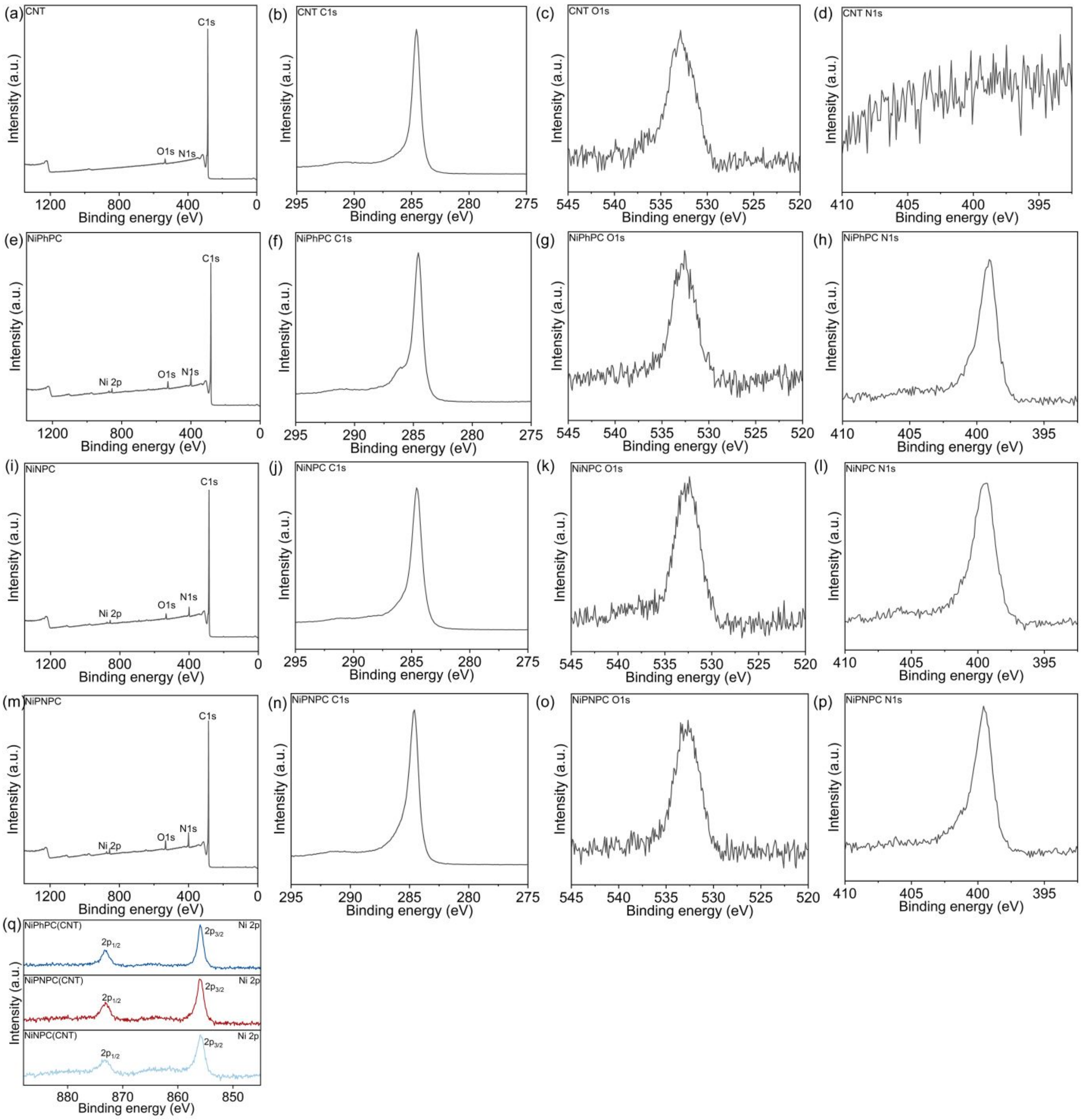


Figure S5. XPS spectra of NiPhPC(CNT), NiPNPC(CNT), and NiNPC(CNT).

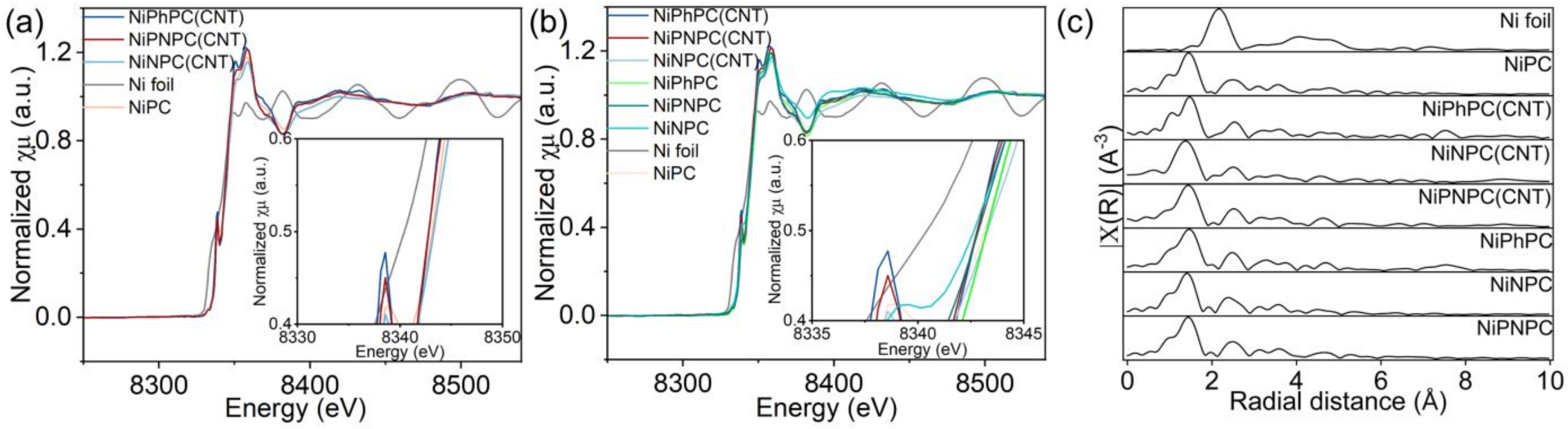


Figure S6. (a) Ni K-edge XANES data of NiPhPC(CNT), NiPNPC(CNT), and NiNPC(CNT). (b) Ni K-edge XANES data of NiPhPC(CNT), NiPNPC(CNT), and NiNPC, with the comparison of their NiPhPC, NiPNPC, and NiNPC molecular catalysts. (c) Fourier-transformed (FT) k3-weighted χ(k) function of the EXAFS spectra.

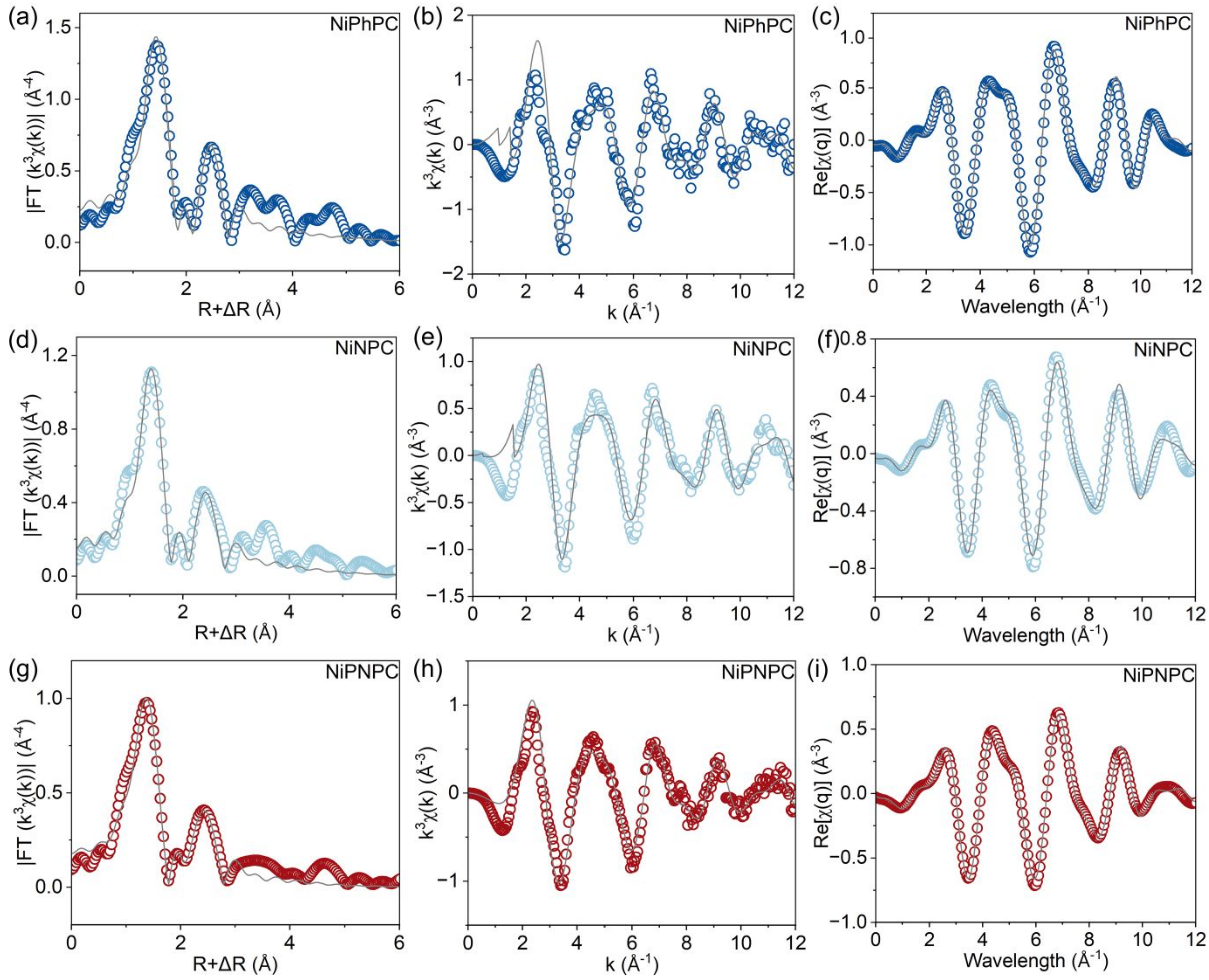


Figure S7. Fitting results of the R space, k space, and q space of EXAFS of NiNPC, NiPNPC, and NiPhPC.

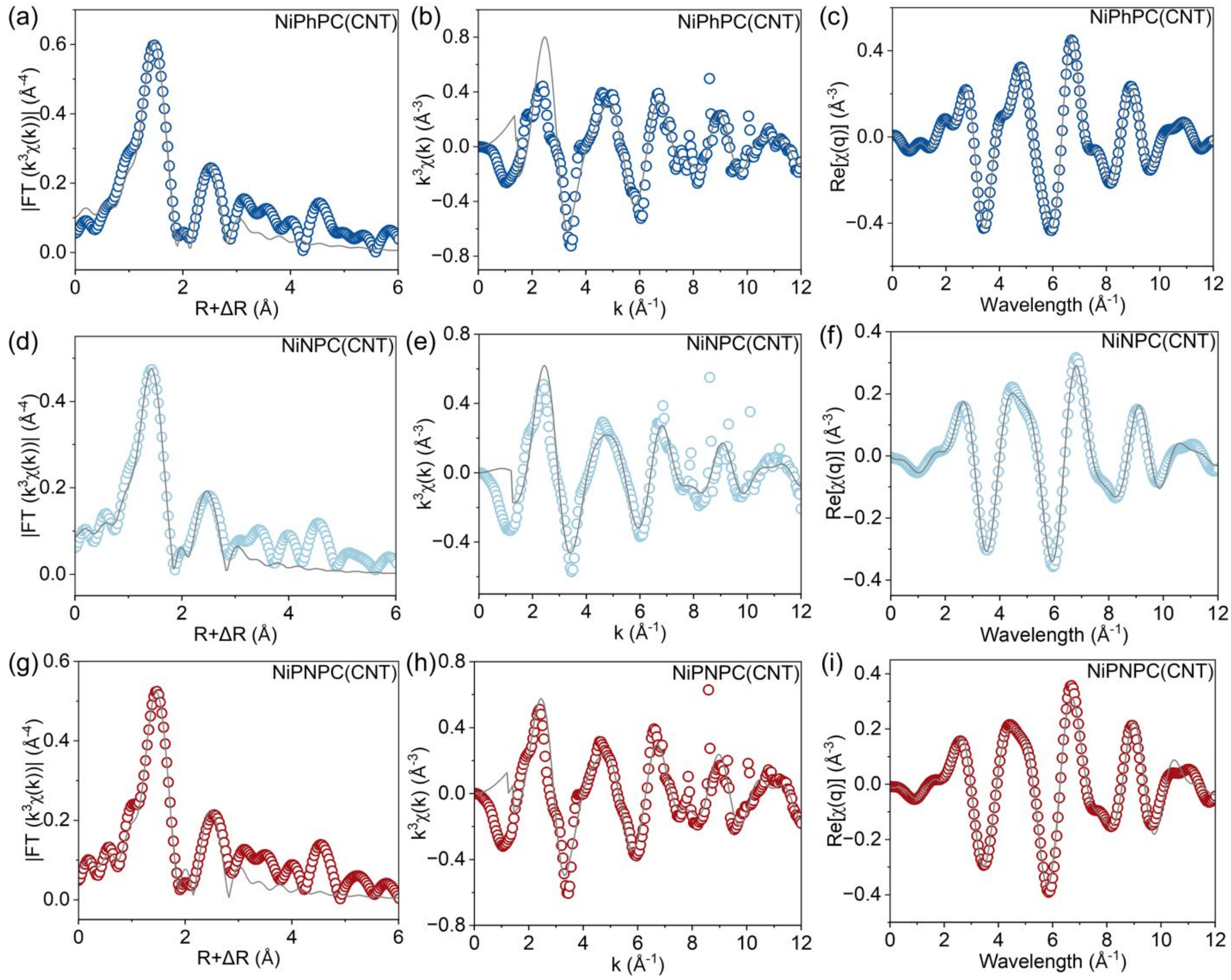


Figure S8. Fitting results of the R space, k space, and q space of EXAFS of NiNPC(CNT), NiPNPC(CNT), and NiPhPC(CNT).

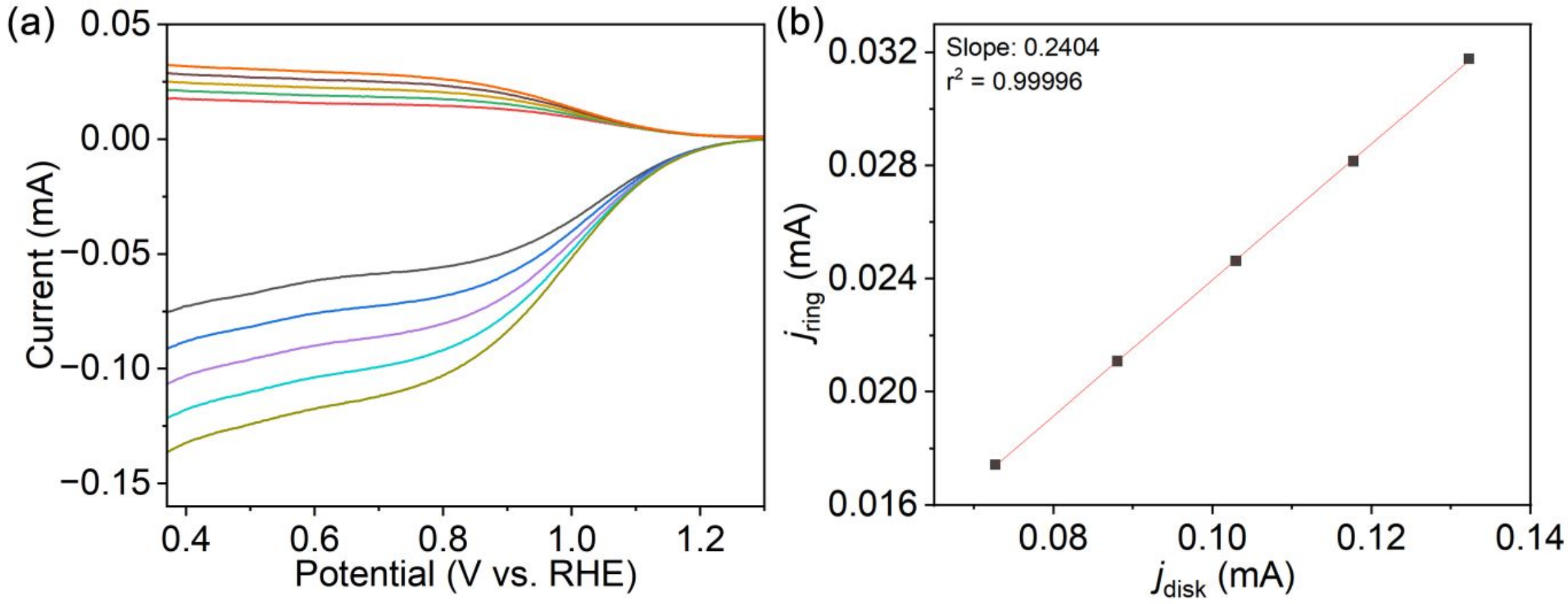


Figure S9. (a) LSV curves on a bare RRDE in 0.1 mol KOH with 10 mM $K_3Fe(CN)_6$ solution. Scan rate: 20 mV $s^{-1}$, Ering = 1.55 V vs. RHE. (b) Linear fitting of the diffusion-limited current density of ring and disk electrodes with different rotation speeds.

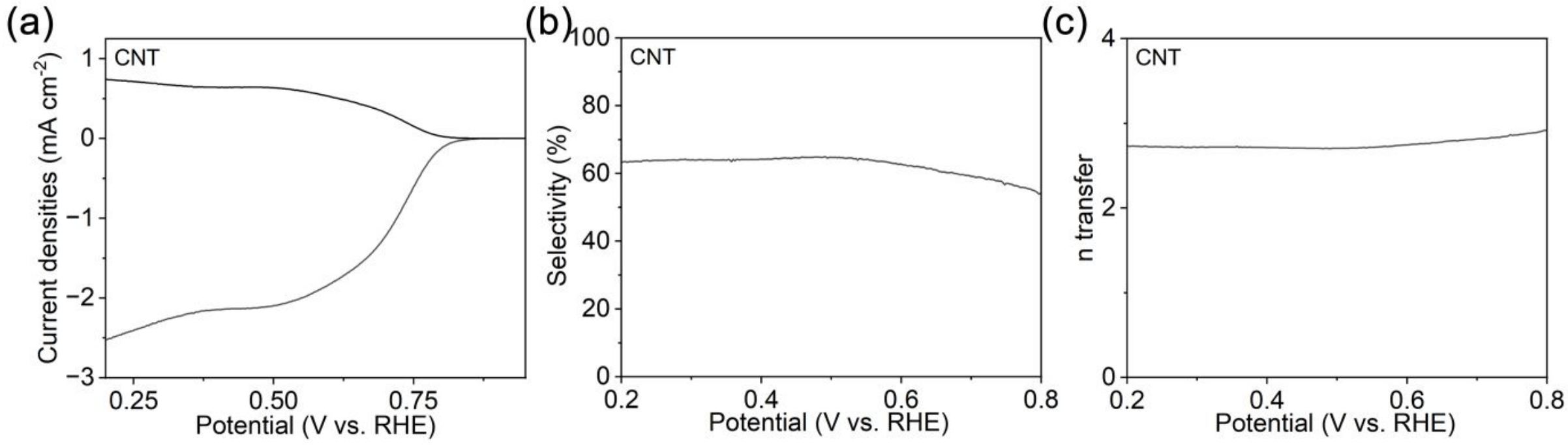


Figure S10. LSV curves (a), calculated selectivity (b), and n transfer (c) for CNT.

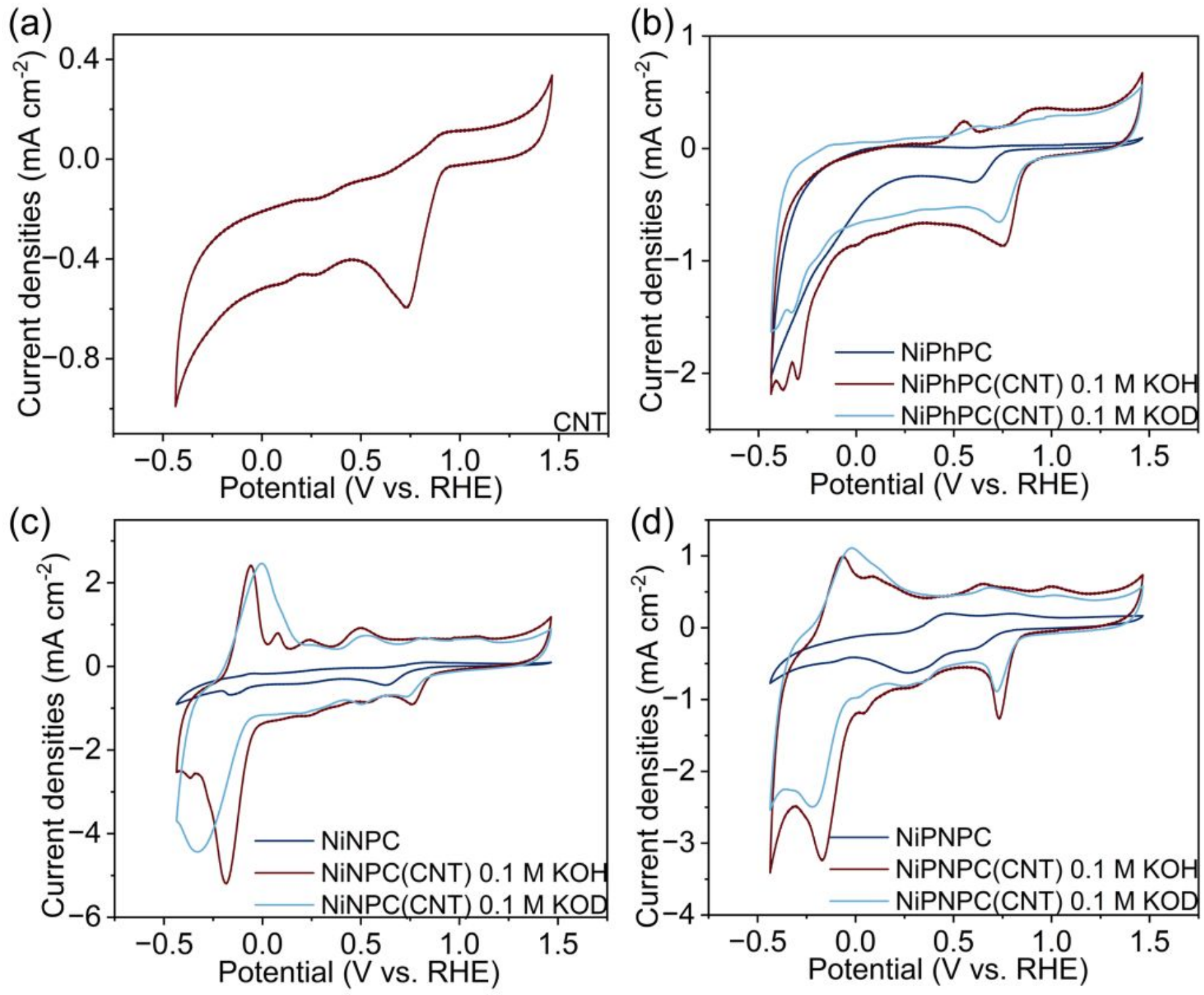


Figure S11. CV spectra comparison of (a) CNT, (b) NiPhPC, NiPhPC(CNT) (0.1 M KOH and 0.1 M KOD), (c) NiPNPC, NiPNPC(CNT) (0.1 M KOH and 0.1 M KOD), (d) NiPNPC, NiPNPC(CNT) (0.1 M KOH and 0.1 M KOD).

To distinguish the intrinsic properties of the molecular catalyst from the support effects, we have performed comparative CV measurements.[18, 19] As shown in Figure S11, the CV profiles of NiPhPC(CNT), NiNPC(CNT), and NiPNPC(CNT) exhibit well-defined redox features that are absent from bare CNT. This confirms that the observed electrochemical activity originates from the molecular species. The well-dispersed molecules on CNT also give rise to enlarged current densities compared to the pure molecules. Furthermore, the distinct differences in peak positions and current responses between 0.1 M KOH and 0.1 M KOD electrolytes for NiPhPC(CNT), NiNPC(CNT), and NiPNPC(CNT) provide additional evidence for hydrogenation processes occurring at active sites, further supporting the dominant role of the molecular catalyst in the composite.

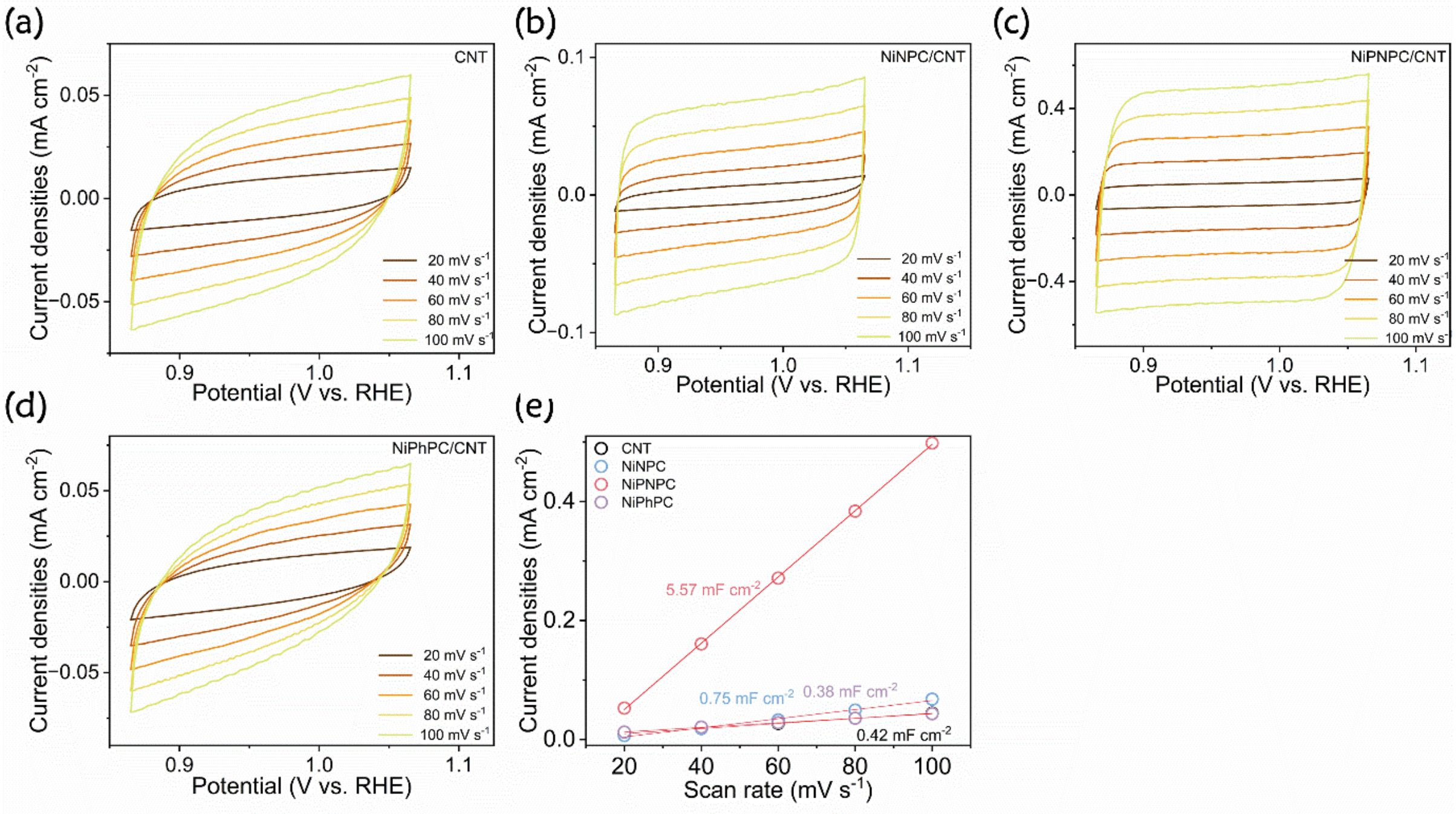


Figure S12. Cyclic voltammograms (CV) were measured for CNT (a), NiNPC(CNT) (b), NiPNPC(CNT) (c), and NiPhPC(CNT) (d) at different scan rates. (e) The linear relationship between the capacitive current varying scan rates for those nickel phthalocyanine derivatives.

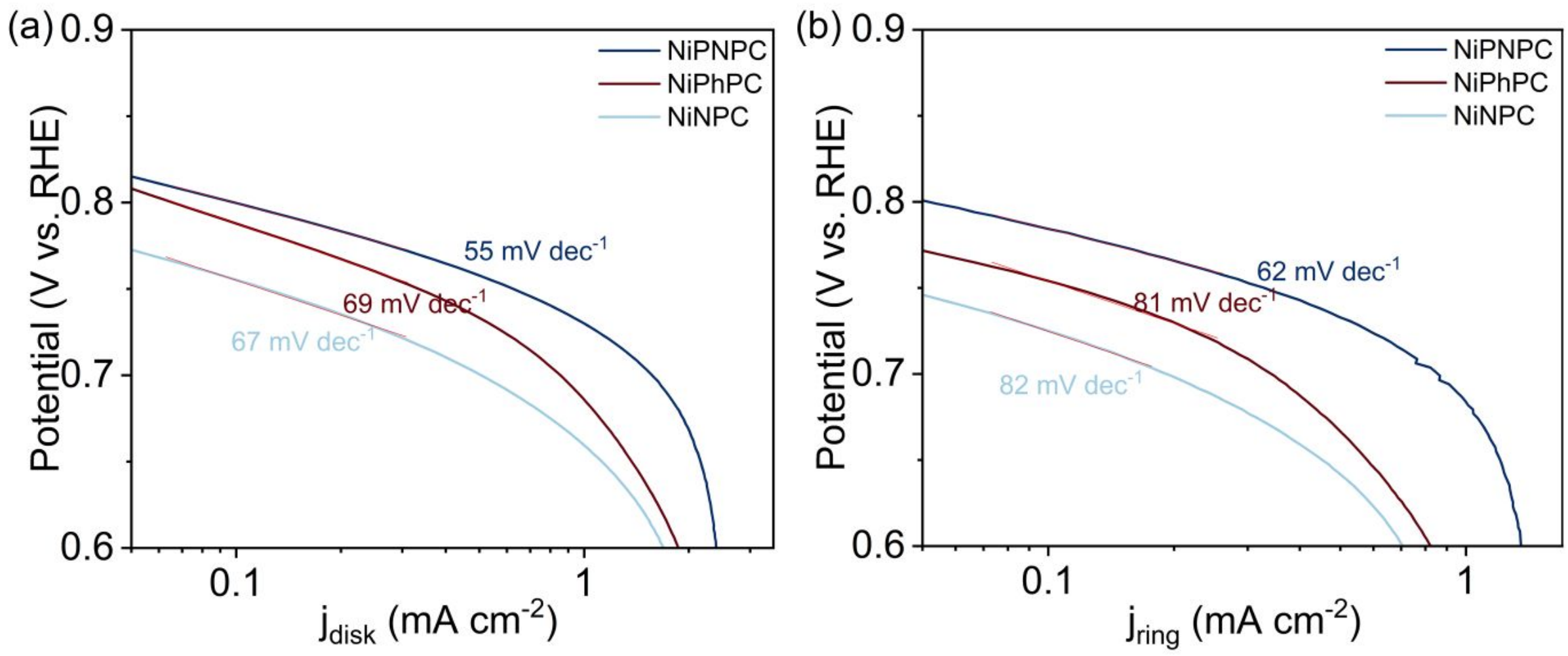


Figure S13. Tafel plots of NiNPC, NiPNPC, and NiPhPC calculated from $j_{\text{disk}}$ and $j_{\text{ring}}$.

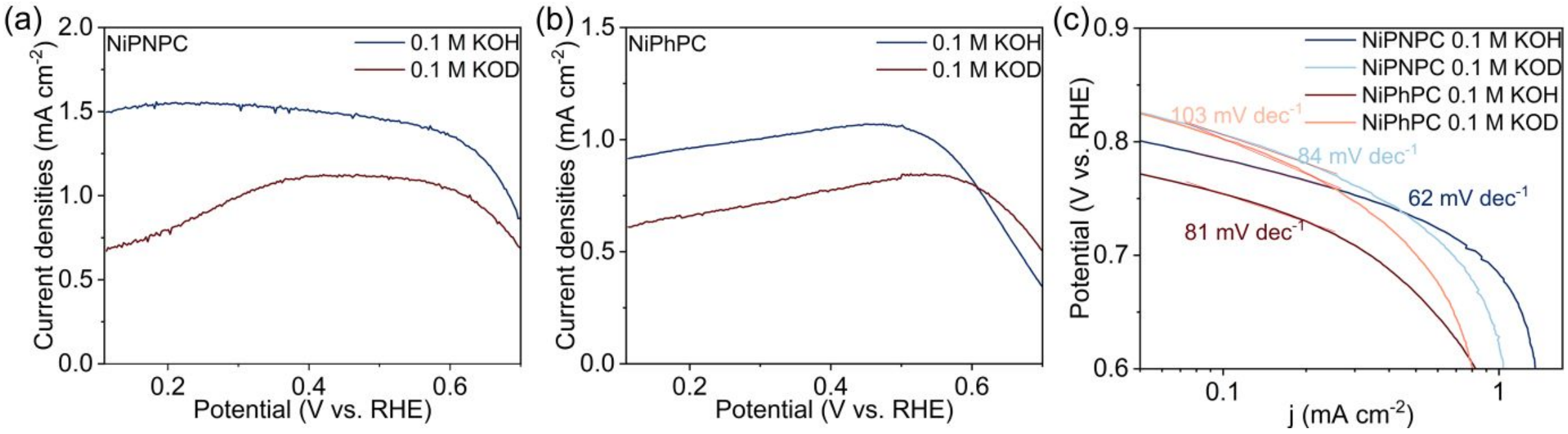


Figure S14. The kinetic isotope effect comparison of NiPNPC (a) and NiPhPC (b) for $H_2O_2$ production. (c) Calculated Tafel plots of NiPNPC and NiPhPC.

To further probe the kinetic isotope effect (KIE) and confirm the role of proton transfer, we performed Tafel analysis on both $H_2O$ and $D_2O$ electrolytes (0.1 M KOH vs. 0.1 M KOD). As shown in Figure S16, the Tafel slopes for NiPNPC increase from 62 mV dec$^{-1}$ (in KOH) to 84 mV dec$^{-1}$(in KOD), while those for NiPhPC increase from 81 mV dec$^{-1}$ to 103 mV dec$^{-1}$. The kinetic isotope effect for NiPNPC reaches approximately 1.5–2 at cathodic potentials below 0.4 V, significantly higher than that for NiPhPC (around 1.5 across almost the entire potential range). This demonstrates that proton transfer plays a more pronounced role in the rate-determining step for NiPNPC, consistent with the enhanced proton delivery enabled by the pyrazine-reorganized hydrogen-bonding network. This KIE comparison further supports that the proton transfer of NiPhPC is less efficient, consistent with its rigid water network (higher 4HB·$H_2O$ population) and lower $H_2O_2$ selectivity.

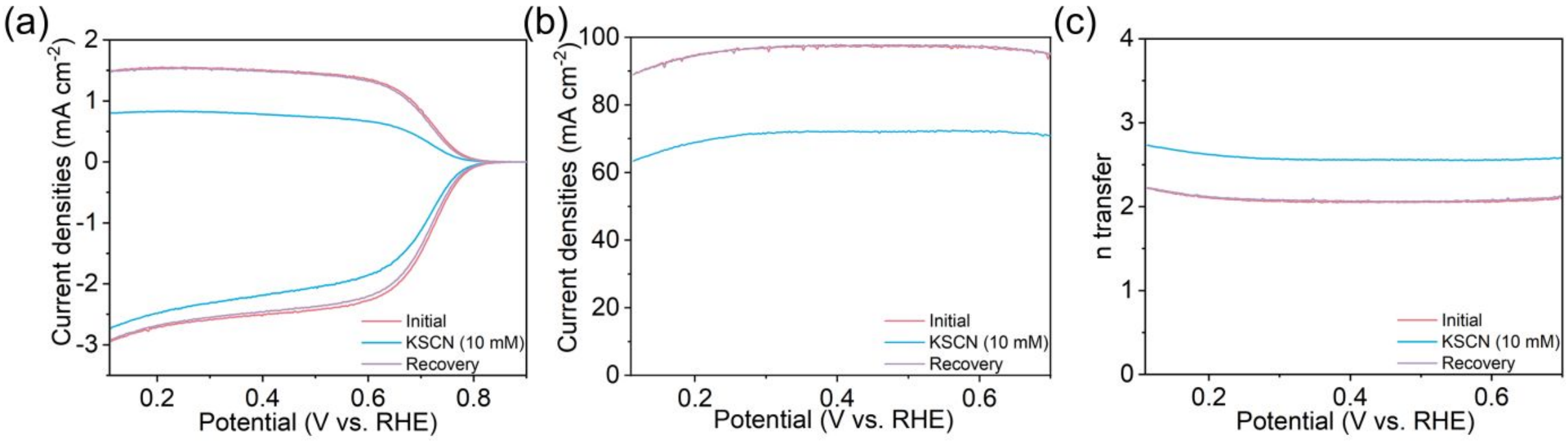


Figure S15. (a) Electrocatalytic LSV curves of NiPNPC with initial scan, addition of KSCN, and in recovery electrolyte. (b) Calculated selectivity comparison. (c) Calculated electron transfer numbers comparison.

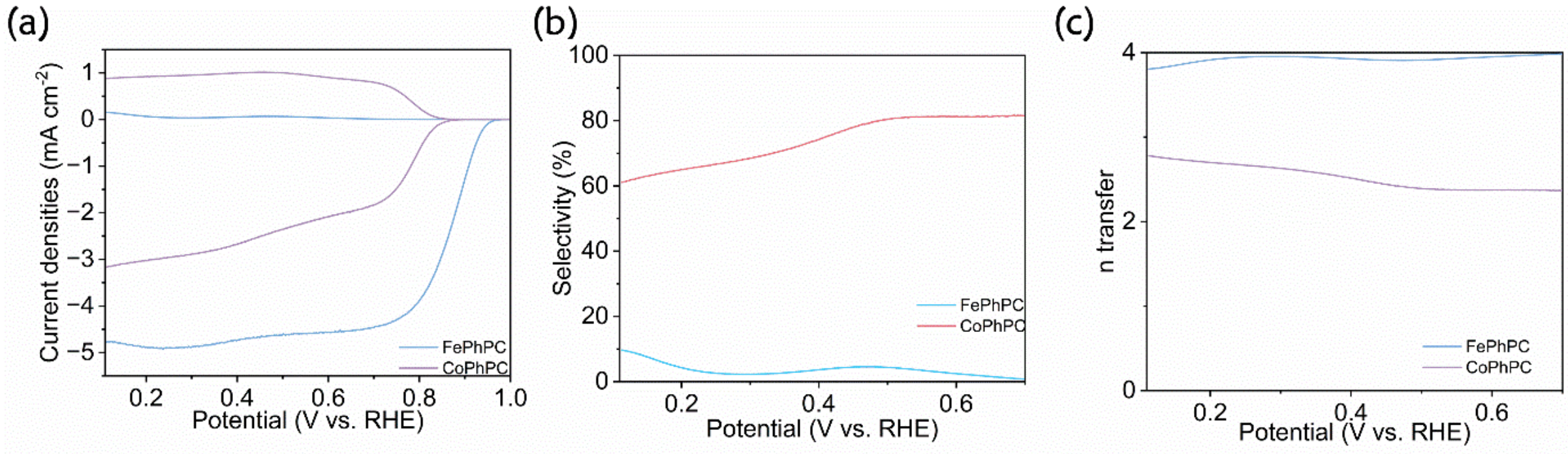


Figure S16. LSV curves (a), calculated selectivity (b), and n transfer (c) for FePhPC and CoPhPC.

Here we explain why we select NiPC derivatives to investigate the $H_2O_2$ production performance. The selection of Ni is not arbitrary but is grounded in both theoretical predictions and experimental validations from our group's prior work. First, DFT calculations on Fe, Co, and Ni-based systems revealed that Ni inherently favors the 2e$^-$ ORR pathway.[20] Second, we have experimentally demonstrated that nickel phthalocyanine derivatives serve as effective electrocatalysts for $H_2O_2$ production through dual regulation of molecular orbital and interfacial water.[3, 21] These findings are also consistent with independent theoretical studies identifying NiPC as a selective catalyst for 2e$^-$ ORR.[22-24] Thus, the choice of Ni as the starting point is well-justified by both our own prior work and the broader literature.

Besides, we have performed a systematic quantitative comparison of the FePNPC, CoPNPC, and NiPNPC catalysts using the electrochemical data already presented in Figure 3g–3i. The results are summarized in Table S3. Take the performance at 0.5 V vs. RHE as an example. The baseline $H_2O_2$ selectivity of NiPhPC is approximately 85.91%, which is significantly higher than those of CoPhPC (ca. 80.47%) and FePhPC (ca. 42.37%) (Figure S15). This is consistent with established literature that Ni–$N_4$ centers inherently favor the 2e$^-$ ORR pathway. Upon pyrazine incorporation, CoPNPC exhibits an absolute gain of approximately 8.5% relative to CoPhPC. Remarkably, NiPNPC displays a substantially larger absolute gain of approximately 11.1%, reaching 97.04%. FePNPC shows a gain of 20.3%, which is notable but still yields an absolute selectivity of only 62.65%, far below that of NiPNPC.

This comparison demonstrates that the full synergy of spin-crossover and hydrogen-bonding effects is uniquely realized in NiPNPC, where operando XES directly confirms the potential-driven spin crossover (Figure 5a). The substantially higher absolute selectivity of NiPNPC (97.04%) compared to CoPNPC (89.22%) and FePNPC (62.65%) highlights the unique effectiveness of the Ni–pyrazine synergy in promoting the 2e$^-$ ORR pathway. We indeed note that Co is known to adopt an intermediate ($S = 1/2$) or high-spin ($S = 3/2$) state in phthalocyanine systems. This is supported by DFT calculations from the literature, where a study of Me–$N_4$ (Me = Fe, Co, Ni) clusters showed that $O_2$ can be chemisorbed on Co–$N_4$ and Fe–$N_4$ clusters but not on Ni–$N_4$ clusters in their ground state,[25] indicating that Ni–$N_4$ catalysts require an external driving force (such as potential-driven spin crossover) to activate $O_2$, while Co–$N_4$ already bind $O_2$. However, in our present work, the performance enhancement for CoPNPC and FePNPC remains limited compared to NiPNPC. We do not have operando XES data for CoPNPC, so we refrain from making definitive statements about its spin-state evolution. Therefore, we focus our mechanistic investigation on the Ni system, where the activity towards $H_2O_2$ production is most pronounced.

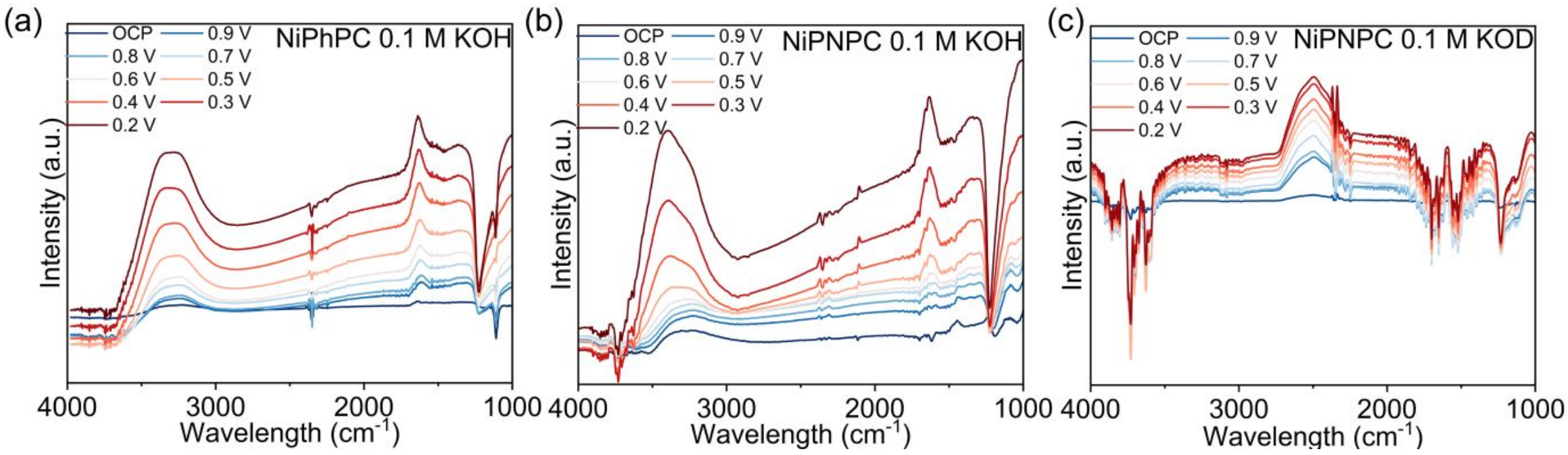


Figure S17. In situ raw ATR-SEIRAS spectra comparison of NiPNPC and NiPhPC.

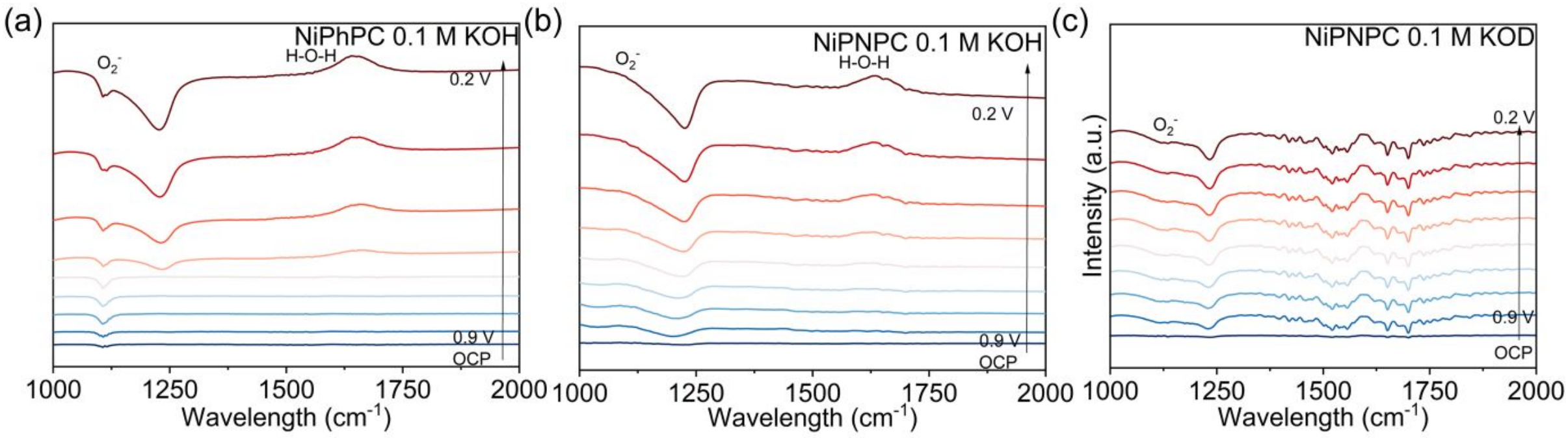


Figure S18. In situ ATR-SEIRAS spectra comparison of NiPNPC and NiPhPC.

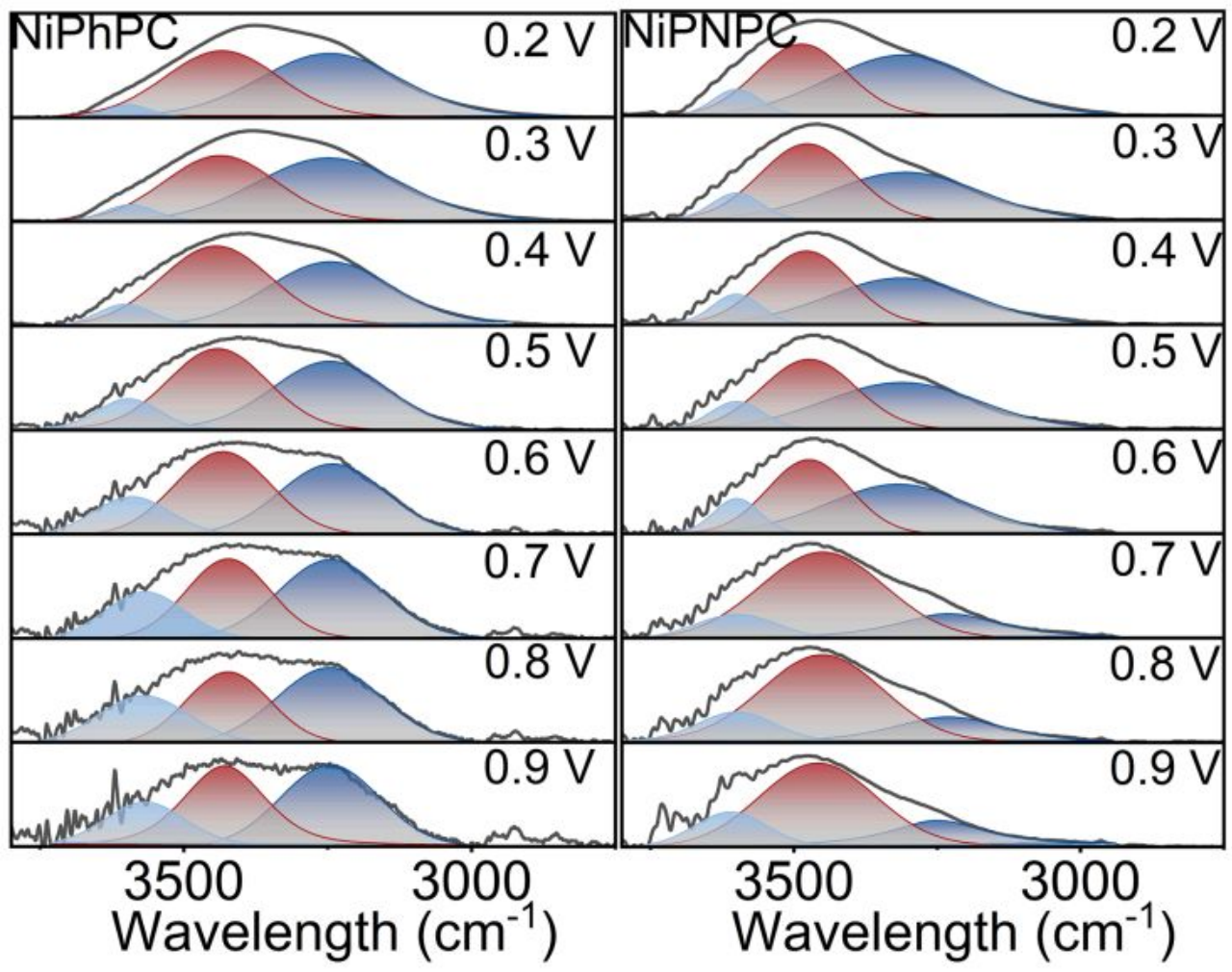


Figure S19. In situ FTIR spectra of the O-H stretching vibrational mode of NiPNPC and NiPhPC.

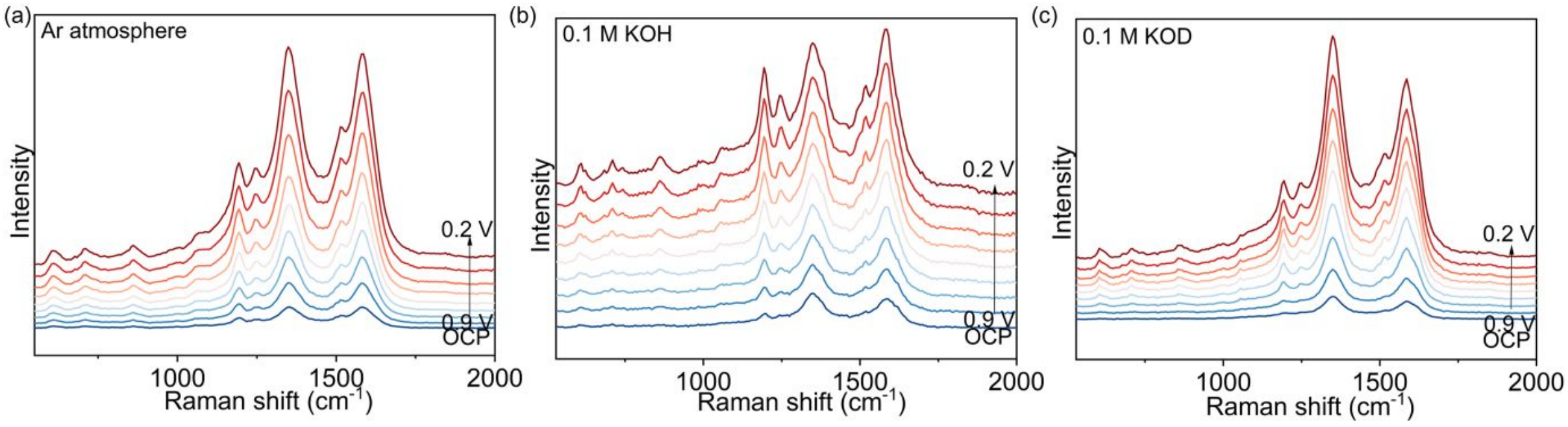


Figure S20. In situ Raman spectra of NiPNPC.

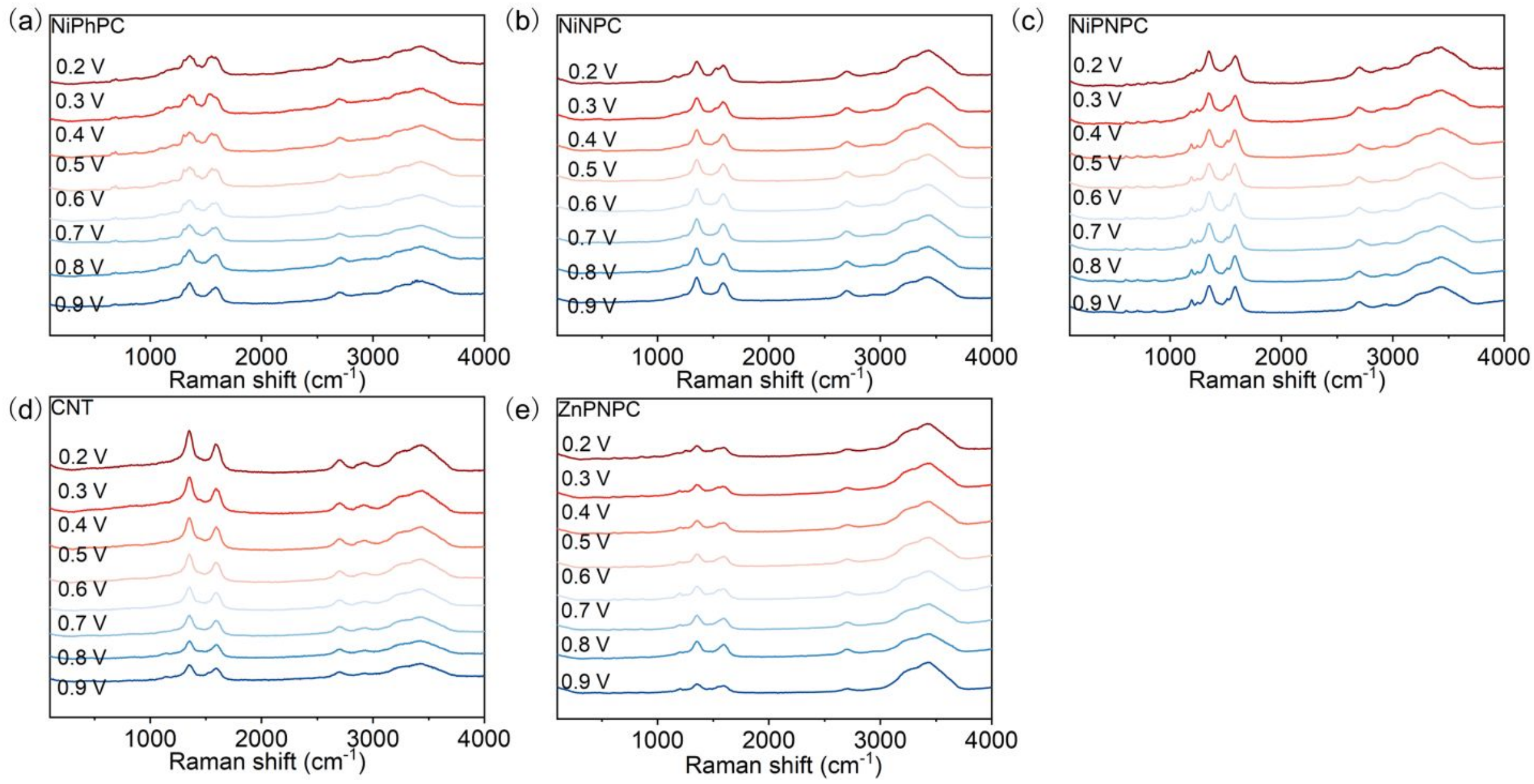


Figure S21. Potential-dependent raw Raman spectra of NiPhPC, NiNPC, NiPNPC, CNT, and ZnPNPC.

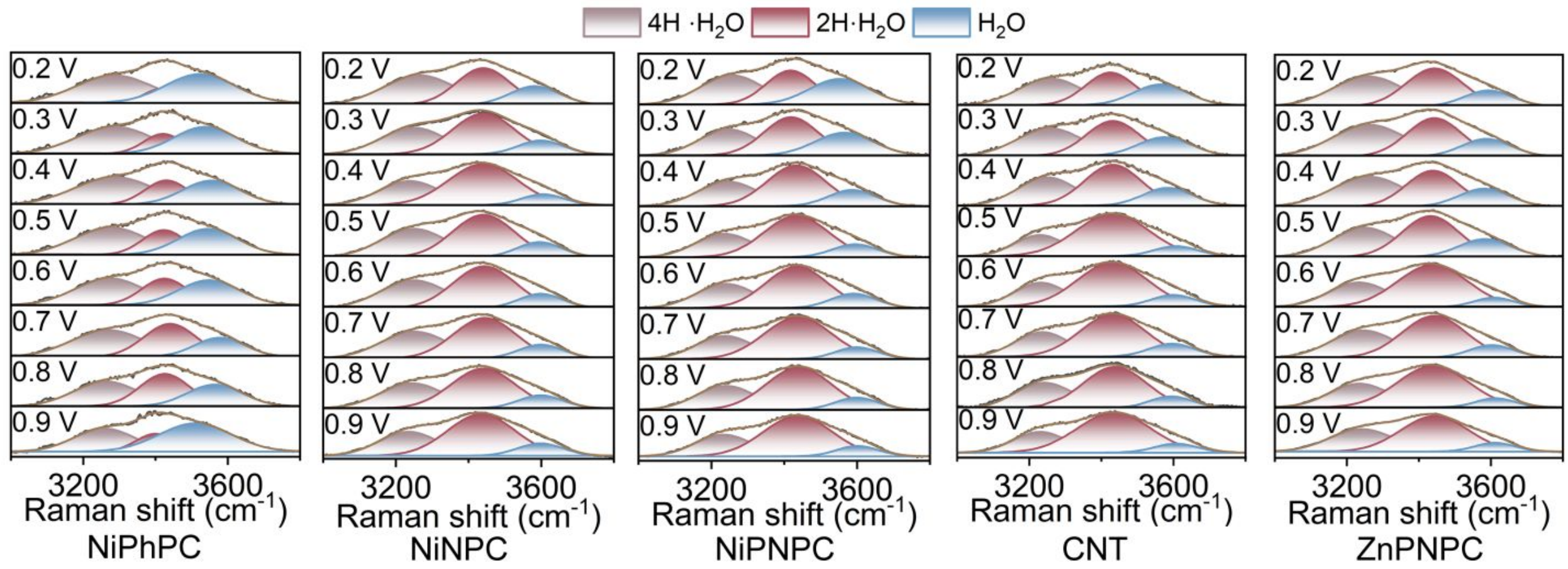


Figure S22. In situ Raman spectra of the O-H stretching vibrational mode of NiPhPC, NiNPC, NiPNPC, CNT, and ZnPNPC.

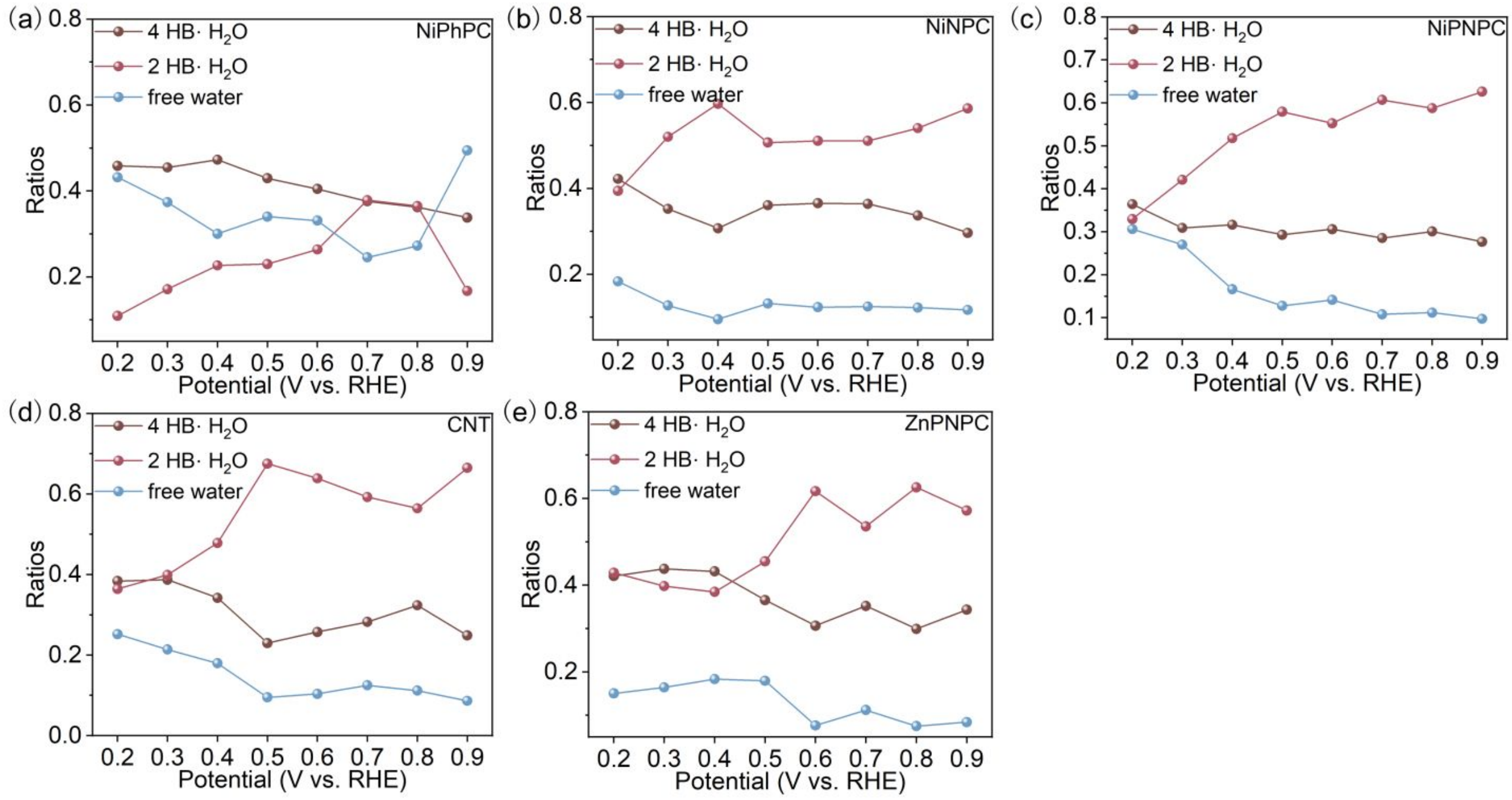


Figure S23. Calculated ratio of the interfacial water for NiPhPC, NiNPC, NiPNPC, CNT, and ZnPNPC.

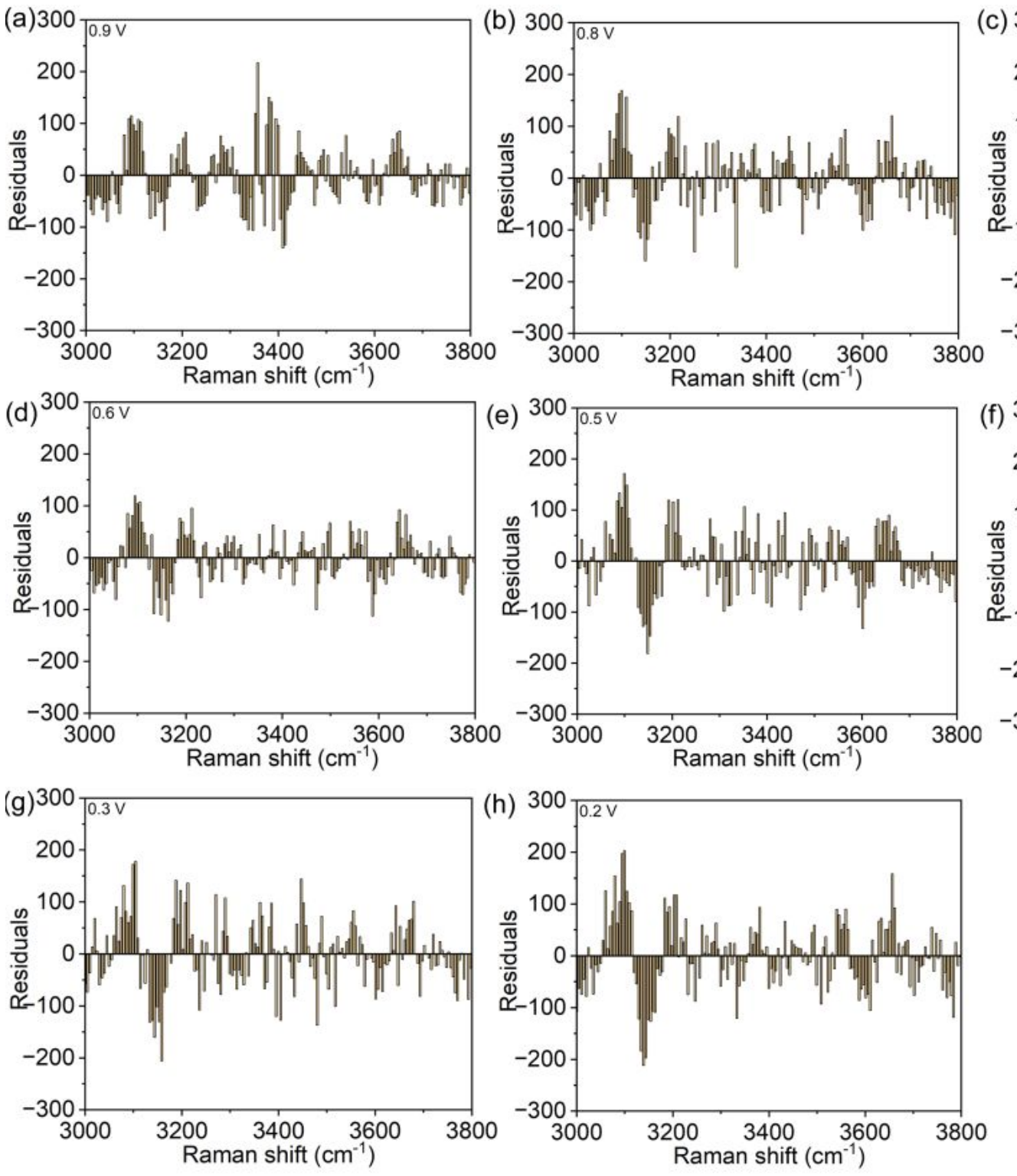


Figure S24. Fitting residuals of O-H stretching vibrational for NiPhPC.

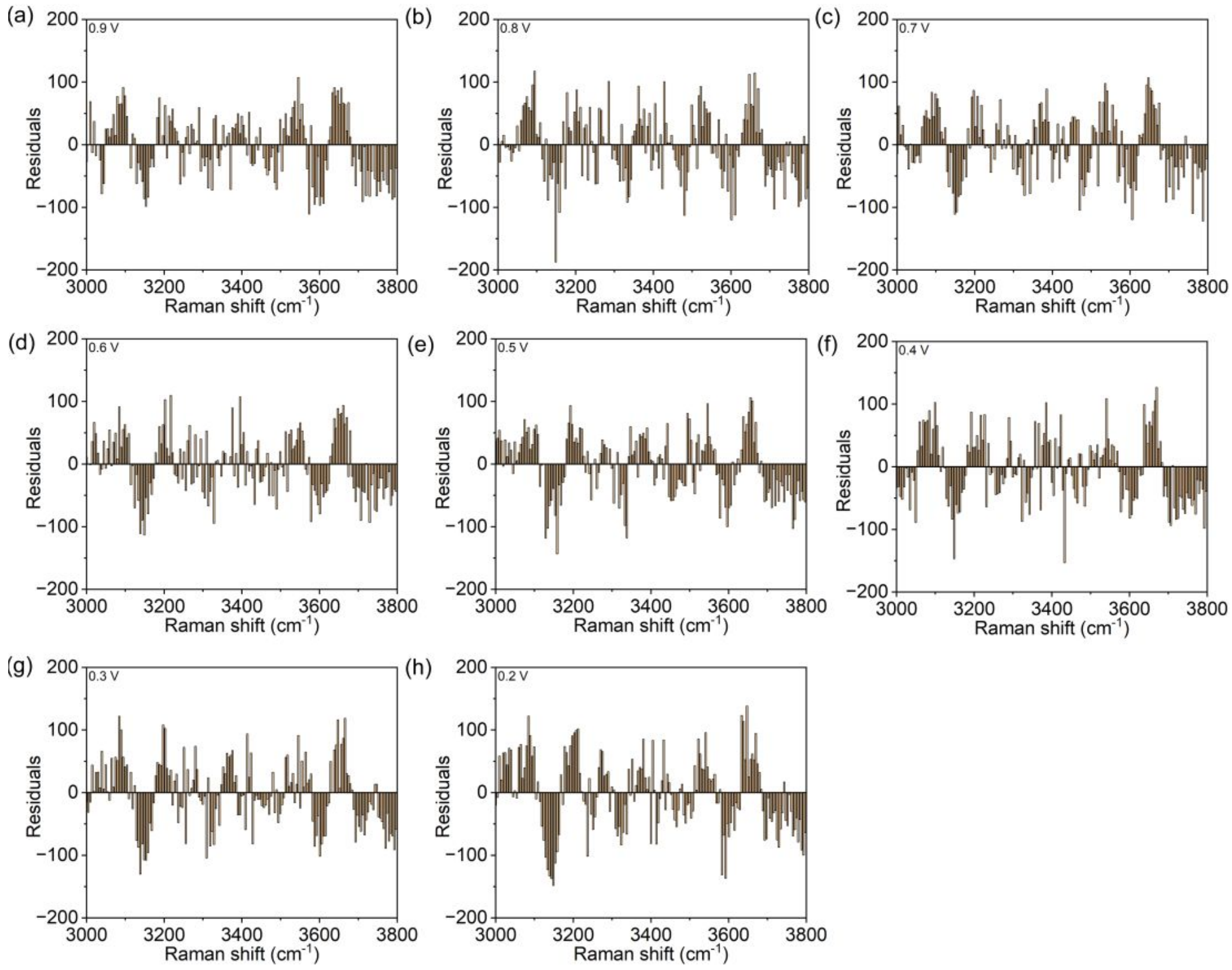


Figure S25. Fitting residuals of O-H stretching vibrational for NiNPC.

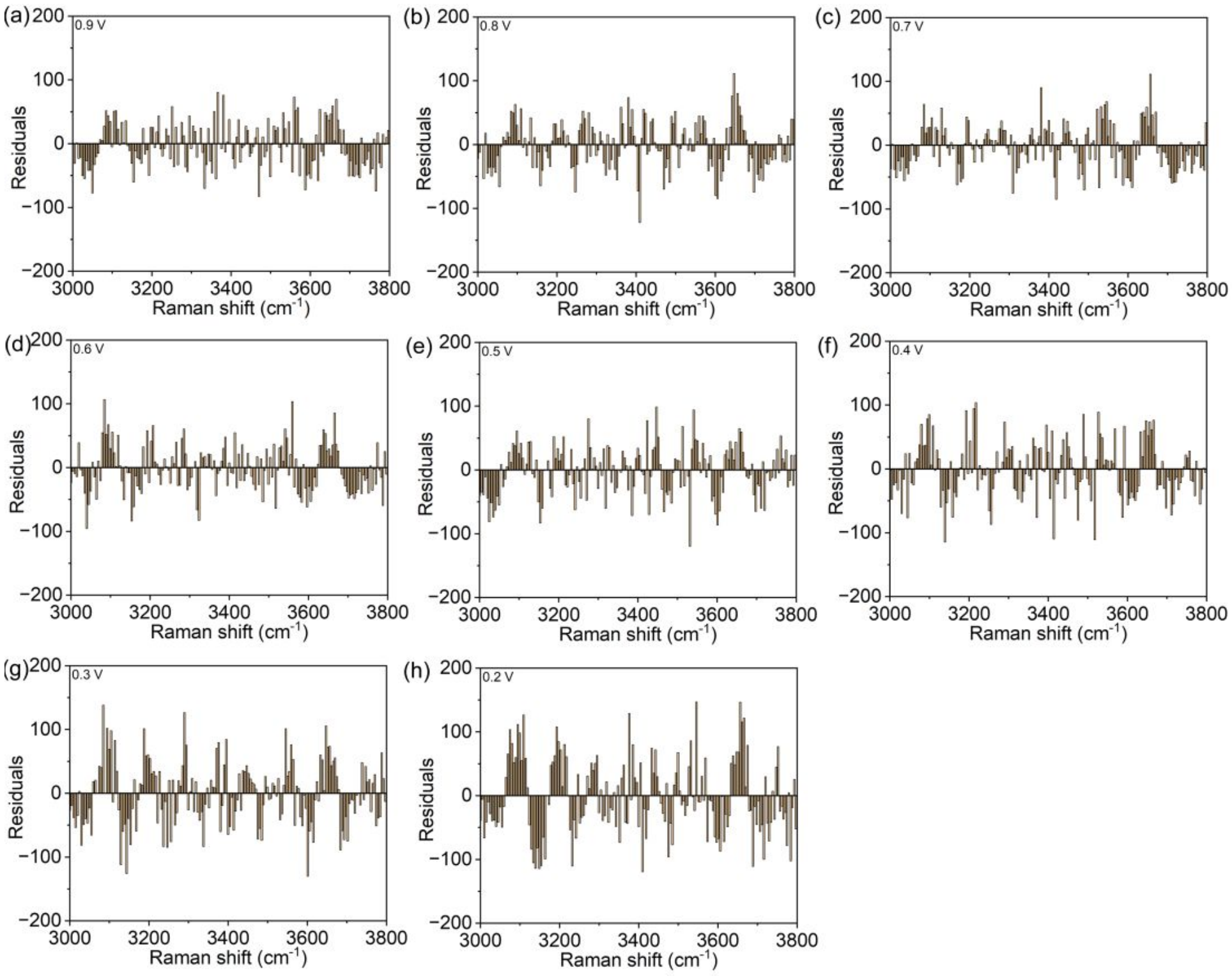


Figure S26. Fitting residuals of O-H stretching vibrational for NiPNPC.

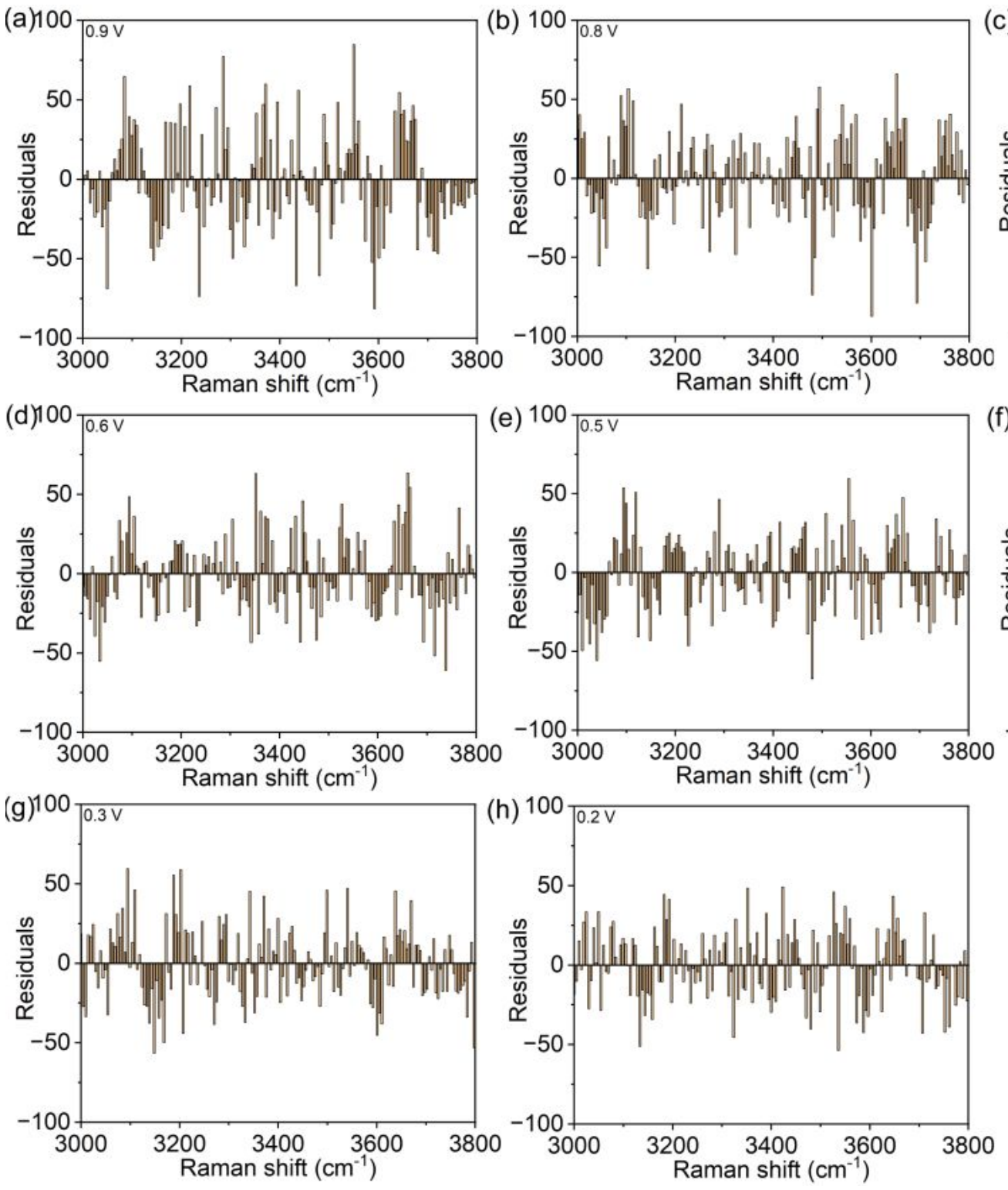


Figure S27. Fitting residuals of O-H stretching vibrational for CNT.

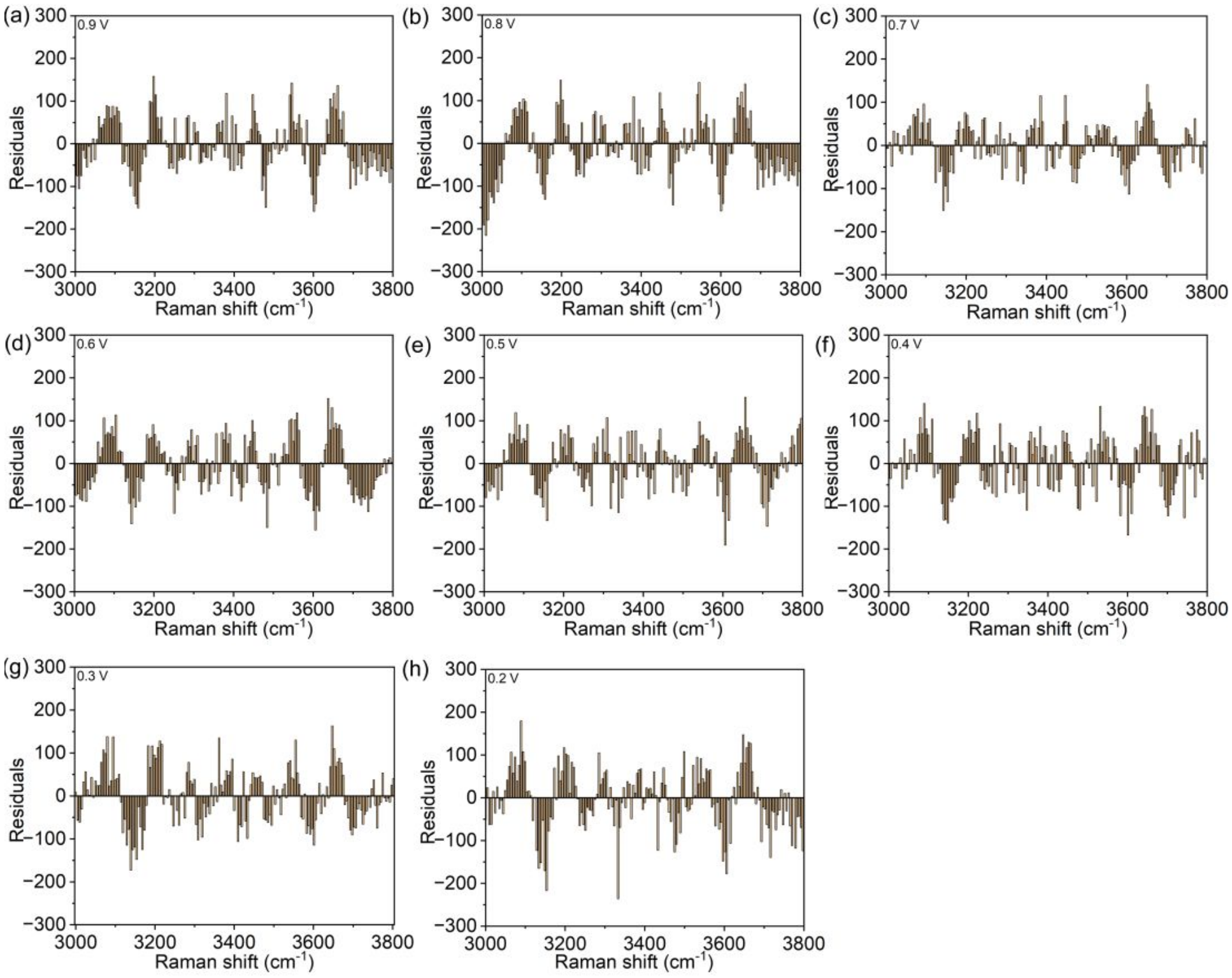


Figure S28. Fitting residuals of O-H stretching vibrational for ZnPNPC.

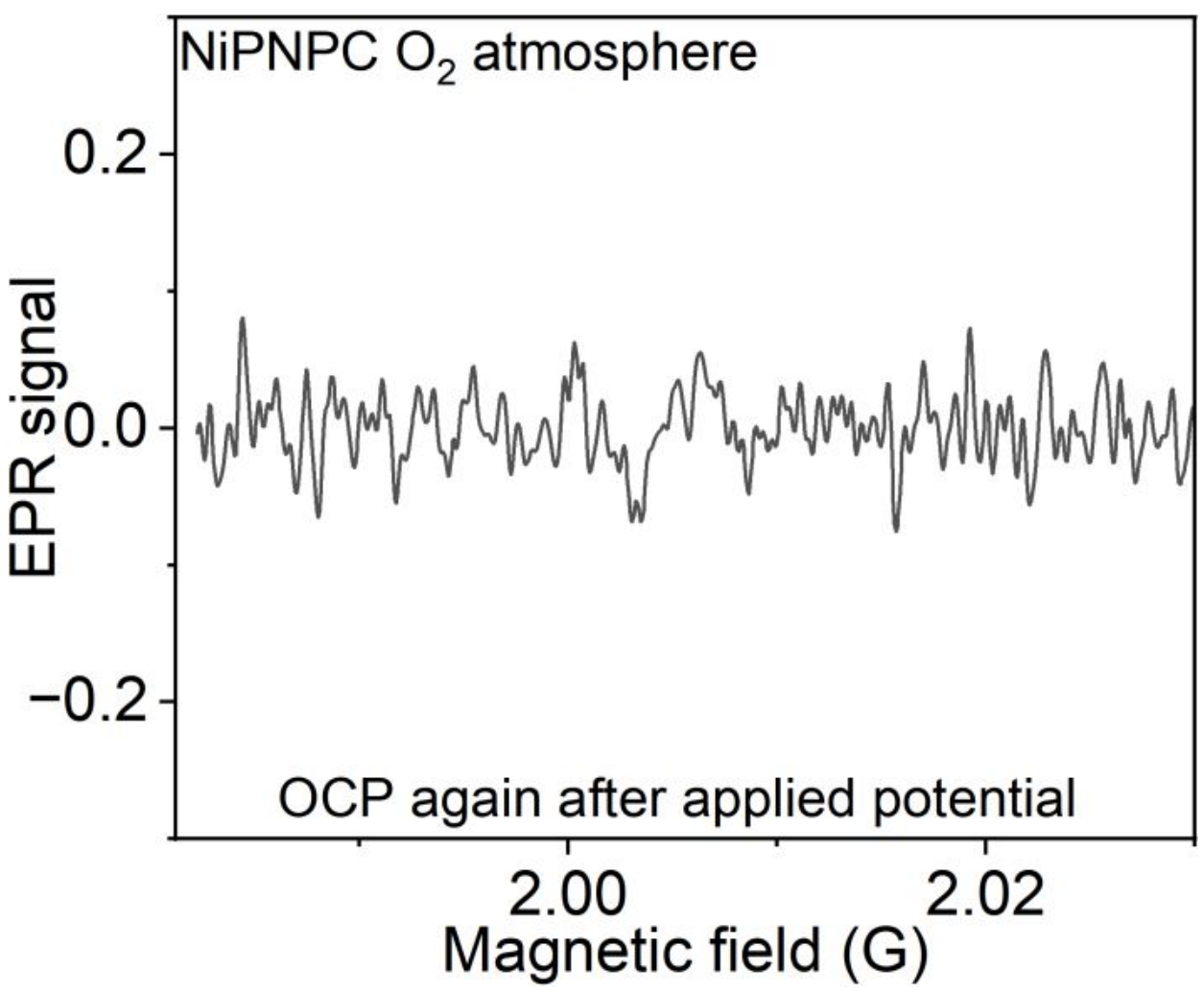


Figure S29. EPR signal of NiPNPC electrode after reactions.

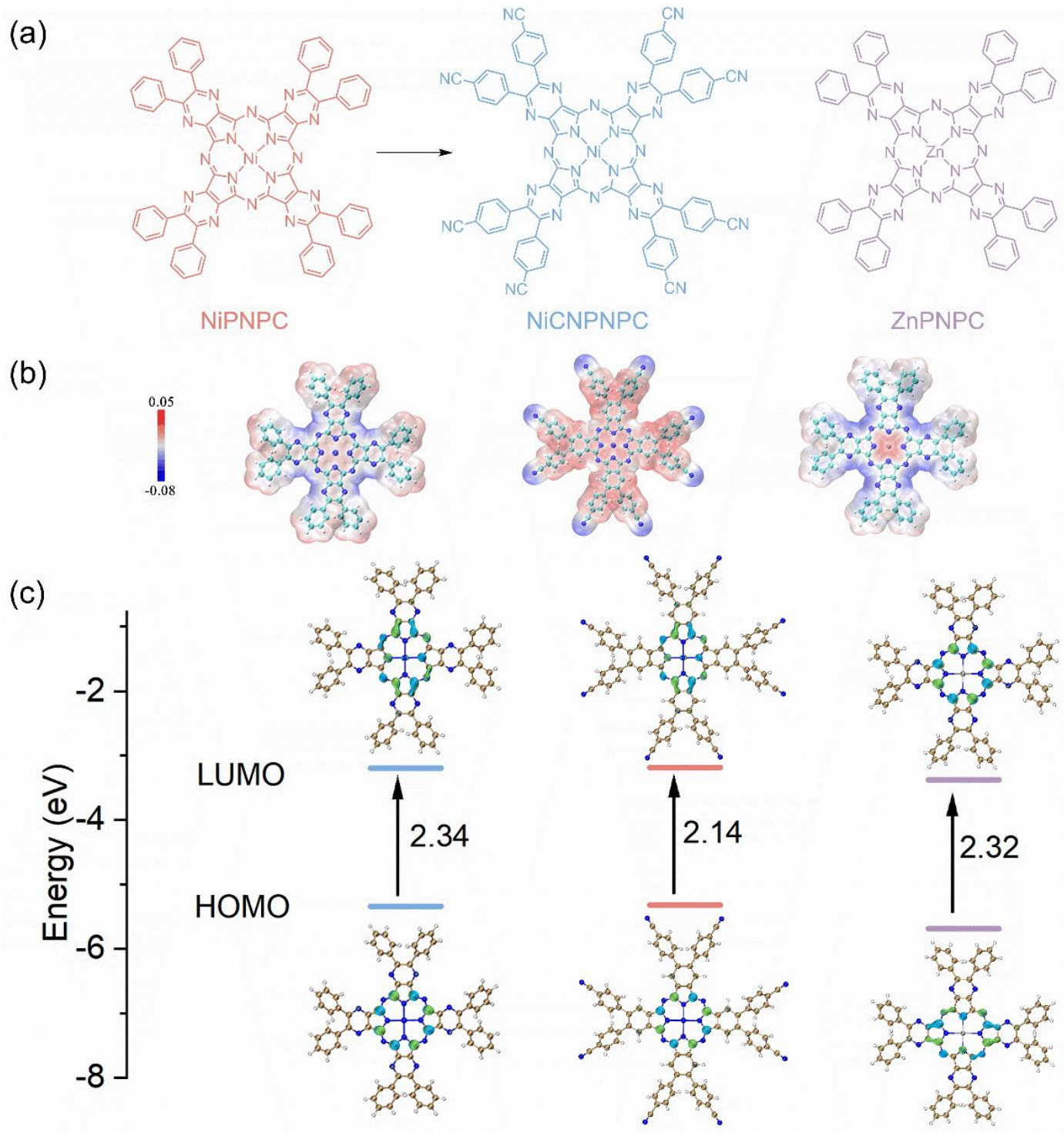


Figure S30. (a) Illustrating the structure of. (b) Calculated electrostatic potentials (ESP) for the NiPC derivatives. (c) Calculated HOMO-LUMO orbitals.

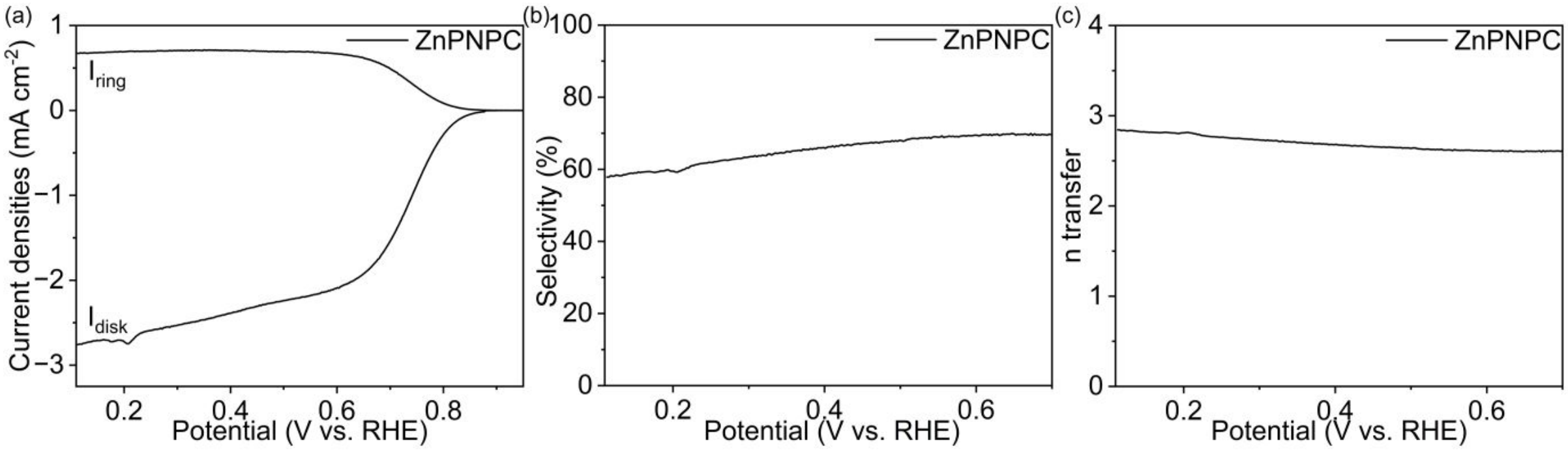


Figure S31. (a) Electrocatalytic LSV curves at the ring-disk electrodes. (b) Calculated selectivity for production of $H_2O_2$. (c) Calculated electron transfer numbers.

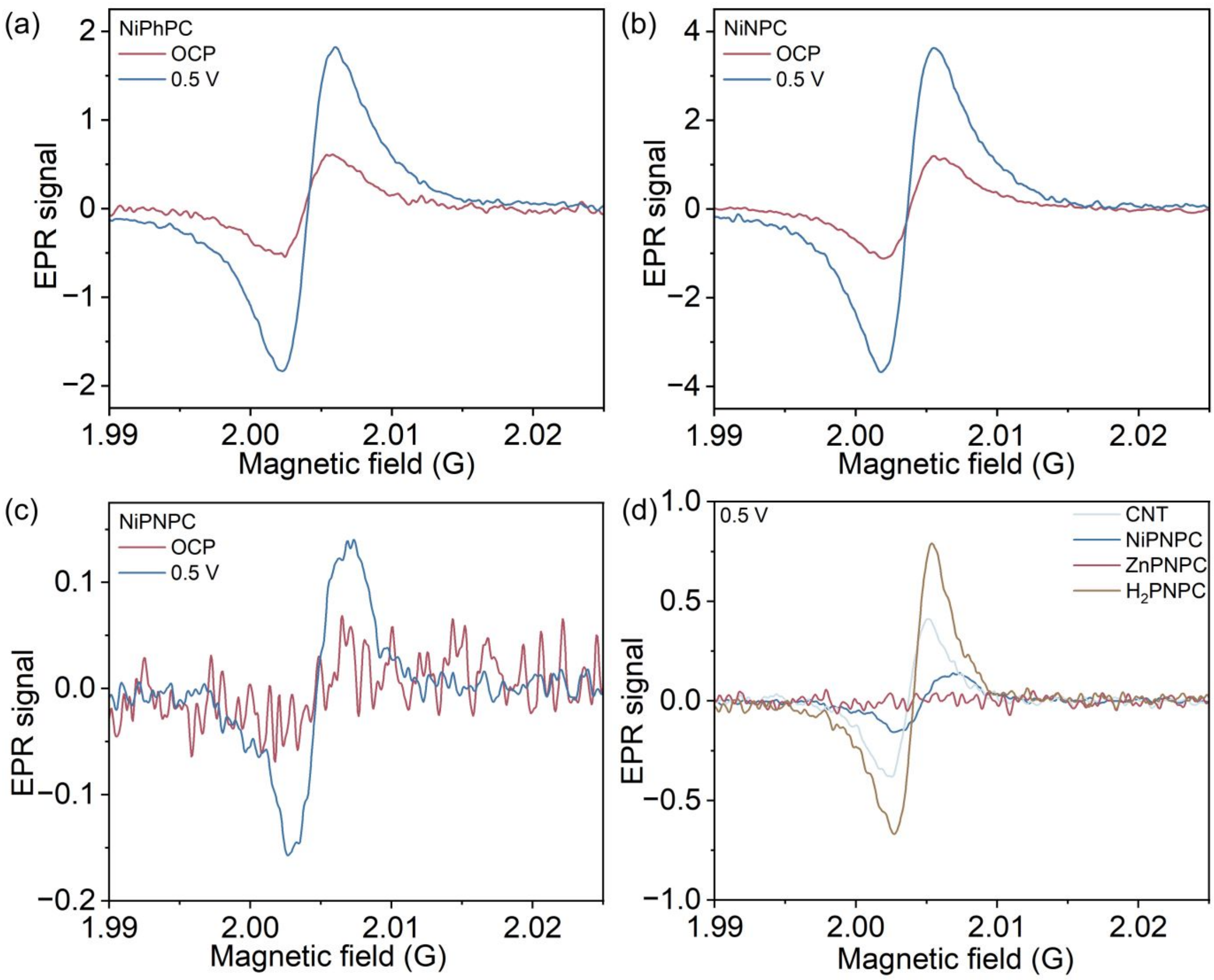


Figure S32. (a) EPR signal comparison of NiPhPC at OCP and 0.5 V vs. RHE. (b) EPR signal comparison of NiNPC at OCP and 0.5 V vs. RHE. (c) EPR signal comparison of NiPNPC at OCP and 0.5 V vs. RHE. (d) EPR signal comparison of CNT, NiPNPC, ZnPNPC, $H_2$PNPC at 0.5 V vs. RHE.

We have performed EPR measurements on control samples under identical conditions. As shown in Figure S32, it is found that CNT alone shows a signal, which likely originates from the carbon defects. We also synthesized the metal-free pyrazine-phthalocyanine ligand ($H_2$PNPC), and EPR measurements on $H_2$PNPC(CNT) exhibit an obvious EPR response. However, we also prepared ZnPNPC on CNT, as Zn(II) is $d^{10}$ and has no EPR response. ZnPNPC with the same pyrazine ligand indeed has no EPR response, demonstrating that the pyrazine ligand from the complex does not generate EPR signal under our experimental conditions. Both NiPhPC and NiNPC exhibit static EPR signals that are present even at OCP and show enhancement upon applying a potential. These signals are not considered to correlate with catalytic activity as NiPhPC and NiNPC show relatively poor $H_2O_2$ selectivity compared to NiPNPC. For NiPNPC, a clear signal emerges upon applying a potential (Figure 4h, Figure S32c, Figure S33). This behavior is attributed to a potential-driven spin crossover at the Ni center, consistent with our operando XES data, while NiPhPC does not exhibit a significant potential dependent XES shift during the tests.

Based on above data, we have performed a stepwise exclusion analysis to further clarify the origin of the signals. (a) From Figure S32, CNT alone exhibits a potential insensitive background signal. This signal is not observed in CNT supported ZnPNPC or NiPNPC after immbilization, confirming that the CNT support does not contribute to the potential dependent response. (b) The metal free ligand H2PNPC exhibits an EPR signal, but ZnPNPC shows almost no signal. This demonstrates that ligand centered radicals are quenched upon metal coordination and are not the source of the potential

dependent EPR response in NiPNPC. (c) ZnPNPC shows no potential dependent EPR signal, whereas NiPNPC shows a clear potential dependent response. This directly demonstrates that the Ni center is essential. (d) NiPhPC and NiNPC already exhibit EPR signals at OCP, in contrast, NiPNPC shows no detectable signal at OCP, and a clear signal emerges only upon applying negative potentials. This phenomenon suggests a potential-driven spin crossover at the Ni center of NiPNPC, which is also confirmed by the operando XES data (Figure 5a), where NiPNPC exhibits a clear potential-driven spectral shift towards the NiO reference (high spin) reference, while NiPhPC shows no such significant shift. This interpretation is further supported by Ni K edge XANES measurements (Figure 2b, Figure S6), which show that the Ni center in NiPhPC, NiNPC, and NiPNPC have nearly identical electronic structures. The dramatic differences in EPR intensity therefore reflect differences in spin density distribution within the molecules, not differences in the Ni center itself.

The combined evidence from CNT, ZnPNPC, metal free ligand, XES, and XANES consistently supports the assignment of the NiPNPC potential dependent EPR response to Ni centered spin crossover. The signals in NiPhPC and NiNPC may be attributed to static LMCT backgrounds arising from their structural features, while NiPNPC uniquely exhibits a clean, potential driven Ni centered signal. This interpretation is fully consistent with the operando XES data (Figure 5a) and the superior $2e^-$ ORR activity of NiPNPC.

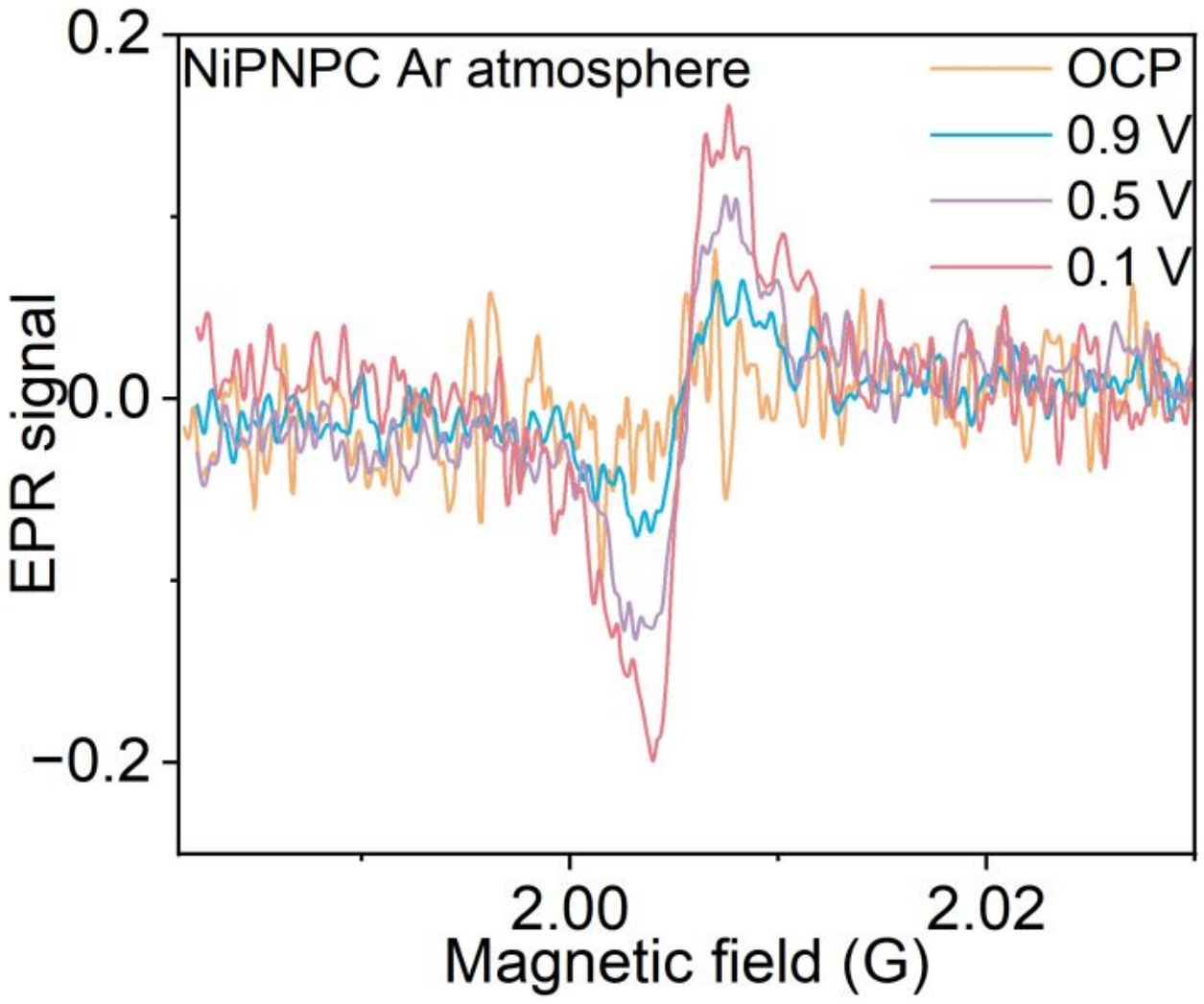


Figure S33. The EPR signals of the NiPNPC electrodes under different potentials.

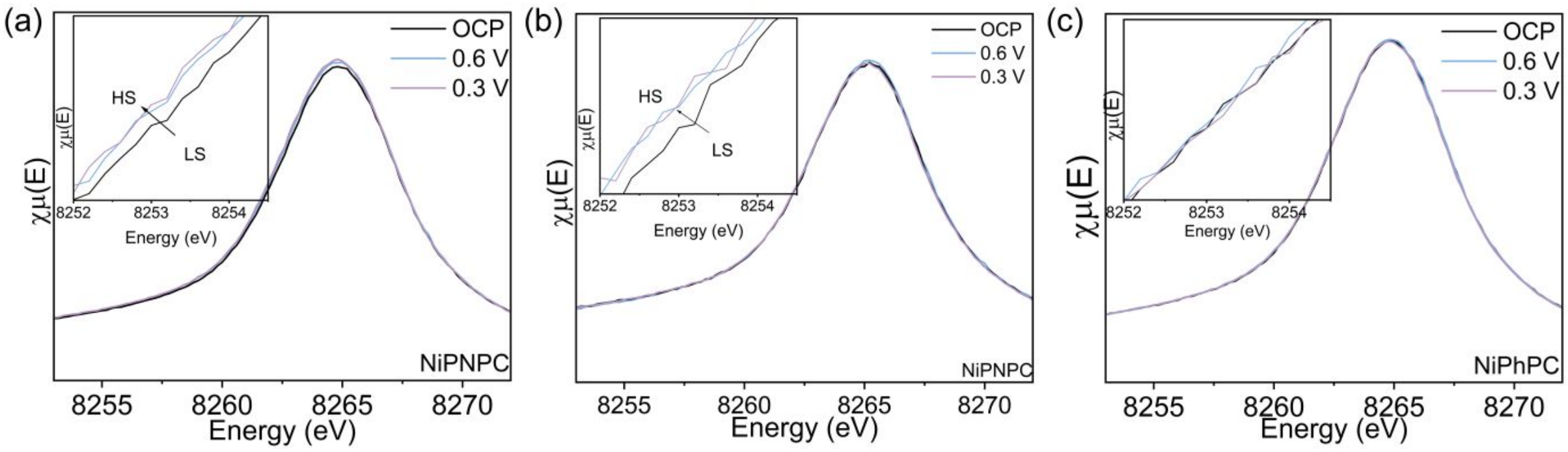


Figure S34. Operando Ni Kβ' XES spectra of repeated NiPNPC in $O_2$ atmosphere, NiPNPC in Ar atmosphere, and NiPhPC in $O_2$ atmosphere.

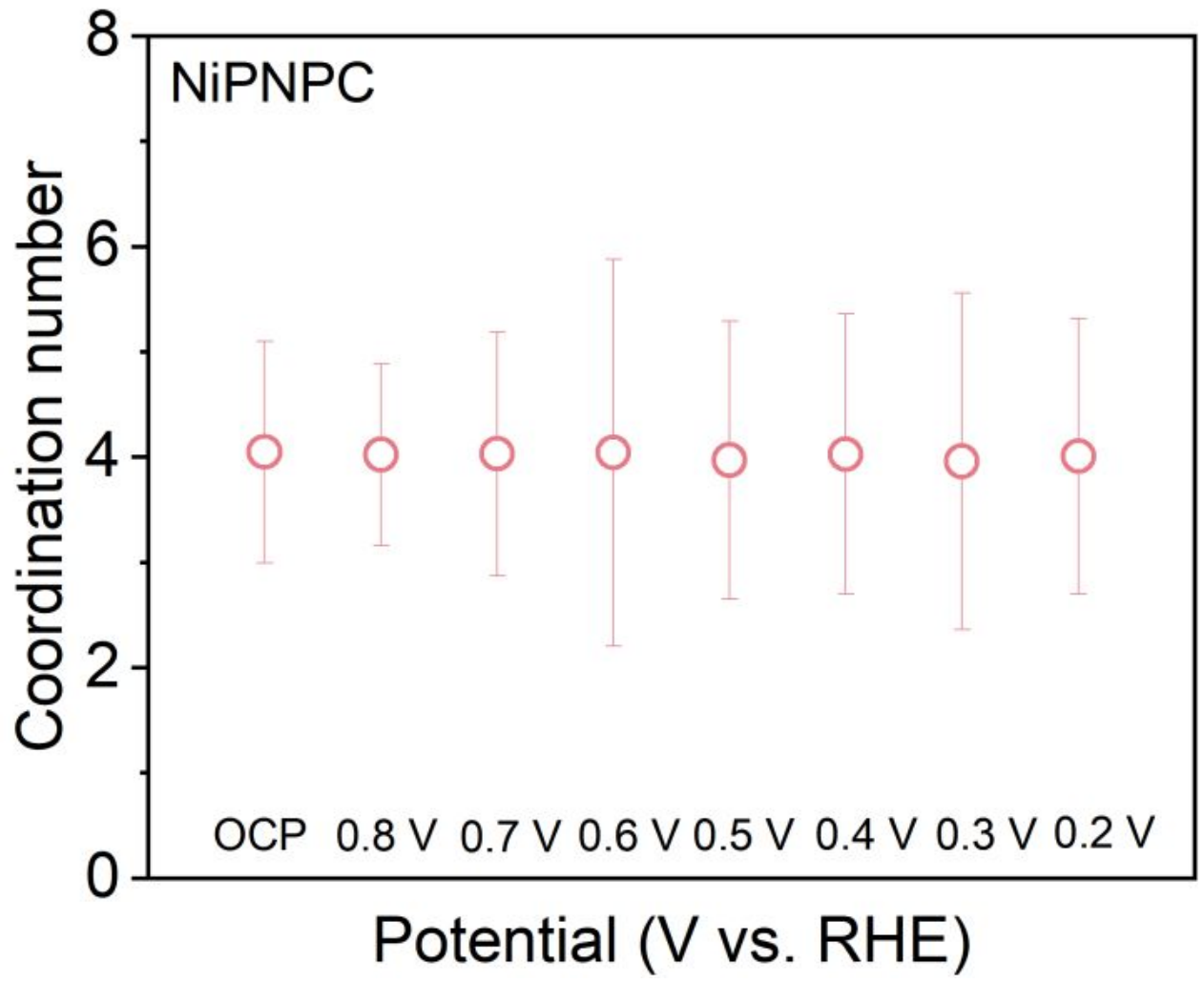


Figure S35. Fitted coordination number of NiPNPC during operando XAFS tests in $O_2$ atmosphere.

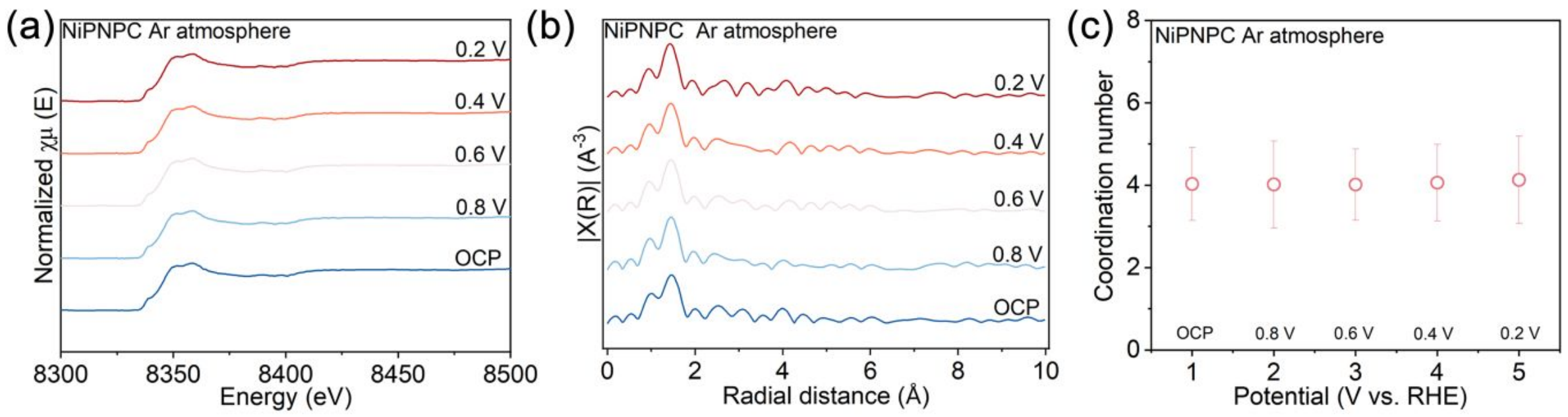


Figure S36. Operando Ni K-edge XANES (a), Fourier-transformed k-space of NiPNPC (b), and fitted coordination number (c) under Ar atmosphere.

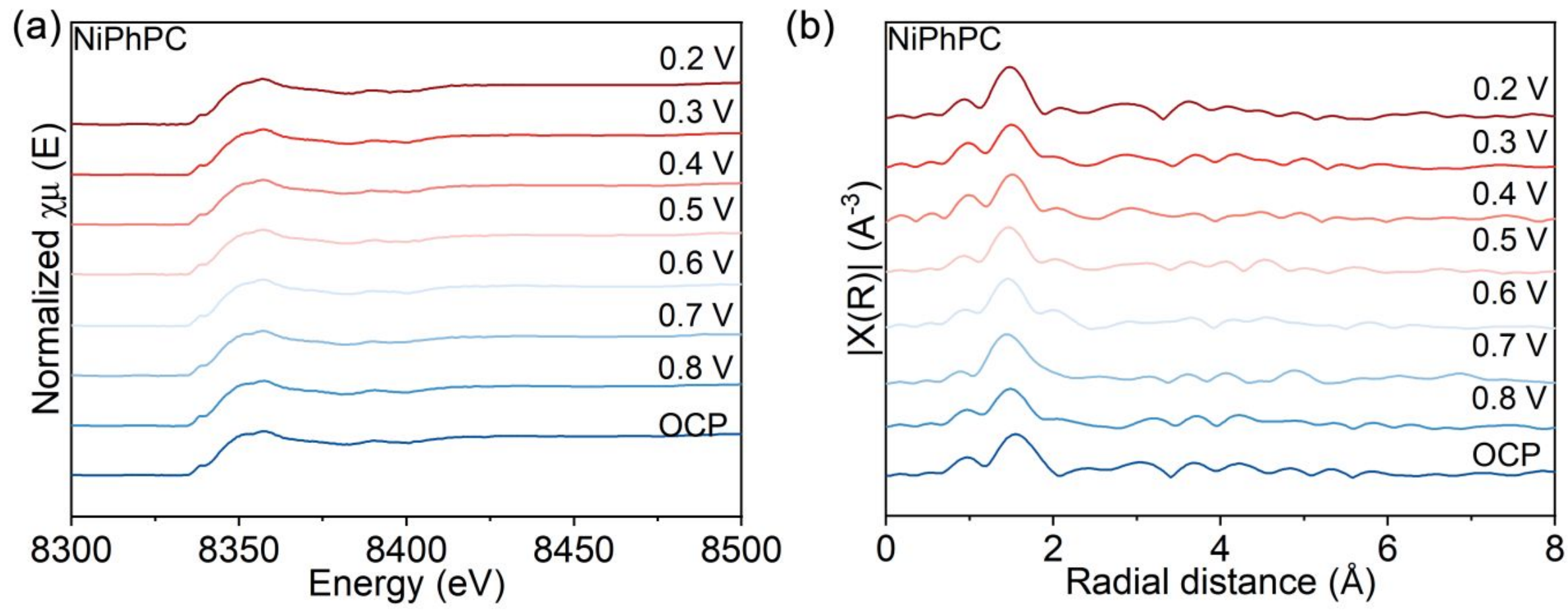


Figure S37. Operando Ni K-edge XANES (a), Fourier-transformed k-space of NiPNPC (b) under $O_2$ atmosphere.

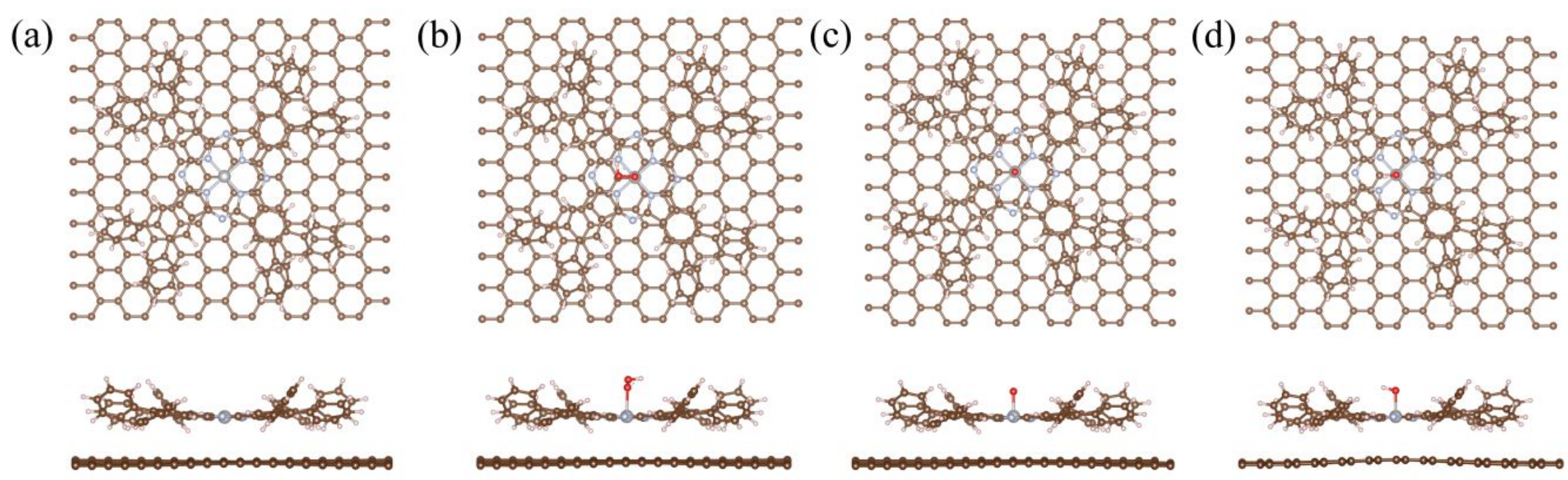


Figure S38. Illustration of optimized structures for NiPhPC during reactions (a) the NiPhPC substrate, (b) *OOH intermediate, (c) *O intermediate, (d) *OH intermediate.

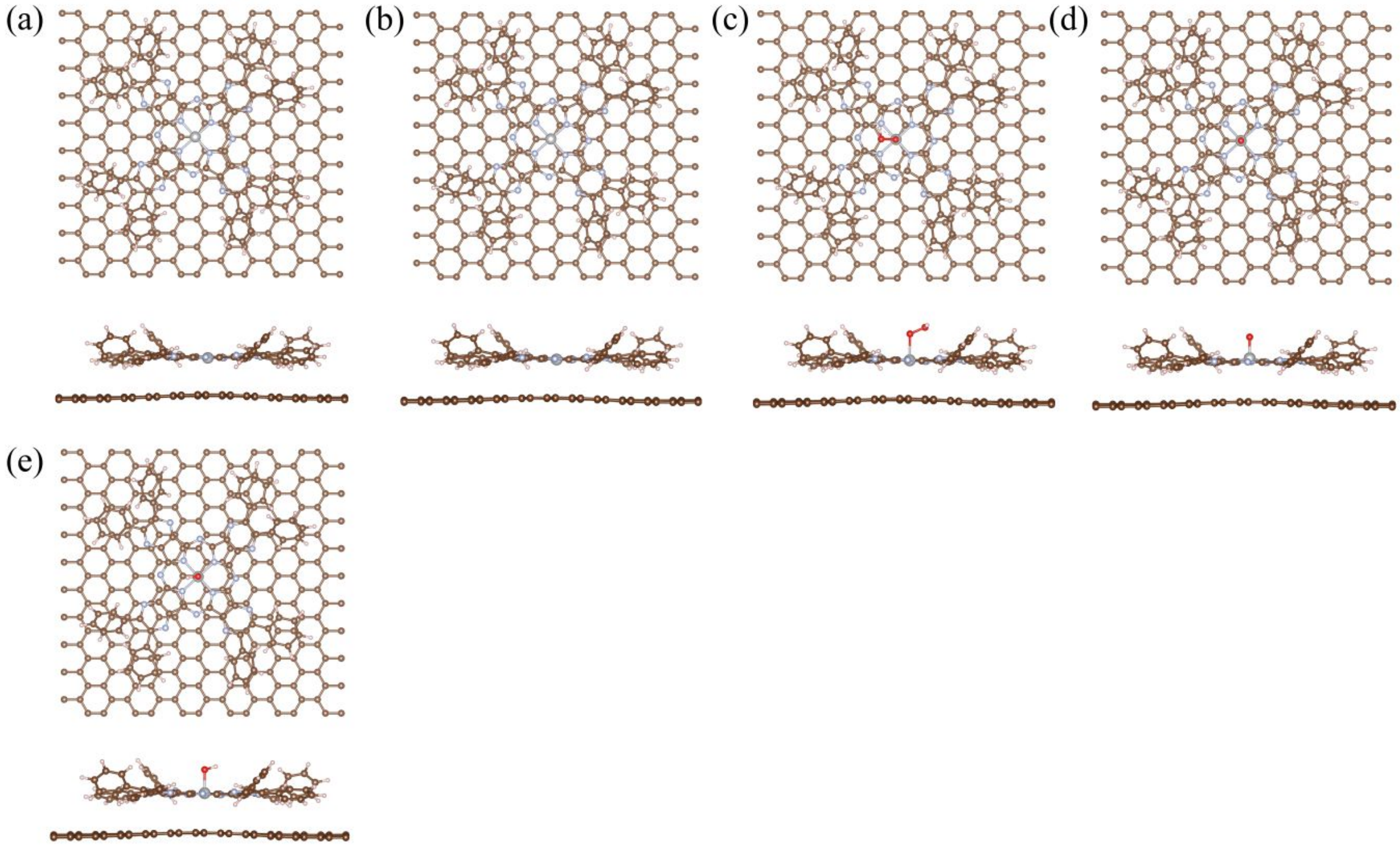


Figure S39. Illustration of optimized structures for NiPNPC during reactions (a) the NiPNPC substrate in a low-spin state, (b) the NiPNPC substrate in a high-spin state, (c) *OOH intermediate, (d) *O intermediate, (e) *OH intermediate.

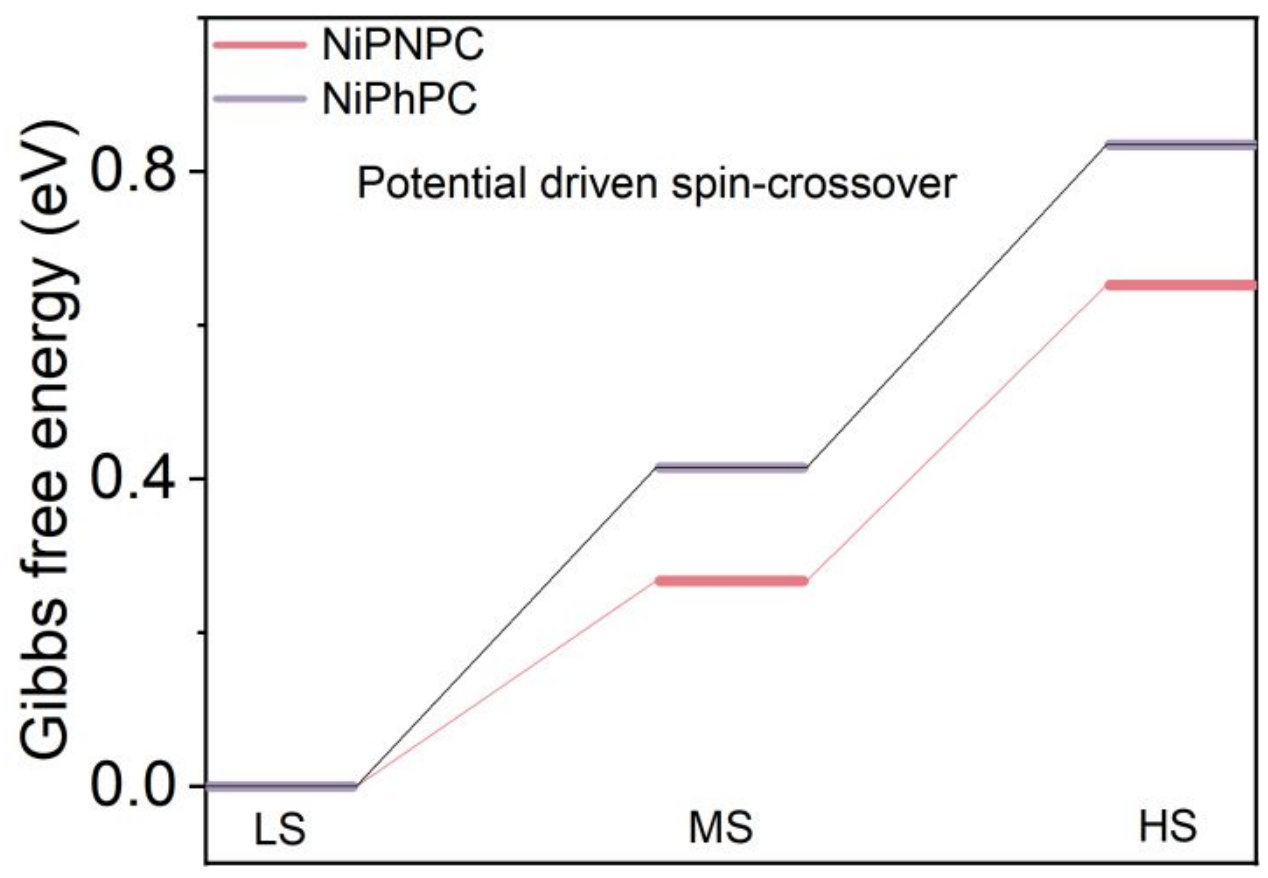


Figure S40. Calculated reaction path of NiPNPC and NiPhPC that is experiencing potential driven spin-crossover process from low-spin state (LS) to middle-state (MS) and finally high-spin state (HS).

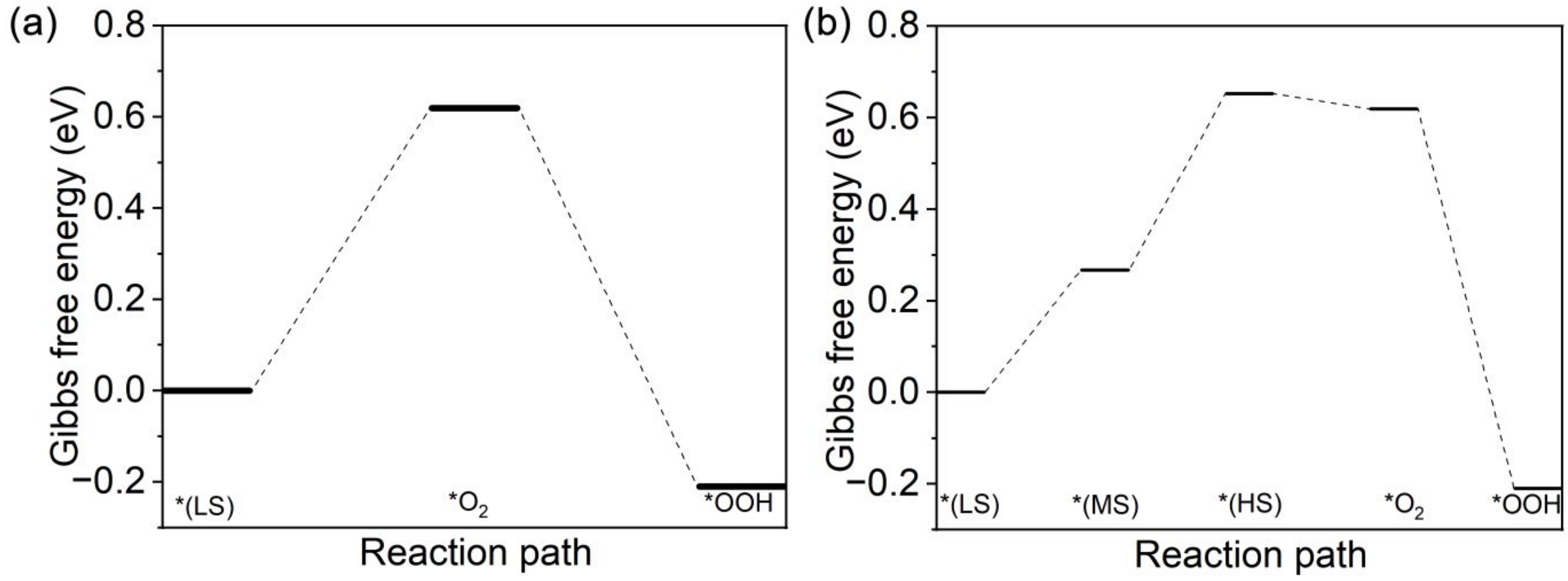


Figure S41. (a) Calculated reaction path of low-spin (LS) state NiPNPC for the formation of *OOH. (b) Calculated reaction path of NiPNPC that is experiencing potential driven spin-crossover to form high-spin state for the formation of *OOH. (The spin state was operated with changing magnetic moment, where low-spin state, middle-state, high-spin state were considered with consideration of the EPR results in Figure 4f and 4g).

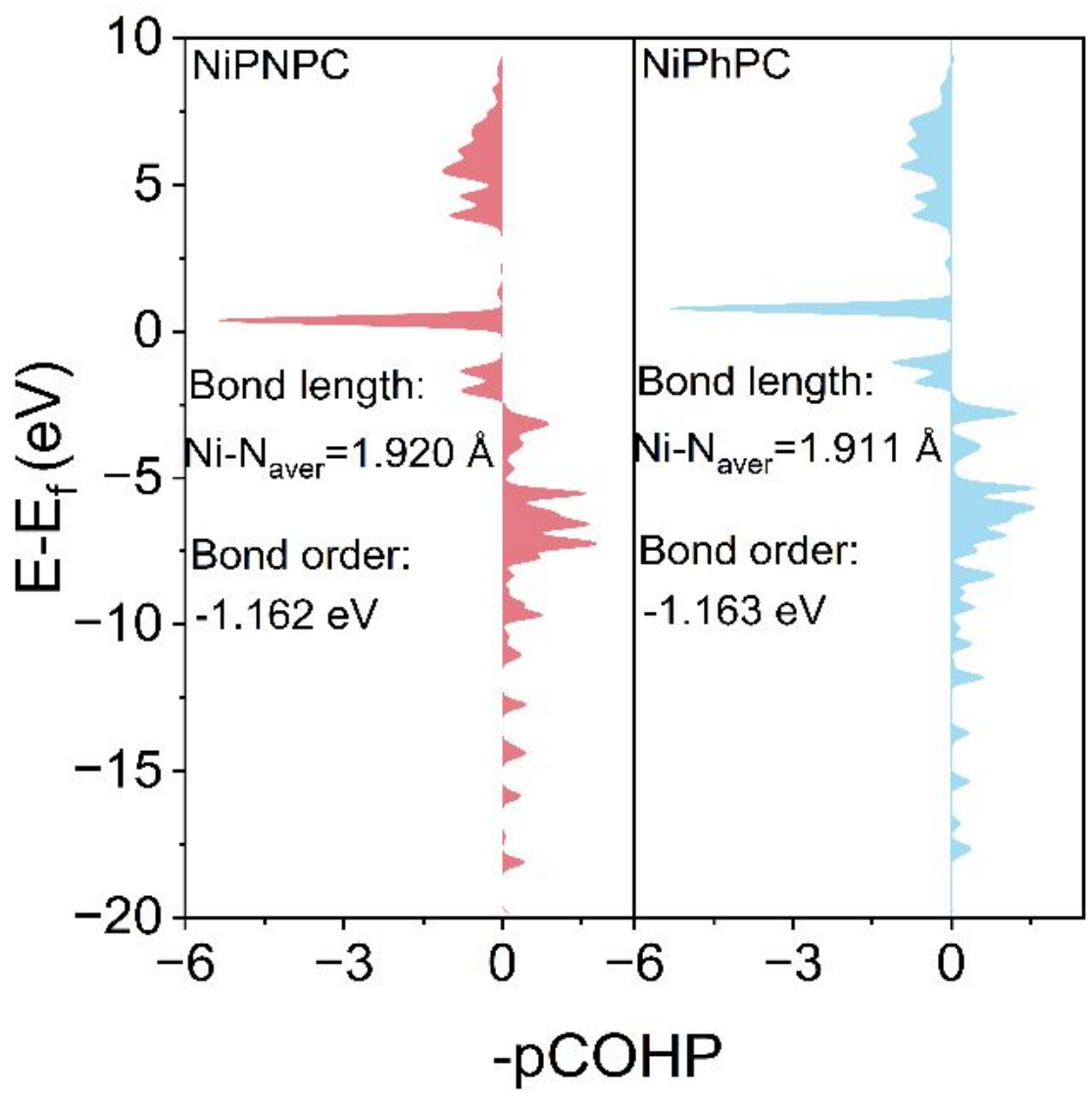


Figure S42. Projected crystal orbital Hamilton population (pCOHP) between Ni and N atoms for NiNPC and NiPhPC.

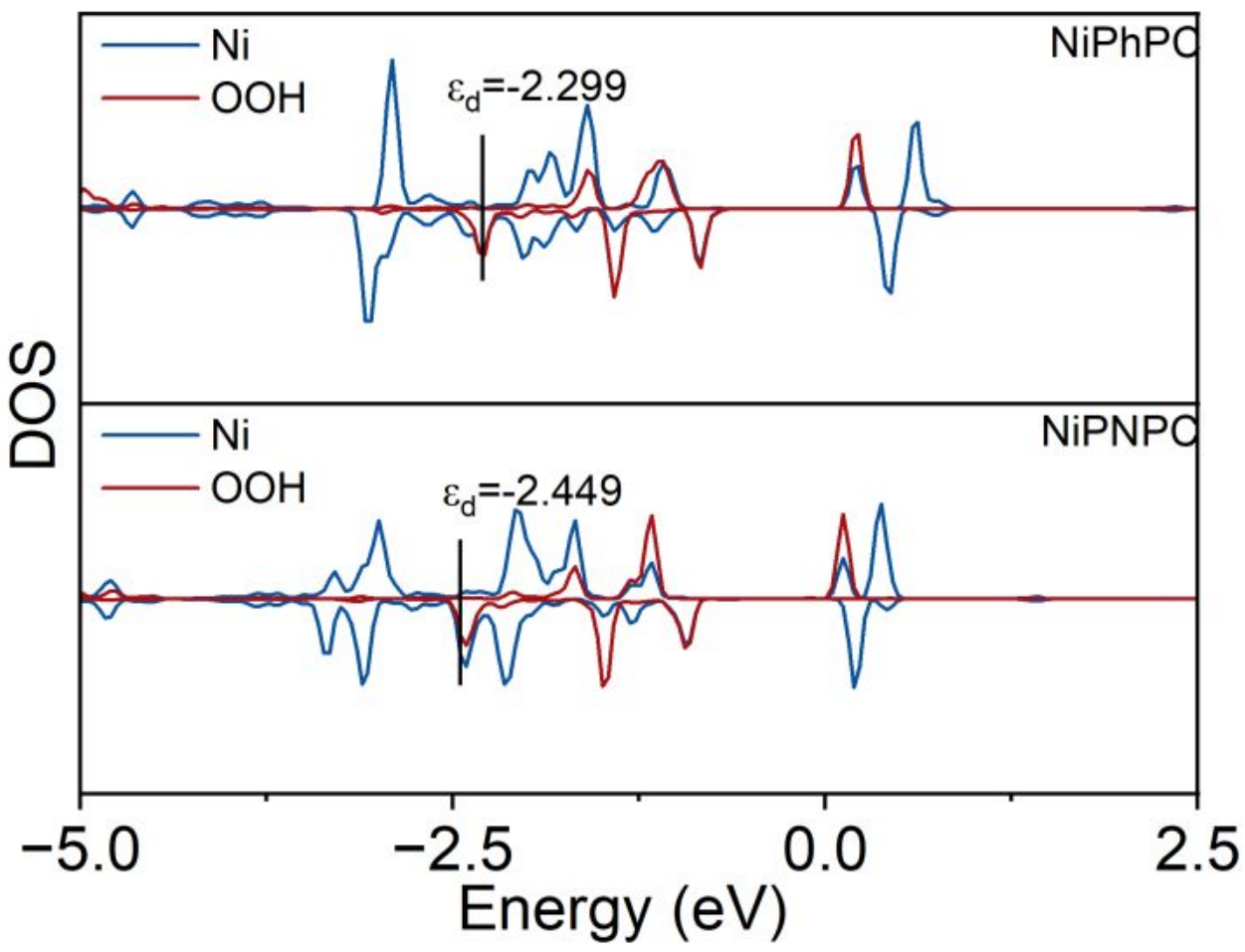


Figure S43. The projected density of states (PDOS) of Ni d orbitals and OOH* intermediates, and the d-band center of Ni for NiPhPC and NiPNPC, respectively.

We have now performed PDOS calculations for NiPNPC and NiPhPC. The results show that the Ni d band center in NiPNPC is downshifted relative to NiPhPC, which is consistent with the Bader charge analysis showing enhanced electron transfer from Ni to the OOH intermediate (Figure S44). This electronic modulation, together with the operando XES results, rationalizes the superior $O_2$ activation and OOH stabilization on NiPNPC. This trend is similar to that observed in our previous research.[3]

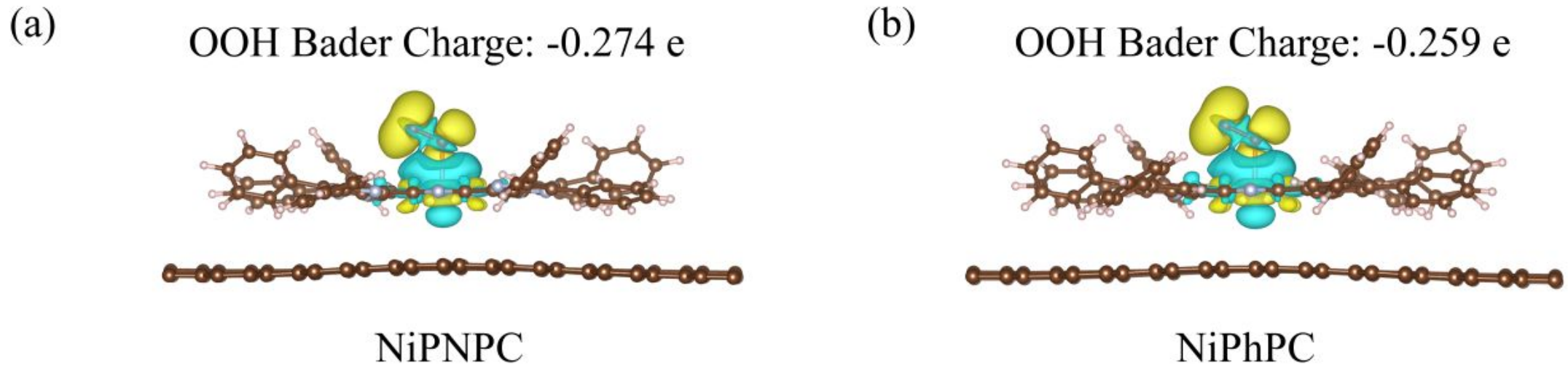


Figure S44. Calculated Bader charge and differential charge density of OOH intermediates.

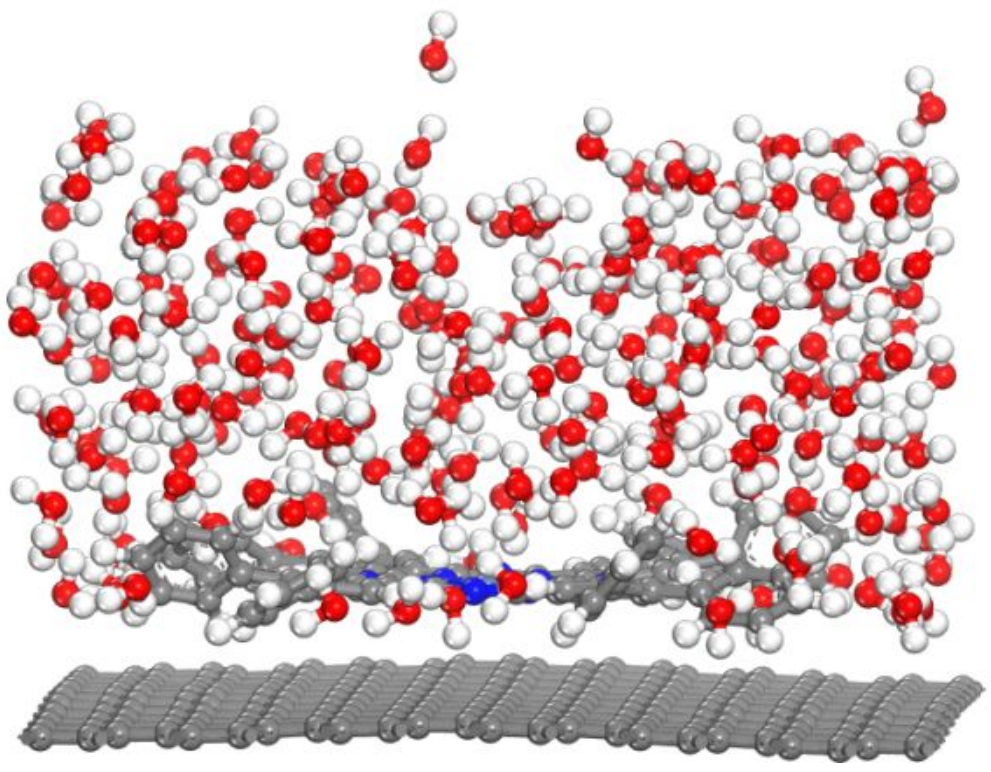

Figure S45. Molecular dynamics simulation results of the interfacial structure for NiPhPC.

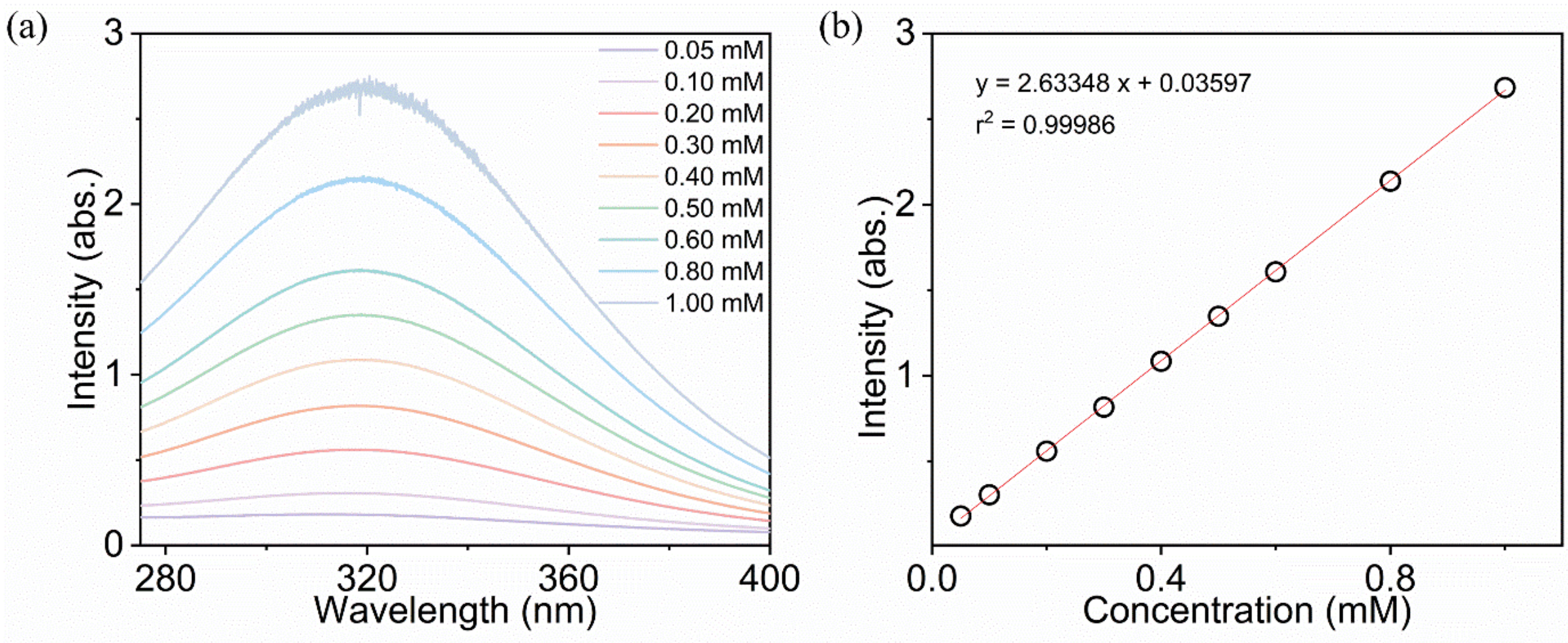


Figure S46. (a) UV-vis absorbance standard spectra of $Ce(SO_4)_2$ in 0.5 M $H_2SO_4$. (b) Converted Standard curve of the $Ce(SO_4)_2$ in 0.5 M $H_2SO_4$.

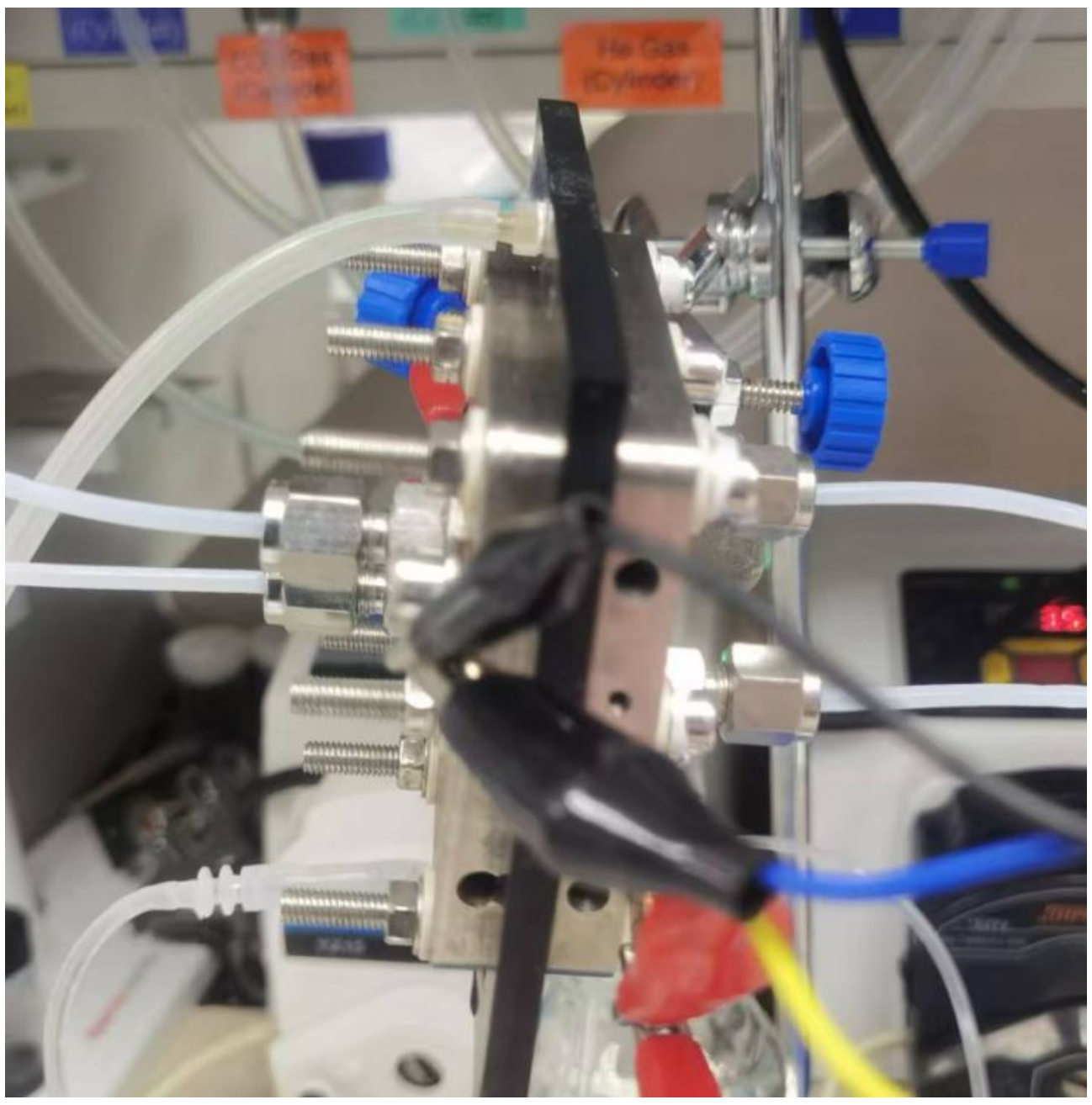

Figure S47. PSE reactor used for $H_2O_2$ production.

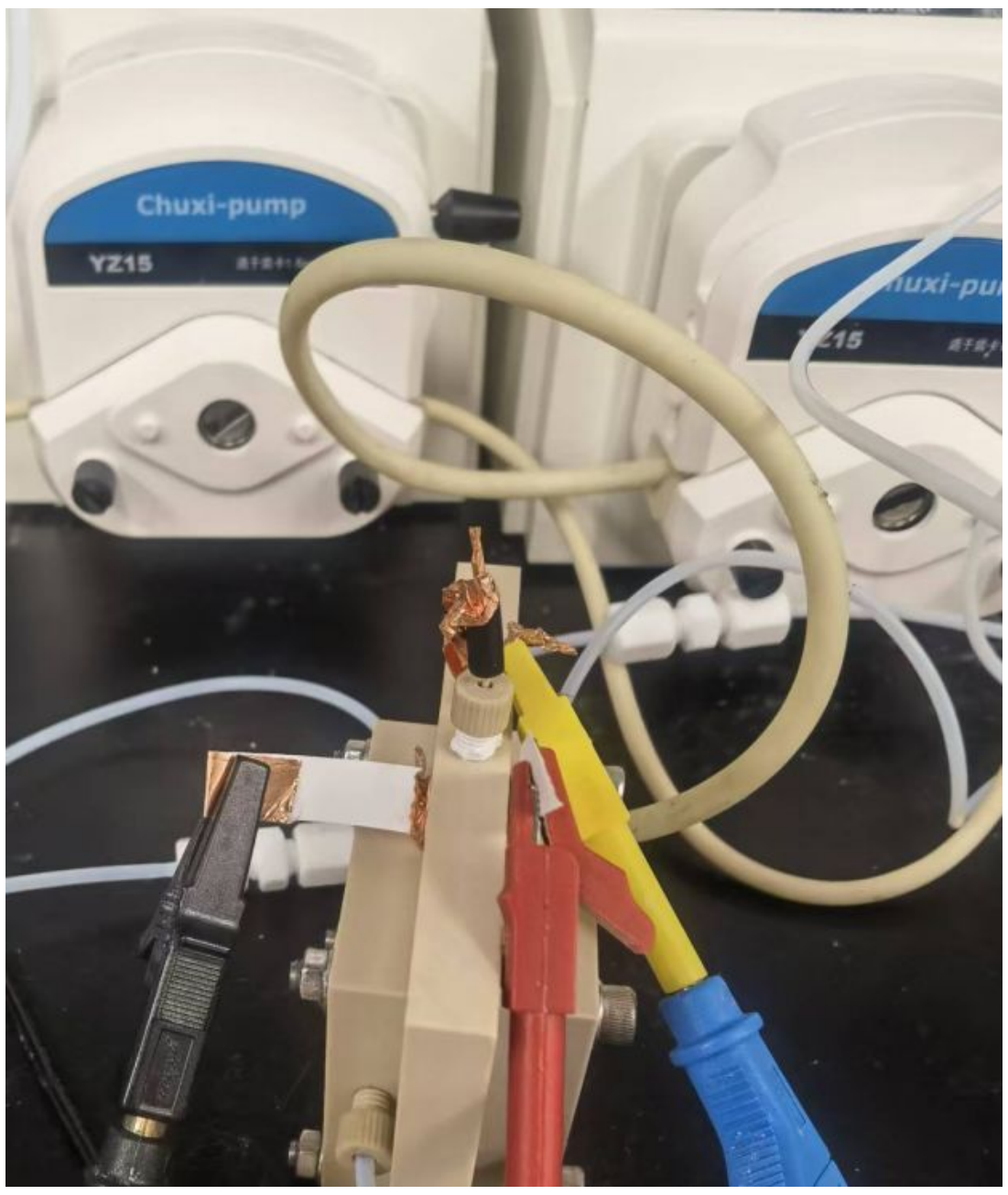


Figure S48. Flow cell used for $H_2O_2$ production.

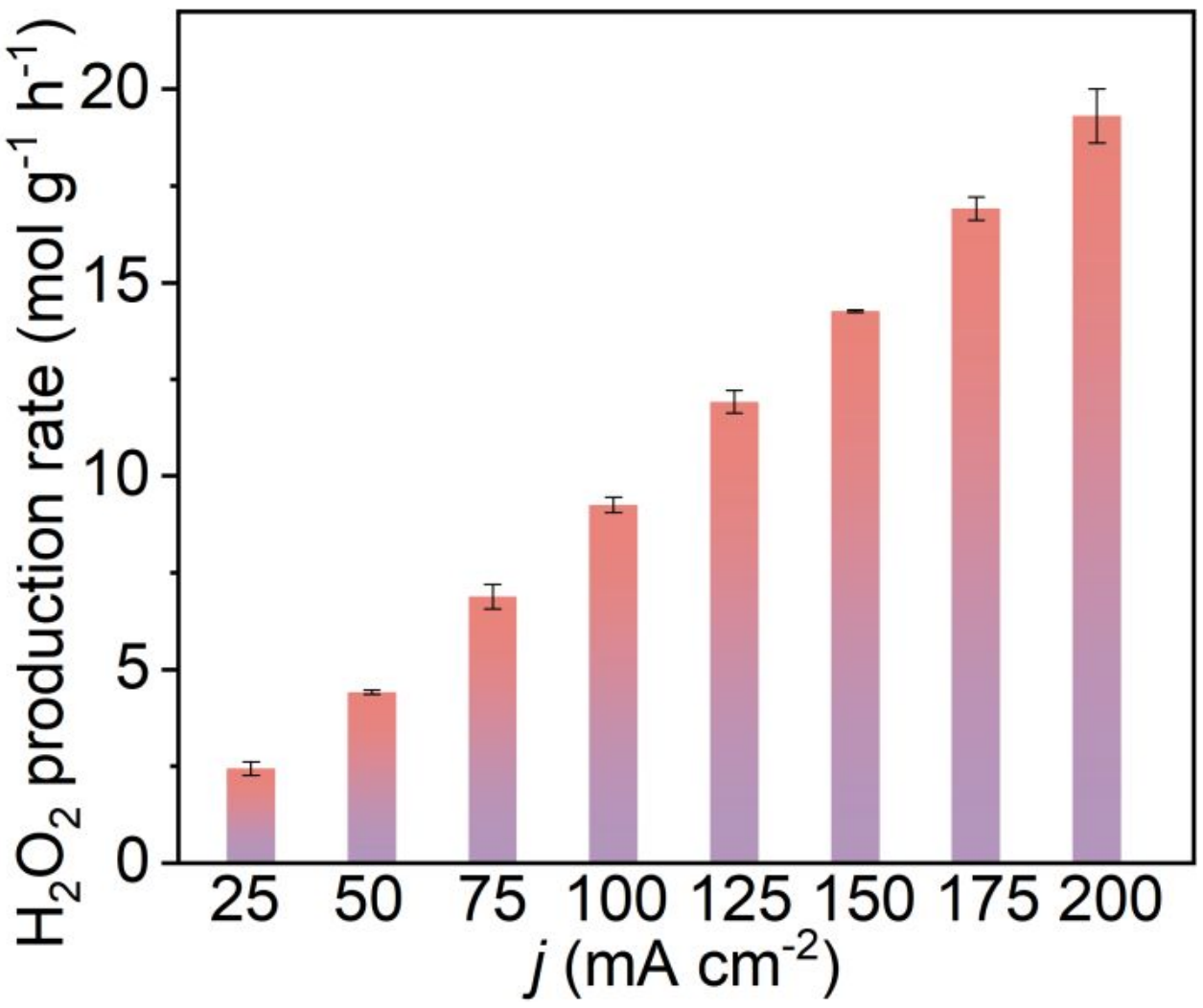


Figure S49. $H_2O_2$ production of NiPNPC under $O_2$ atmosphere in flow cell.

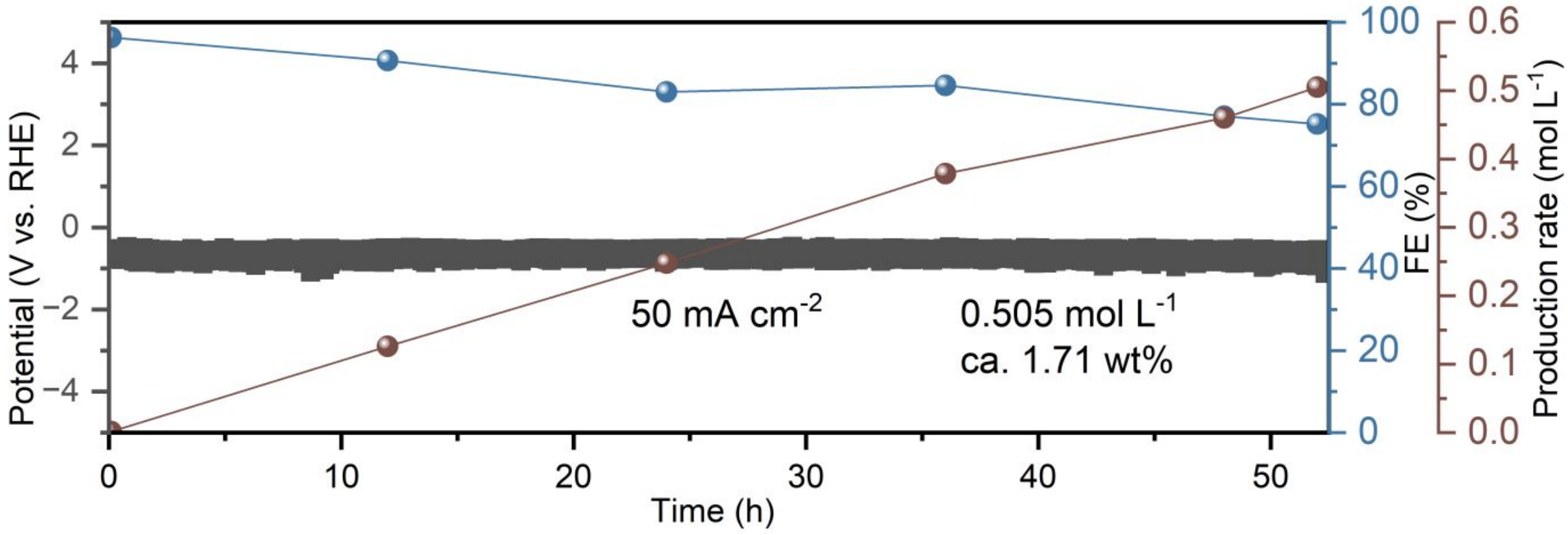


Figure S50. Stability test of NiPNPC in the flow cell.

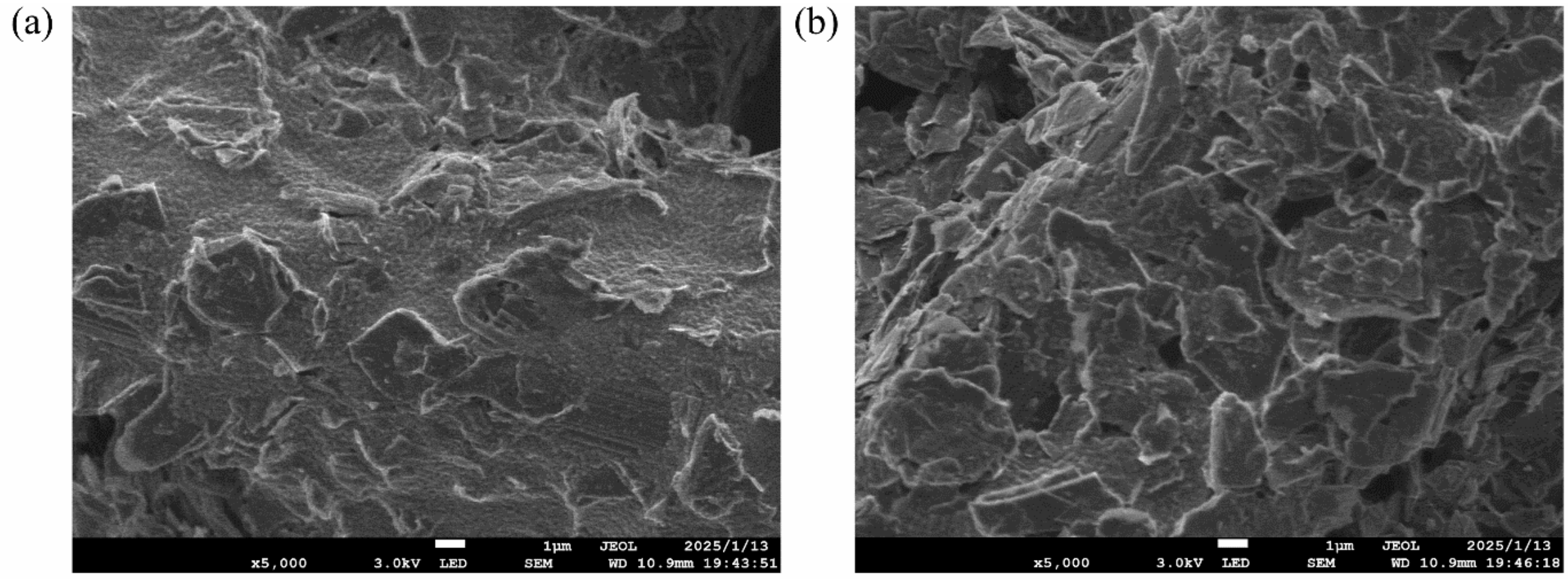


Figure S51. The SEM images of NiPNPC before and after reactions.

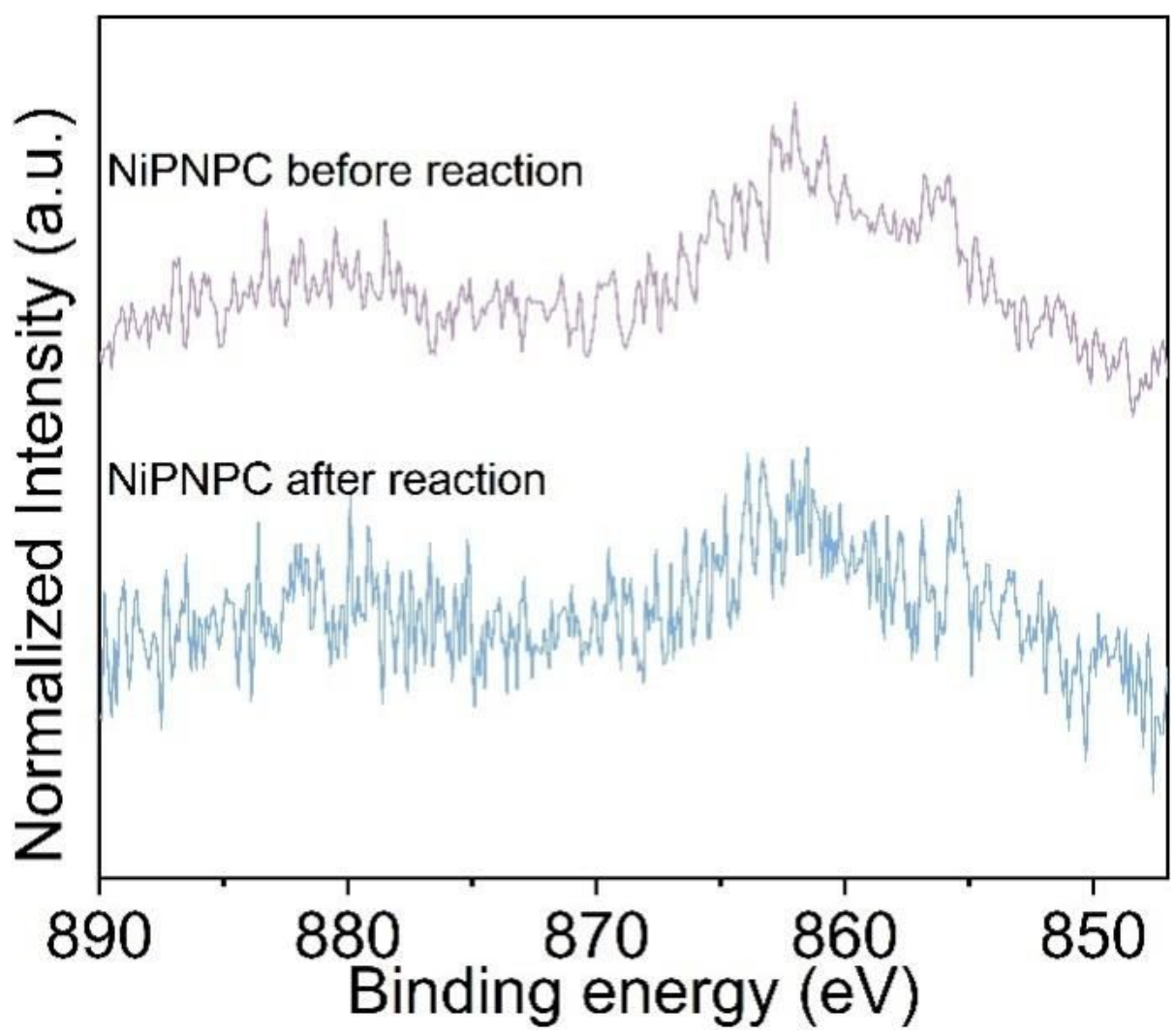


Figure S52. The XPS spectra of NiPNPC before and after reactions.

**Table S1**. Compositional analysis of catalysts with XPS, ICP-MS, and calculated mass loading based on ICP-MS.

| | C (wt%)$_{XPS}$ | N (wt%)$_{XPS}$ | O (wt%)$_{XPS}$ | Ni (wt%)$_{XPS}$ | Ni (wt%)$_{ICP}$ | Loading (g cm$^{-2}$ )$_{Ni\_XPS}$ | Loading (g cm$^{-2}$ )$_{Ni\_ICP}$ |
|---|---|---|---|---|---|---|---|
| NiNPC(CNT) | 89.73 | 4.01 | 3.39 | 2.87 | 2.39 | $3.66\times10^{-6}$ | $3.05\times10^{-6}$ |
| NiPNPC(CNT) | 88.84 | 5.36 | 3.12 | 2.68 | 2.85 | $3.41\times10^{-6}$ | $3.63\times10^{-6}$ |
| NiPhPC(CNT) | 88.94 | 4.95 | 3.38 | 3.30 | 2.88 | $4.20\times10^{-6}$ | $3.67\times10^{-6}$ |
| CNT | 97.58 | 0.52 | 3.90 | - | - | - | - |

**Table S2**. Structural parameters fitted from EXAFS for NiNPC, NiPNPC, and NiPhPC using the NiPC model structure.

| Samples | Shell | CN[a] | R(Å) | $\sigma^2$(×10$^{-3}$Å$^2$) | ΔE (eV) | R-factor (%) |
|---|---|---|---|---|---|---|
| NiPhPC | Ni-N | 4.00±0.67 | 1.89±0.02 | 2.62±2.14 | 3.77±2.01 | 0.78 |
| NiNPC | Ni-N | 3.99±0.69 | 1.86±0.02 | 1.58±2.09 | 0.54±2.78 | 0.92 |
| NiPNPC | Ni-N | 4.00±0.80 | 1.84±0.02 | 4.63±2.41 | 2.00±3.21 | 1.71 |
| NiPhPC(CNT) | Ni-N | 4.01±0.75 | 1.91±0.02 | 3.28±2.13 | 7.17±2.28 | 1.92 |
| NiNPC(CNT) | Ni-N | 4.00±0.69 | 1.88±0.01 | 4.52±1.99 | 5.64±2.12 | 1.43 |
| NiPNPC(CNT) | Ni-N | 4.00±0.81 | 1.90±0.02 | 2.17±2.58 | 5.85±3.21 | 1.25 |

N, coordination number; R, the distance between absorber and backscatter atoms; $\sigma^2$, Debye-Waller factor to account for both thermal and structural disorders; $\Delta E_0$, inner potential correction; R factor (%) indicates the goodness of the fit. $S_0^2$ was determined from Ni foil fitting and fixed to 0.82. Fitting range: 3.0≤k (/Å)≤11.5 and 1≤R(Å)≤3.0.

**Table S3**. Comparison of ORR Performance for FePNPC, CoPNPC, and NiPNPC at 0.5 V vs RHE.

| Catalyst | Without Pyrazine (selectivity) | With Pyrazine (selectivity) | Gap (selectivity) |
|---|---|---|---|
| FePNPC vs FePhPC | 42.37% | 62.65% | +20.28% |
| CoPNPC vs CoPhPC | 80.47% | 89.22% | +8.5% |
| NiPNPC vs NiPhPC | 85.91% | 97.04% | +11.13% |

To underscore the unique advantage of the Ni center in NiPNPC, we synthesized and evaluated analogous Fe- and Co-based phthalocyanines with pyrazine (FePNPC, CoPNPC) and phenyl (FePhPC, CoPhPC) substituents for comparison. As shown in Figures 3g–3i, their catalytic behavior differs markedly. FePNPC and FePhPC both exhibited an intrinsic tendency of $4e^-$ ORR pathway, consistent with the established behavior of Fe–$N_4$ centers, while both CoPNPC and CoPhPC promoted a moderate $2e^-$ pathway, in line with the well-known tendency of Co–$N_4$ moieties. Notably, while pyrazine substitution improved the $H_2O_2$ selectivity for all three metals to varying degrees, NiPNPC achieved the highest absolute selectivity of 97.04%, substantially higher than CoPNPC (89.22%) and FePNPC (62.65%). This substantially higher absolute selectivity for Ni demonstrates that pyrazine exerts a synergistic effect on Ni that is stronger than on Co or Fe. In NiPNPC, pyrazine facilitates both the potential-driven spin crossover (evidenced by operando XES, Figure 5a) and the reorganization of the interfacial hydrogen-bond network (Figure 4f). For CoPNPC and FePNPC, the improvements were limited, consistent with their intrinsically different electronic structures. These comparative results position NiPNPC as the optimal catalyst within this series for highly selective $H_2O_2$ production via the $2e^-$ ORR.

**Table S4**. Fitting results of O-H stretching vibrational for NiPhPC, NiNPC, NiPNPC, CNT, and ZnPNPC.

| NiPhPC | 4H·$H_2O$ | 2H·$H_2O$ | free $H_2O$ |
|---|---|---|---|
| 0.9 V vs. RHE | 264016.592±14945.921 | 130936.111±39377.978 | 386058.490±41116.431 |
| 0.8 V vs. RHE | 268552.570±31451.180 | 270773.791±71323.868 | 202085.892±48314.382 |
| 0.7 V vs. RHE | 269814.933±19844.659 | 271503.196±48937.970 | 176129.857±34060.713 |
| 0.6 V vs. RHE | 283484.472±22289.017 | 184850.639±55148.879 | 231999.917±41353.217 |
| 0.5 V vs. RHE | 369251.886±25260.044 | 197713.657±52380.263 | 292448.861±38274.279 |
| 0.4 V vs. RHE | 398197.537±30323.940 | 191065.909±53667.871 | 253147.306±36035.940 |
| 0.3 V vs. RHE | 378230.832±24967.245 | 142627.540±42154.480 | 310992.277±33697.816 |
| 0.2 V vs. RHE | 428076.773±24191.734 | 102107.625±31403.057 | 403301.093±40690.596 |
| NiNPC | 4H·$H_2O$ | 2H·$H_2O$ | free $H_2O$ |
| 0.9 V vs. RHE | 409046.745±36667.636 | 808760.639±69876.525 | 161751.293±37127.928 |
| 0.8 V vs. RHE | 481658.407±37947.474 | 772622.340±65281.717 | 175267.616±31947.882 |
| 0.7 V vs. RHE | 543379.866±36050.255 | 761920.334±60047.820 | 186797.266±28901.647 |
| 0.6 V vs. RHE | 565345.649±34305.289 | 790005.254±56115.201 | 191286.193±26397.764 |
| 0.5 V vs. RHE | 569313.741±36297.729 | 799343.830±62007.367 | 208912.791±30565.869 |
| 0.4 V vs. RHE | 494226.853±41250.744 | 960978.891±69732.173 | 153960.371±31987.858 |
| 0.3 V vs. RHE | 586382.135±38597.269 | 865073.048±63256.669 | 211992.225±30636.035 |
| 0.2 V vs. RHE | 713230.743±36762.603 | 665894.748±72310.229 | 310209.703±43224.355 |
| NiPNPC | 4H·$H_2O$ | 2H·$H_2O$ | free $H_2O$ |
| 0.9 V vs. RHE | 249008.551±19211.372 | 563459.239±36136.830 | 87690.339±18857.800 |
| 0.8 V vs. RHE | 260960.094±20259.458 | 510041.211±37711.650 | 96969.608±20013.021 |
| 0.7 V vs. RHE | 243808.192±21297.990 | 518658.994±40128.762 | 92175.394±21123.799 |
| 0.6 V vs. RHE | 263342.652±19797.952 | 475522.815±38856.293 | 121757.217±21944.706 |
| 0.5 V vs. RHE | 271950.698±21724.204 | 537784.895±38856.293 | 118473.286±23512.073 |
| 0.4 V vs. RHE | 271576.869±23947.603 | 444367.405±48710.332 | 142509.224±28688.203 |
| 0.3 V vs. RHE | 282278.944±27081.762 | 384447.863±74706.895 | 246740.441±54111.960 |
| 0.2 V vs. RHE | 402344.243±26980.486 | 364371.474±69956.209 | 338347.726±51696.661 |
| CNT | 4H·$H_2O$ | 2H·$H_2O$ | free $H_2O$ |
| 0.9 V vs. RHE | 189452.003±16688.721 | 506088.698±33554.350 | 65775.105±18022.226 |

| | | | |
|---|---|---|---|
| 0.8 V vs. RHE | 174662.971±15253.611 | 377288.302±29593.555 | 58533.849±15916.351 |
| 0.7 V vs. RHE | 163042.077±13049.174 | 341904.612±26497.556 | 72186.860±15114.996 |
| 0.6 V vs. RHE | 135817.477±13005.902 | 337056.804±26462.517 | 54760.858±14492.280 |
| 0.5 V vs. RHE | 106830.218±11527.238 | 314052.125±23521.690 | 44197.273±13114.384 |
| 0.4 V vs. RHE | 147434.297±13030.888 | 206185.594±27570.199 | 77478.872±16783.933 |
| 0.3 V vs. RHE | 148432.719±13167.094 | 153031.929±30837.316 | 82045.093±20691.142 |
| 0.2 V vs. RHE | 138076.516±10376.678 | 131037.308±23998.791 | 90623.006±16521.688 |
| ZnPNPC | 4H·$H_2O$ | 2H·$H_2O$ | free $H_2O$ |
| 0.9 V vs. RHE | 795696.105±49996.432 | 1325741.465±74712.889 | 195179.044±29304.098 |
| 0.8 V vs. RHE | 674754.233±51448.669 | 1412041.020±80825.231 | 169555.081±33118.265 |
| 0.7 V vs. RHE | 602652.819±47000.652 | 916365.459 ±73016.190 | 191735.137±35349.444 |
| 0.6 V vs. RHE | 711636.367±41666.209 | 1431640.176±65954.028 | 177386.223±27273.842 |
| 0.5 V vs. RHE | 706401.773±52482.410 | 879129.286±104756.385 | 346789.328±66802.362 |
| 0.4 V vs. RHE | 892498.362±53909.102 | 795270.456±103571.071 | 379346.461±67206.510 |
| 0.3 V vs. RHE | 952522.212±55874.833 | 866038.786±99785.732 | 357819.209±60819.404 |
| 0.2 V vs. RHE | 943090.895±47735.704 | 959999.510±84389.167 | 336985.396±45108.024 |

**Table S5**. Summary of the parameters for tests in porous state electrolyte cell.

| Current density (mA cm$^{-2}$) | FE | $H_2O_2$ concentration (mmol L$^{-1}$) | Production rate (mmol cm$^{-2}$ h$^{-1}$) | Total charge passed (C) | Electrolyte pH |
|---|---|---|---|---|---|
| 10 | 0.954±0.015 | 5.934±0.095 | 0.177±0.004 | 9.0 | 8.78±0.04 |
| 25 | 0.946±0.010 | 14.709±0.153 | 0.440±0.006 | 22.5 | 11.01±0.03 |
| 50 | 0.932±0.002 | 28.966±0.839 | 0.866±0.002 | 45.0 | 11.45±0.03 |
| 75 | 0.926±0.009 | 43.173±0.503 | 1.290±0.008 | 67.5 | 11.27±0.01 |
| 100 | 0.912±0.025 | 56.727±0.625 | 1.695±0.021 | 90.0 | 10.94±0.02 |
| 125 | 0.904±0.013 | 70.281±0.854 | 2.103±0.024 | 112.5 | 10.69±0.01 |
| 150 | 0.878±0.005 | 81.878±0.451 | 2.447±0.019 | 135.0 | 10.11±0.03 |
| 175 | 0.856±0.012 | 93.122±1.260 | 2.783±0.053 | 157.5 | 9.09±0.01 |
| 200 | 0.848±0.008 | 105.422±0.850 | 3.150±0.042 | 180.0 | 9.00±0.01 |

**Table S6**. Summary of the parameters for tests in flow cell.

| Current density (mA $cm^{-2}$) | FE | $H_2O_2$ concentration (mmol $L^{-1}$) | Production rate (mol $g^{-1}$ $h^{-1}$) | Total charge passed (C) | Electrolyte pH |
|---|---|---|---|---|---|
| 25 | 0.967±0.001 | 3.757±0.038 | 2.251±0.023 | 15.0 | 13.67±0.01 |
| 50 | 0.963±0.018 | 7.482±0.140 | 4.489±0.084 | 30.0 | 13.69±0.03 |
| 75 | 0.950±0.014 | 11.084±0.164 | 6.650±0.098 | 45.0 | 13.70±0.03 |
| 100 | 0.942±0.019 | 14.648±0.299 | 8.789±0.179 | 60.0 | 13.69±0.04 |
| 125 | 0.938±0.030 | 18.226±0.575 | 10.935±0.345 | 75.0 | 13.71±0.06 |
| 150 | 0.889±0.014 | 20.739±0.330 | 12.444±0.198 | 90.0 | 13.72±0.01 |
| 175 | 0.868±0.017 | 23.618±0.466 | 14.171±0.279 | 105.0 | 13.72±0.01 |
| 200 | 0.857±0.031 | 26.640±0.956 | 15.984±0.573 | 120.0 | 13.72±0.03 |

**Table S7**. Summary of the parameters for biomass conversion in flow cell.

| | Current density (mA $cm^{-2}$) | FE (%) | $H_2O_2$ concentration (mmol $L^{-1}$) | Production rate (mol $g^{-1}$ $h^{-1}$ ) | Total charge passed (C) | Electrolyte pH |
|---|---|---|---|---|---|---|
| Glycerol | 25 | 0.838±0.051 | 3.258±0.200 | 1.955±0.120 | 15.0 | 13.68±0.02 |
| | 50 | 0.875±0.032 | 6.800±0.251 | 4.080±0.151 | 30.0 | 13.69±0.02 |
| | 75 | 0.865±0.031 | 10.081±0.363 | 6.049±0.218 | 45.0 | 13.68±0.01 |
| | 100 | 0.872±0.014 | 13.555±0.224 | 8.133±0.135 | 60.0 | 13.68±0.02 |
| | 150 | 0.828±0.076 | 19.303±0.177 | 11.582±0.106 | 90.0 | 13.69±0.02 |
| Erythritol | 75 | 0.756±0.027 | 8.808±0.315 | 5.285±0.189 | 45.0 | 13.64±0.01 |
| Xylitol | 75 | 0.869±0.021 | 10.136±0.250 | 6.081±0.150 | 45.0 | 13.68±0.02 |
| Glucose | 75 | 0.678±0.015 | 7.902±0.311 | 4.741±0.186 | 45.0 | 13.69±0.02 |

**Table S8**. The comparison of electrolyte in the middle channel of PSE before and after stability test.

| | Conductivity (μS cm⁻¹) | pH | Sulfate concentration(ion chromatigraphy) (mg L⁻¹) | ICP(Ni leaching) (ppb) | TOC (mg L⁻¹) | Total charge (C cm⁻²) | $O_2$ flow rate (sccm) |
|---|---|---|---|---|---|---|---|
| PSE | | | | | | | |
| Initial | 1.21 | 6.60 | - | - | - | - | 40 |
| Product | 79.7 | 3.86 | 28 | 1.216 | 8.231 | 36,000[a] | 40 |

a. After reaction for 100h.

Purity analysis of the outlet solution shows that the $H_2O_2$ collected from the water-fed PSE stream has a conductivity of 79.7 μS $cm^{-1}$ and a pH of 3.86, this is due to that $H_2O_2$ is a weak acid, and the proton may across to the solution. The sulfate concentration was 28 mg $L^{-1}$, indicating also minor permeation of sulfate from sulfate electrolyte. While the total organic carbon (TOC) was 8.231 mg $L^{-1}$, likely arising from the Dowex resin or membrane components. Ni leaching was also detected with a content of 1.216 ppb, confirming relatively slow leaching of the NiPNPC catalyst. These results indicate that the PSE system produces $H_2O_2$ with acceptable purity for downstream applications, though further optimization of the membrane and resin materials could reduce sulfate and TOC levels.

**Table S9**. The comparison of electrolyte of flow cell before and after stability test.

| | pH | Total charge (C $cm^{-2}$) | $O_2$ flow rate (sccm) |
|---|---|---|---|
| Flow cell | | | |
| Initial | 13.69 | - | 25 |
| Product | 13.91 | 9,360[a] | 25 |

a. After reaction for 52h.

**Reference**:


(1) Hordiyenko, O. V.; Rudenko, I. V.; Zamkova, I. A.; Denisenko, O. V.; Biitseva, A. V.; Arrault, A.; Tolmachev, A. A. Facile Synthesis of Hydrazine Derivatives of 5H-Pyrrolo[3,4-b]pyrazine and 1H-Pyrrolo[3,4-b]quinoxaline. *Synthesis* **2013**, *45* (24), 3375-3382.
(2) Tao, Y.; Li, J.-R.; Chang, Z.; Bu, X.-H. ZnII and HgII Complexes with 2,3-Substituted-5,6-di(1H-tetrazol-5-yl)pyrazine Ligands: Roles of Substituting Groups and Synthetic Conditions on the Formation of Complexes. *Crystal Growth & Design* **2010**, *10* (2), 564-574.
(3) Sun, L.; Jin, X.; Su, T.; Fisher, A. C.; Wang, X. Conjugated Nickel Phthalocyanine Derivatives for Heterogeneous Electrocatalytic $H_2O_2$ Synthesis. *Advanced Materials* **2024**, *36* (17), 2306336.
(4) Liu, Y.; Li, L.; Li, X.; Xu, Y.; Wu, D.; Sakthivel, T.; Guo, Z.; Zhao, X.; Dai, Z. Asymmetric tacticity navigates the localized metal spin state for sustainable alkaline/sea water oxidation. *Science Advances 11* (22), eads0861.
(5) *Gaussian 16 Rev. C.01*; Gaussian, Wallingford, CT: Gaussian, Wallingford, CT, 2016.
(6) Becke, A. D. Density-functional thermochemistry. III. The role of exact exchange. *The Journal of Chemical Physics* **1993**, *98* (7), 5648-5652.
(7) Krishnan, R.; Binkley, J. S.; Seeger, R.; Pople, J. A. Self-consistent molecular orbital methods. XX. A basis set for correlated wave functions. *The Journal of Chemical Physics* **1980**, *72* (1), 650-654.
(8) Hay, P. J.; Wadt, W. R. Ab initio effective core potentials for molecular calculations. Potentials for the transition metal atoms Sc to Hg. *The Journal of Chemical Physics* **1985**, *82* (1), 270-283.
(9) Hay, P. J.; Wadt, W. R. Ab initio effective core potentials for molecular calculations. Potentials for K to Au including the outermost core orbitals. *The Journal of Chemical Physics* **1985**, *82* (1), 299-310.
(10) Grimme, S.; Antony, J.; Ehrlich, S.; Krieg, H. A consistent and accurate ab initio parametrization of density functional dispersion correction (DFT-D) for the 94 elements H-Pu. *The Journal of Chemical Physics* **2010**, *132* (15), 154104.
(11) Grimme, S.; Ehrlich, S.; Goerigk, L. Effect of the damping function in dispersion corrected density functional theory. *Journal of Computational Chemistry* **2011**, *32* (7), 1456-1465.
(12) Humphrey, W.; Dalke, A.; Schulten, K. VMD: Visual molecular dynamics. *Journal of Molecular Graphics* **1996**, *14* (1), 33-38.
(13) Lu, T.; Chen, F. Multiwfn: A multifunctional wavefunction analyzer. *Journal of Computational Chemistry* **2012**, *33* (5), 580-592.
(14) Kresse, G.; Joubert, D. From ultrasoft pseudopotentials to the projector augmented-wave method. *Physical Review B* **1999**, *59* (3), 1758-1775.
(15) Perdew, J. P.; Burke, K.; Ernzerhof, M. Generalized Gradient Approximation Made Simple. *Physical Review Letters* **1996**, *77* (18), 3865-3868.
(16) Nørskov, J. K.; Rossmeisl, J.; Logadottir, A.; Lindqvist, L.; Kitchin, J. R.; Bligaard, T.; Jónsson, H. Origin of the Overpotential for Oxygen Reduction at a Fuel-Cell Cathode. *The Journal of Physical Chemistry B* **2004**, *108* (46), 17886-17892.
(17) Wang, V.; Xu, N.; Liu, J.-C.; Tang, G.; Geng, W.-T. VASPKIT: A user-friendly interface facilitating high-throughput computing and analysis using VASP code. *Computer Physics Communications* **2021**, *267*, 108033.
(18) Tung, C.-W.; Zhang, W.; Lai, T. Y.; Wang, J.; Chu, Y.-C.; Wang, G.-B.; Hsu, C.-S.; Liao, Y.-F.; Hiraoka, N.; Ishii, H. Spin crossover-driven diiron electrocatalyst boosts sustainable water oxidation. *Nature Sustainability* **2025**, *8* (7), 793-805.
(19) van Langevelde, P. H.; Ligthart, N. E. G.; van Duren, P. G. J.; Vermaas, D. A.; Hetterscheid, D. G. H. H2O2 Synthesis with Molecular Electrocatalysts Enables Substantial Current Densities in a Flow Cell Using Gas Diffusion Electrodes. *ACS Electrochemistry* **2025**, *1* (11), 2326-2337.
(20) Li, X.; Tang, S.; Dou, S.; Fan, H. J.; Choksi, T. S.; Wang, X. Molecule confined isolated metal sites enable the electrocatalytic synthesis of hydrogen peroxide. *Advanced Materials* **2022**, *34* (25), 2104891.

(21) Sun, L.; Zhai, Y.; Yu, D.; Chen, Y.; Liang, Z.; Qiao, Z. A.; Wang, X. Dual Regulation of Molecular Orbital and Interfacial Water on Nickel Phthalocyanine for Highly Selective H2O2 Synthesis. *Angewandte Chemie* **2026**, e1594313.
(22) Wang, Y.; Shi, R.; Shang, L.; Waterhouse, G. I.; Zhao, J.; Zhang, Q.; Gu, L.; Zhang, T. High-efficiency oxygen reduction to hydrogen peroxide catalyzed by nickel single-atom catalysts with tetradentate N2O2 coordination in a three-phase flow cell. *Angewandte Chemie International Edition* **2020**, *59* (31), 13057-13062.
(23) Xiao, C.; Cheng, L.; Zhu, Y.; Wang, G.; Chen, L.; Wang, Y.; Chen, R.; Li, Y.; Li, C. Super-coordinated Nickel N4Ni1O2 site single-atom catalyst for selective H2O2 electrosynthesis at high current densities. *Angewandte Chemie* **2022**, *134* (38), e202206544.
(24) Wang, Y.; Zhang, Z.; Zhang, X.; Yuan, Y.; Jiang, Z.; Zheng, H.; Wang, Y.-G.; Zhou, H.; Liang, Y. Theory-driven design of electrocatalysts for the two-electron oxygen reduction reaction based on dispersed metal phthalocyanines. *CCS Chemistry* **2022**, *4* (1), 228-236.
(25) Kattel, S.; Wang, G. A density functional theory study of oxygen reduction reaction on Me–N4 (Me = Fe, Co, or Ni) clusters between graphitic pores. *Journal of Materials Chemistry A* **2013**, *1* (36), 10790-10797.
